# Reversibly Crosslinked Polyurethane Fibres from Sugar‐Based 5‐Chloromethylfurfural: Synthesis, Fibre‐Spinning and Fibre‐to‐Fibre Recycling

**DOI:** 10.1002/cssc.202402067

**Published:** 2024-11-11

**Authors:** Niklas Warlin, Maria Nelly Garcia Gonzalez, Rafael N. L. de Menezes, Andras Karajos, Emma Olsson, Caroline Almqvist, Mahmoud Sayed, Smita V. Mankar, Nitin G. Valsange, Omar Y. Abdelaziz, Christian P. Hulteberg, Fredrik G. Bäcklund, Zengwei Guo, Nicola Rehnberg, Stefan Lundmark, Rajni Hatti‐Kaul, Patric Jannasch, Baozhong Zhang

**Affiliations:** ^1^ Department of Chemistry Centre for Analysis and Synthesis Lund University SE-221 00 Lund Sweden; ^2^ Department of Chemistry Stanford University Stanford 94305-5080 California United States; ^3^ Environmental and Energy Systems Studies Department of Technology and Society Lund University SE-221 00 Lund Sweden; ^4^ Division of Biotechnology Department of Chemistry Lund University SE-221 00 Lund Sweden; ^5^ Department of Chemical Engineering King Fahd University of Petroleum & Minerals Dhahran 31261 Saudi Arabia; ^6^ Interdisciplinary Research Center for Refining & Advanced Chemicals King Fahd University of Petroleum & Minerals Dhahran 31261 Saudi Arabia; ^7^ Division of Chemical Engineering Department of Process and Life Science Engineering Lund University SE-221 00 Lund Sweden; ^8^ Division Materials and Production Department of Polymers Fibers and Composites RISE Research Institutes of Sweden SE-43153 Mölndal Sweden; ^9^ Bona Sweden AB SE-200 21 Malmö Sweden; ^10^ Perstorp AB Perstorp Industrial Park SE-284 80 Perstorp Sweden

**Keywords:** Fibre spinning, Chemical recycling, Polyurethane, Bio-based molecules, Life cycle assessment

## Abstract

The development of recyclable crosslinked thermosetting fibres is a challenging research topic. In the present work, we have designed and synthesized polyurethane fibres from fructose‐derived 5‐chloromethylfurfural (CMF) and lignin‐derived monomeric phenols. The greenhouse gas emissions associated with the production of CMF showed comparable results to that of 5‐hydroxymethylfurfural (HMF), a high potential sugar‐based platform molecule. The wet‐spun biobased polyurethane fibres produced could be conveniently crosslinked using Diels–Alder chemistry to effectively enhance the glass transition temperature and mechanical properties. At a mildly elevated temperature (140 °C), the chemically crosslinked fibres could be effectively de‐crosslinked, which enabled complete separation from a mixture with poly(ethylene terephthalate) (PET) and cotton fibres. These results outline a potential strategy to design and fabricate new biobased fibres with reversible crosslinking, which may enable fibre‐to‐fibre recycling.

## Graphical abstract

Sugar‐derived 5‐chloromethylfurfural and lignin‐derived vanillin were used to synthesize and wet‐spin a reversibly crosslinked polyurethane fibre. The fibre was selectively recycled from a mixture of cotton and PET by heating them to reverse the crosslinking of the polyurethane in dimethylformamide. The resulting solution was filtered and directly re‐spun into another fibre.

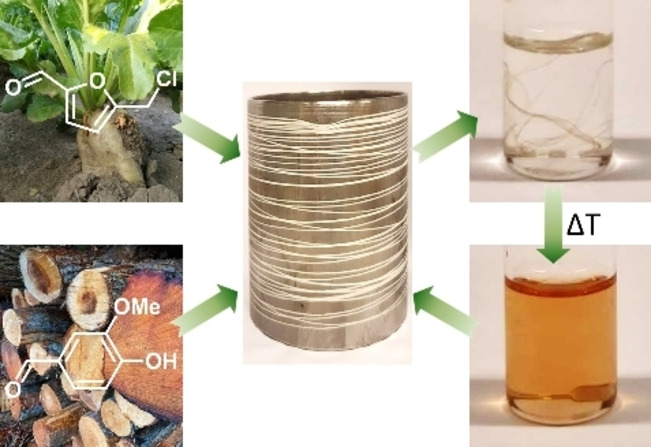

## Introduction

Today, polymer fibres are ubiquitous in society and industry (e. g. textiles, construction, automotives, medicine, etc.), with an annual production exceeding 100 million tons.[^1^, ^2^] Although most fibres on the market are thermoplastics (e. g., polyesters, polyolefins, polyamides and polyurethanes),[^1^, ^2^, ^3^, ^4^] thermosetting (chemically crosslinked) fibres have received growing attention due to their desirable mechanical properties[^5^, ^6^] and chemical and thermal stability.[^1^, ^7^] Unfortunately, chemically crosslinked fibres (just like other crosslinked polymer materials) frequently suffer from poor processability and recyclability.^[8]^ Therefore, the development of new thermosetting fibres with reversible crosslinking has become attractive. If the crosslinking bonds in the fibres are dynamic, or hydrolytically cleavable,^[9]^ re‐processing and recycling may be facilitated after the re‐collected fibres are subjected to de‐crosslinking under suitable physical/chemical conditions.[^10^, ^11^, ^12^, ^13^] Recently, thermosetting fibres with covalent adaptable networks based on oxime‐urethane structures have been reported with desirable mechanical properties and re‐processability.^[1]^ Thermally driven reversible Diels‐Alder reactions have also been demonstrated to be effective in developing reversible crosslinked materials.[^14^, ^15^, ^16^, ^17^]

Another environmental concern about polymer fibres is related to their dependence and consumption of non‐renewable fossil resources.[^18^, ^19^, ^20^] Therefore, there has been significant research in the development of sustainable fibres utilizing various bio‐sourced raw materials during the past decade.[^19^, ^21^] Among them, carbohydrates are particularly attractive biomass resources, which have been widely investigated as starting materials for biobased polymers (e. g. PLA, PET, PEF, and PTT) that are potentially applicable in textile fibres as well as other applications such as packaging, disposable tableware, cutlery, upholstery, etc.[^22^, ^23^, ^24^, ^25^] Recently, 5‐hydroxymethylfurfural (HMF) has been identified as an attractive sugar‐based platform molecule for developing sustainable polymers,[^26^, ^27^, ^28^, ^29^, ^30^, ^31^, ^32^, ^33^, ^34^, ^35^, ^36^, ^37^] despite that its instability at high temperatures or under acidic conditions may hinder its large‐scale production and utilization.[^38^, ^39^, ^40^] Recently, another sugar‐based analog to HMF, 5‐chloromethylfurfural (CMF), has attracted growing attention due to its desirable stability under acidic conditions, enabling it to be produced from low‐cost carbohydrates (e. g., cellulose, glucose, or raw biomass).[^41^, ^42^, ^43^, ^44^, ^45^, ^46^] More importantly, HMF and CMF contain furan groups that could enable reversible Diels‐Alder reactions, which may be desirable in the design and development of new reversibly crosslinked fibres. To our knowledge, the application of Diels‐Alder chemistry to recycle bio‐sourced thermosetting polymer fibres based on HMF or CMF has not been reported so far.

Herein, we report the synthesis of CMF from fructose‐based raw materials and a preliminary evaluation of its environmental aspects. The utilization of sugar‐based CMF in the preparation of reversibly crosslinked polyurethane fibres was then investigated. Recycling of the crosslinked fibres was achieved based on a retro‐Diels–Alder reaction at elevated temperature, followed by recovery of the de‐crosslinked polymers, which were subsequently re‐spun into fibres. In this way, we have initially demonstrated closed‐loop fibre‐to‐fibre recycling of the newly designed sugar‐based polymer.

## Results and Discussion

### Comparative Life Cycle Assessment (LCA) of Gram‐Scale CMF and HMF Synthesis

Recently, CMF has been identified as a potential alternative to the important biobased platform molecule HMF.^[41]^ However, the climate impact of CMF, particularly compared to that of HMF, remains to be unraveled. In this work, we preliminarily compared the Green House Gas (GHG) emissions for the production of CMF and HMF based on a cradle‐to‐gate LCA of several reported synthesis procedures performed at gram‐scale.[^39^, ^42^, ^47^, ^48^, ^49^] It should be noted that this was an early‐stage analysis aiming to provide a first insight into the possible crucial aspects of the synthesis of these molecules, and only considered synthesis procedures performed at a relatively small scale (<500 g). Therefore, the results should be considered with caution, and deeper analysis will be needed before drawing comprehensive conclusions regarding GHG emissions of industrial biobased HMF and CMF production (not yet fully realized), e. g., by including additional impact categories.

**Figure 1:**
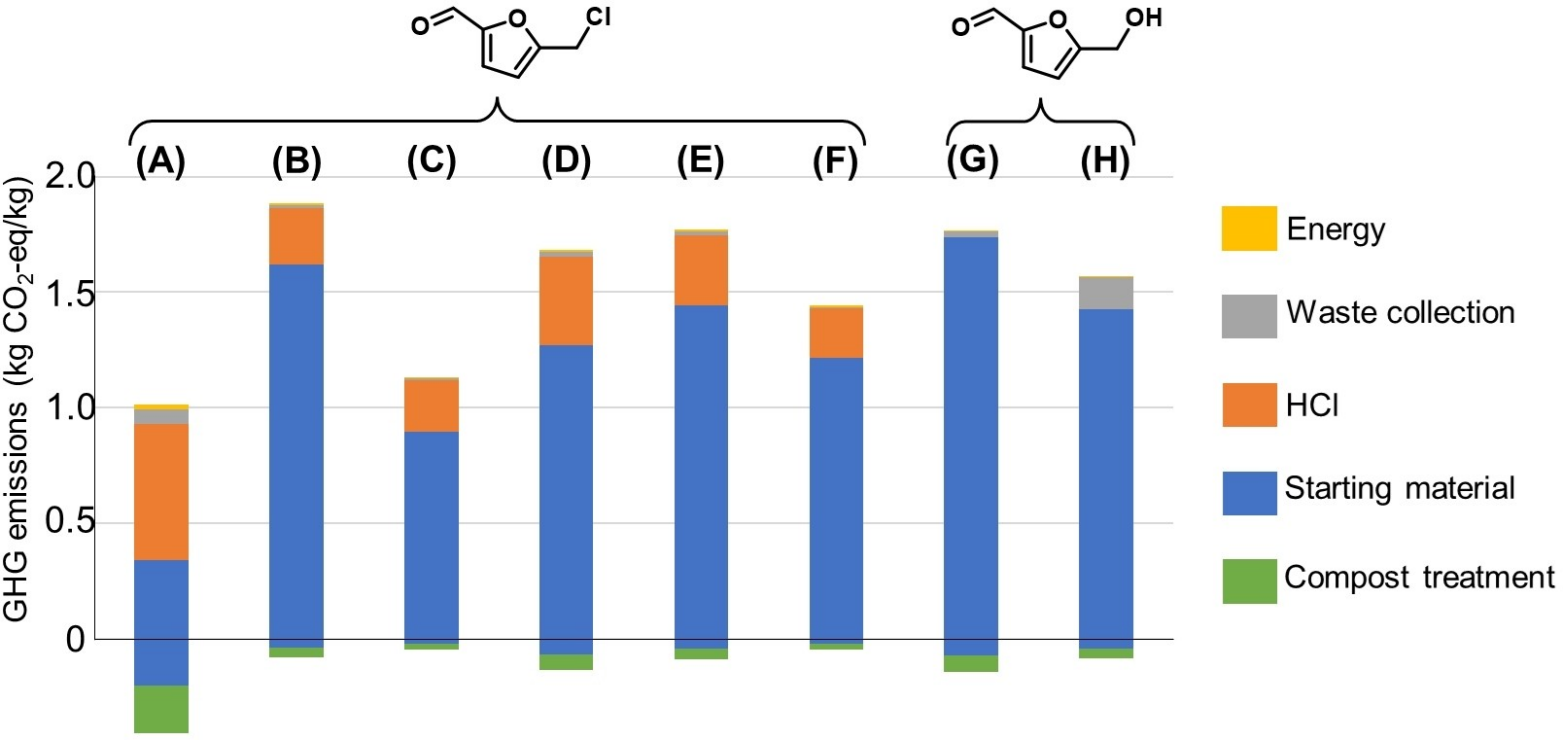
The GHG emissions as predicted by LCA of the synthesis of CMF (A−F) and HMF (G−H) from various carbohydrate biomass sources, including (A) used corn stover, (B) used glucose, (C)‐(D) used sucrose, and (E)‐(H) used fructose. Only the energy required to maintain the reaction temperature was considered. All the contributions of the synthesis are shown in Supplementary Table S5.

The GHG emissions for CMF synthesis in the current scale (gram scale) were estimated to be in the range of 1.01–1.89 kg CO_2_‐eq/kg CMF (Cases A–F, Figure 1 and Supplementary Table S5). Among all these investigated processes, the lowest (1.01 kg CO_2_‐eq/kg CMF) and highest (1.89 kg CO_2_‐eq/kg CMF) GHG emissions were obtained using corn stover (Case A, Supplementary Table S5) and glucose (Case B, Supplementary Table S5) as the starting materials, respectively. These two values coincide with the lowest and highest GHG emissions for the corresponding starting material production (0.15–1.1 kg CO_2_‐eq/kg starting material, respectively),[^50^, ^51^] indicating that the choice of starting material is crucial for the final result. Moreover, Case A (Figure 1) was also the only case where the largest contribution did not come from the starting material. The GHG emissions for HMF synthesis were estimated to be in the range of 1.57 – 1.77 kg CO_2_‐eq/kg HMF (Cases G−H, Figure 1). According to this result, the GHG emissions of CMF and HMF were in a similar range, depending on the type of starting materials and synthetic procedures. The choice and processing of the starting materials were especially important from an environmental perspective.

Particularly when fructose was used as the starting material for CMF and HMF production (Cases E–H, Figure 1, Supplementary Table S5), similar GHG emissions were estimated for both molecules.

It should be noted that the calculated data values in Figure 1 do not include the energy used to heat the solvents, because the choice and amount of solvent were not optimized in many cases and are thus not meaningfully comparable. However, if this factor was taken into consideration by just using the reported values in the publications, significantly higher GHG emissions were obtained in all cases (1.48–3.26 kg CO_2_ eq/kg, Supplementary Tables S6–7), with Case A being the worst (3.26 kg CO_2_ eq/kg). This confirms the significant impact of the solvent used for the synthesis.

Sensitivity analysis (based only on the energy required to maintain constant reaction temperature, base case, Supplementary Tables S8 and S9) showed insignificant differences between the Swedish and EU electricity production, although the contribution values using EU electricity were slightly higher. The GHG emissions differed insignificantly with respect to the base case among the different synthesis procedures (details in Supplementary Tables S8 and S9). The rest of the contributions remained the same.

Overall, our preliminary LCA results suggest that CMF could be an environmentally friendly alternative to the more widely studied analogue, HMF. The exact synthetic procedure, particularly the choice and volume of the organic solvent used for the synthesis, shows a significant impact on their GHG emissions, which needs to be carefully considered to achieve a sustainable production.

### Monomer Synthesis

As indicated above, there are many different synthetic pathways to obtain CMF from various biomass resources (e. g., cellulose, fructose, glucose, and sucrose),[^52^, ^53^] and these synthetic methods will have a significant environmental impact. However, our main focus here is to explore the valorization of CMF and various lignin‐based molecules in polymer production. Consequently, the most convenient production method of CMF was chosen by a facile S_N_2 reaction of HMF with HCl at room temperature. The high purity of the obtained CMF was confirmed by ^1^H nuclear magnetic resonance (NMR) spectroscopy (Supplementary Figure S2 and Figures S5–9).

Both fructose‐based HMF (synthesized in this work) and commercial fossil‐based HMF were used for the synthesis of CMF; the latter showed a slightly higher yield and less black humin formation (76 % and 62 % yields when using fossil‐ and fructose‐based CMF, respectively). However, there was no significant difference in the ^1^H NMR spectra of the CMF produced from the two sources (Supplementary Figure S2).

**Scheme 1:**
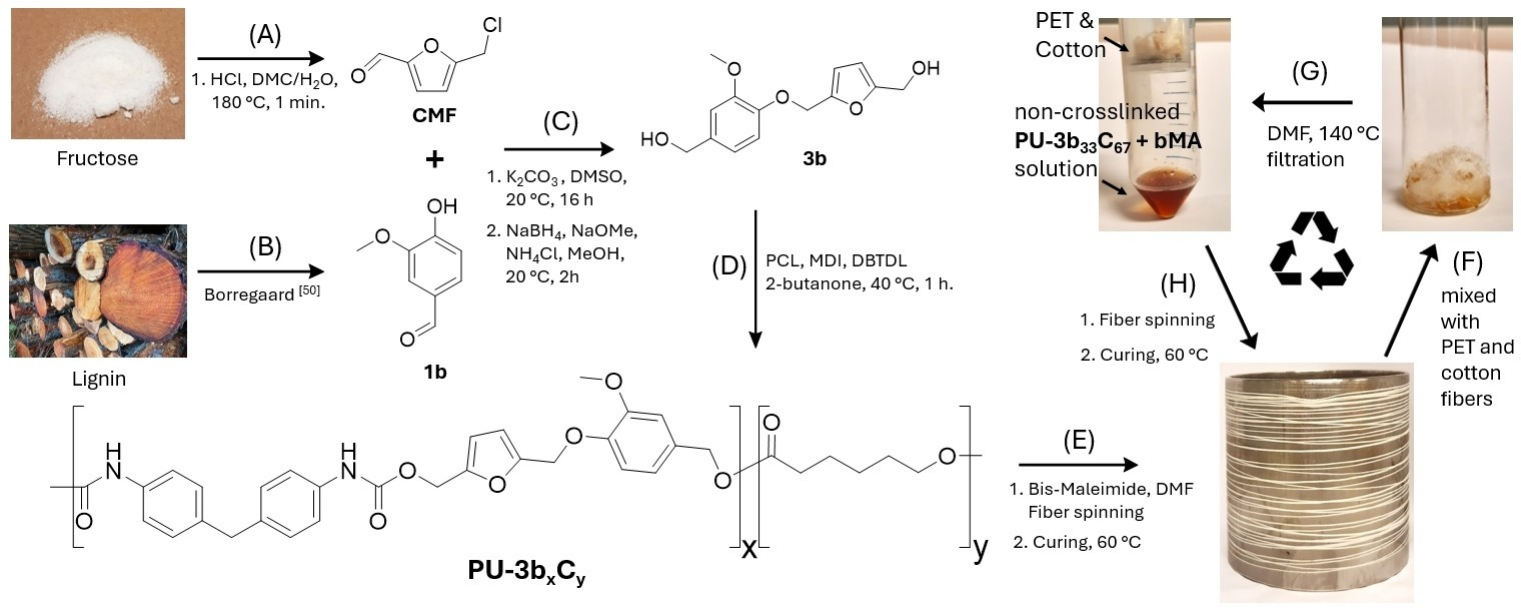
**(A)** Synthesis of CMF from fructose. Reaction conditions: Step 1. Fructose (1 eq.) HCl (0.23 M), dimethyl carbonate/H_2_O, 180 °C, 1 min. Step 2. HMF (1 eq.), HCl/CH_2_Cl_2_, 20 °C, overnight. **(B)** Synthesis of vanillin (1b) from lignin by Borregaard.^[55]^ **(C)** Step 1. Synthesis of bio‐based aldehydes (**2 a–c**). Reaction conditions: **1 a–c** (1 eq.), CMF (1 eq.), K_2_CO_3_ (1 eq.), DMSO, 20 °C, overnight. Step 2. Synthesis of bio‐based diols (**3 a–c**). Reaction conditions: **2 a–c** (1 eq.), NaBH_4_ (1.5–2.1 eq.), NaOMe (0.1 eq.), NH_4_Cl (sat.), MeOH, 20 °C, 2 h. **(D)** Copolymerization of Monomer **3 b** with polycaprolactone (PCL) and methylene diphenyl diisocyanate (MDI), yielding a series of copolymers **PU‐3b_x_C_y_** (y=67, 50, and 33, denoting the feed weight fraction of Monomer **3 b** with respect to PCL). **PU‐C** is the polyurethane synthesized using PCL and MDI without Monomer **3 b**. PU‐3b is synthesized using Monomer **3 b** and MDI without PCL. Reaction conditions: PCL (0.01*x eq.), 3b (0.01*y eq.), MDI (1.05 eq.), dibutyltin dilaurate (DBTDL, 0.002 eq.), 2‐butanone, 40 °C, 1 h. **(E)** Step 1. Fibre spinning of PU‐3b_33_C_67_ with bis‐maleimide into a polyurethane fibre. Step 2. Fibre is cured at 60 °C to trigger a Diels‐Alder reaction between the furan units in the polymer and bis‐maleimide. **(F) PU‐3b_33_C_67_g** fibre cut into pieces and mixed with PET and cotton fibres wetted with DMF. **(G)** De‐crosslinking of **PU‐3b_33_C_67_g** fibre in DMF at 140 °C, and then the solution separated from PET and cotton fibres by filtration. **(H)** New fibres spun from the **PU‐3b_33_C_67_g** solution.

The obtained CMF was subjected to a straightforward two‐step synthetic procedure using three lignin‐derived monomeric phenols (**1 a–c**, Scheme 1) to yield three di‐aldehydes (**2 a–c**), and then the corresponding diols (**3 a–c**). The first step was an S_N_2 reaction under mild conditions (performed at room temperature overnight with the mild base K_2_CO_3_). The impact of the solvent on the reaction between CMF and vanillin (**1 b**) was investigated by evaluating the conversion of CMF (Supplementary Table S10) in the crude reaction mixtures (after solvent evaporation) according to the ^1^H NMR spectra. As a result, the highest CMF conversion was achieved using DMSO as the solvent, followed by DMF (100 and 86 %, respectively), which could be related to their ability to dissolve K_2_CO_3_ and to facilitate the deprotonation of the phenol group in **1 b**.^[54]^ Other solvents resulted in relatively low conversions (<40 %). Methanol resulted in only 37 % conversion, possibly because the nucleophile can form hydrogen bonds with the solvent (methanol) and thus reduce its reactivity. Other solvents with relatively low polarity (e. g., acetone, tetrahydrofuran (THF), acetonitrile, CH_2_Cl_2_, and ethyl acetate) gave even lower CMF conversion (0–12 %) due to the low solubility of K_2_CO_3_. Low conversion (9 %) was also observed when water was used as the solvent, likely due to the poor aqueous solubility of CMF. Therefore, DMSO was chosen as the optimized solvent for the synthesis of dialdehydes **2 a–c**. In addition to the solvent effect, we have also investigated the effects of the starting materials for this reaction. Both bio‐based CMF that we synthesized and bio‐based vanillin from Borregaard ASA were used for the synthesis, and then compared to the results when fossil‐based CMF and fossil‐based vanillin were used. As shown in Supplementary Table S11 and Figure S3, there was no significant difference in the yield and purity of **2 a‐b** when using different starting materials.

The second synthetic step to prepare diols **3 a‐**‐**c** was a straightforward reduction of the aldehyde groups in **2 a–c** using NaBH_4_. A catalytic amount of NaOMe was used to prevent the undesirable methanolysis of NaBH_4_.[^56^, ^57^] For **3 a** and **3 c**, reactions in methanol proceeded smoothly. For **3 b**, the reaction in methanol suffered from an undesirable formation of methyl acetal as a side reaction (Supplementary Figure S4), so the reaction was carried out in acetonitrile instead. After the reaction, the excess NaBH_4_ and NaOMe catalyst was quenched by an aqueous NH_4_Cl solution, and the resulting crude products were conveniently purified by extraction with DCM yielding **3 a–c** as light yellow crystals.

### Synthesis and Characterization of Thermoplastic Copolyurethanes

In many practical applications (e. g., coatings, fibres, adhesives, and medical implants) the backbones of polyurethanes frequently contain a soft segment (e. g., a long flexible diol) to improve the mechanical properties.[^58^, ^59^] In this work, the flexible polyester diol polycaprolactone (PCL, *M*_n_=400 Da), was employed for polyurethane synthesis together with MDI and Monomer **3 b** (Scheme 1D). This reaction was usually rapid and exothermic, resulting in rapidly increased temperature and viscosity. To prevent this uncontrollable situation, we adopted a procedure by adding the catalyst DBTDL dropwise while adding more solvent. This method effectively controlled the temperature and viscosity of the reaction mixture, resulting in complete consumption of the isocyanate (monitored by Fourier‐transform infrared spectroscopy, FTIR) while minimizing undesirable branching side reactions such as allophanate or isocyanurate formation which may cause crosslinking and gelation.[^60^, ^61^] Afterward, the reaction was quenched by dibutylamine to remove any possible residual toxic isocyanates. The obtained crude polymer was purified by precipitation, yielding **PU‐3b_x_C_y_** as pale‐yellow solids. The chemical structure of the polymers was determined by ^1^H NMR spectroscopy (Supplementary Figure S40–50), and the molecular weights were determined by size exclusion chromatography (SEC, Table 1).

**Table 1:** Molecular and thermal properties of the obtained thermoplastic polyurethanes.

	SEC		TGA			DSC	DMA	
Polymer	*M*_n_^[a]^ (kDa)	*M*_w_^[a]^ (kDa)	*T*_5_^[b]^ (°C)	*T*_d_^[b]^ (°C)	CY^[b]^ (%)	*T*_g_^[c]^ (°C)	*T*_g_^[d]^ (°C)	E’^[e]^ (MPa)
PU‐3b	47.4	111	200	212|265|312|363	32.2	110	–	–
PU‐3b_67_C_33_	49.4	96.7	218	227|303	32.5	86	77	1910
PU‐3b_50_C_50_	30.3	90.9	225	230|318	27.3	69	46	2120
PU‐3b_33_C_67_	38.0	102	236	233|327	20.0	53	24	2146
PU‐C	62.9	157	272	325	8.7	17	–	–

^[a]^ *M*_n_ and *M*_w_ were measured by size exclusion chromatography (SEC) with DMAc containing 5 g/L LiBr. ^[b]^ *T*_5,_ *T*_d_ and char yield (CY) values were measured by Thermogravimetric Analysis (TGA), as the temperature with 5 % mass loss, the temperature with the local maximum mass loss (from the derivative weight curve), and the residual mass after heating the polymer to 600 °C, respectively. ^[c]^ *T*_g_ was measured by differential scanning calorimetry (DSC) and taken from the second heating curves. ^[d]^ *T*_g_ measured by dynamic mechanical analysis (DMA) as the maximum in E’’ curves. ^[e]^ Storage modulus taken at 30 °C below *T*_g_.

**Figure 2:**
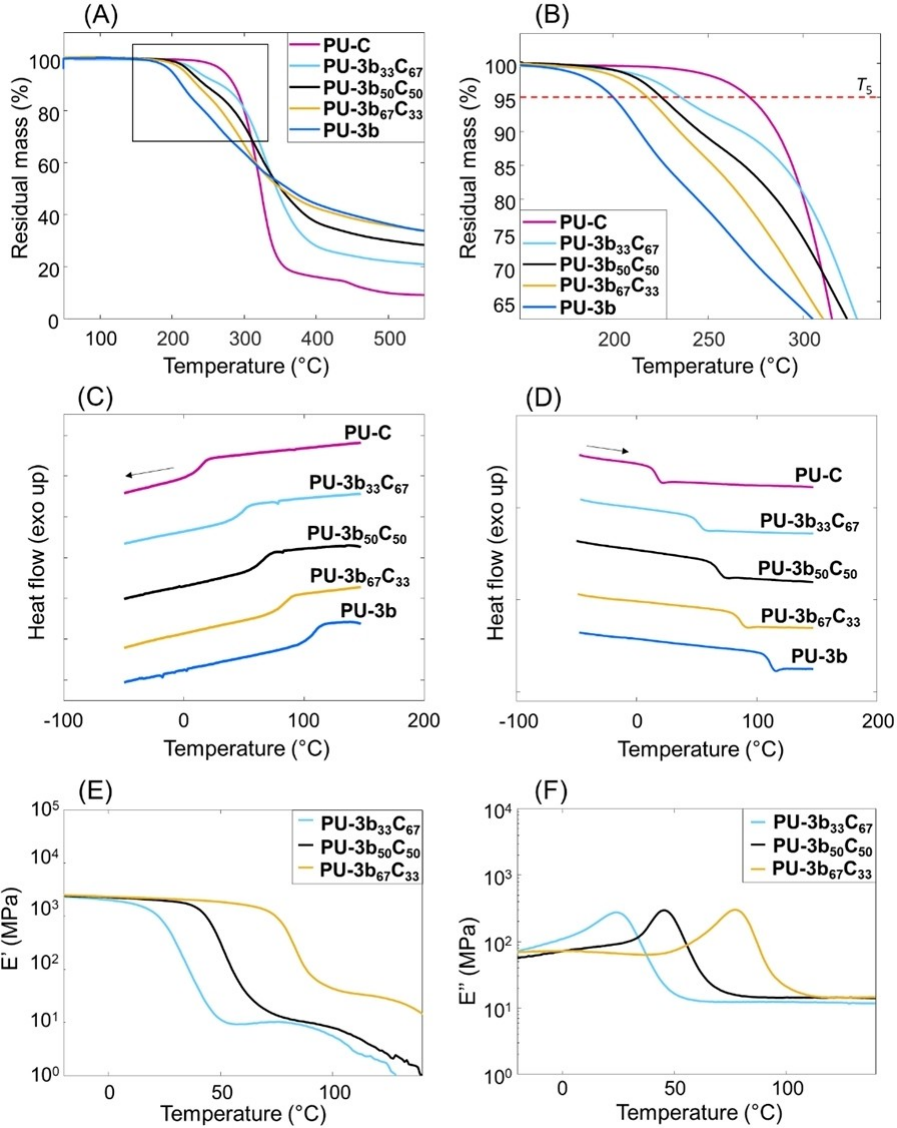
(A) TGA thermograms of the series of **3 b**‐based polyurethanes. (B) zoomed‐in window of the *T*_5_ range of the obtained polyurethanes. DSC 1^st^ cooling (C) and 2^nd^ heating curves (D) of the series of thermoplastic copolyurethanes **PU‐3b_x_C_y_** (y=33, 50, and 67), in comparison with two homopolymers **PU‐3 b** and **PU‐C**. The arrows on top indicate whether they are heating or cooling. DMA storage (E) and loss moduli (F) for the obtained series of thermoplastic copolyurethanes.

The thermal properties of the series of copolyurethanes (including the two homopolymers **PU‐3 b** and **PU‐C**) were investigated by TGA and DSC (Figure 2A–D, Table 1). All polymers were dried for 2 h at 20 °C above their respective *T*_g_ to remove any trace of solvent residues from the sample. All polymers showed *T*_5_≥200 °C with a clear decreasing trend with the increased content of **3 b** (from **PU‐C** to **PU‐3 b**, Table 1). This indicated that the urethane units formed between monomer **3 b** and MDI were less thermally stable compared to the urethane units formed by PCL and MDI. This was supported by the observation that **PU‐C** exhibited a single thermal decomposition maximum at 325 °C (Supplementary Figure S51B), most likely due to the cleavage of the urethane unit formed by PCL and MDI. Hence, the first degradation rate maximum at 212–233 °C for **PU‐3 b** and all copolymers could be attributed to the cleavage of the urethane units formed by **3 b** and MDI. This observation was consistent with the generally lower stability of aromatic urethane bonds compared to aliphatic ones.[^62^, ^63^, ^64^] Furthermore, the char yields (CY) of the three copolymers increased with the content of **3 b**, which was consistent with the increased aromatic contents.^[65]^ Notably, **PU‐3 b** did not show higher CY than **PU‐3b_67_C_33_**, despite its high content of aromatic **3 b** structures. The series of copolyurethanes were further analyzed by DSC (Figures 2C,D and Table 1). As shown in the second heating curves (Figure 2D), no melting endotherm was observed for all these polymers, indicating their amorphous nature. A single *T*_g_ was observed for all copolymers, which indicated good mixing between the hard and soft segments.^[66]^ The observed *T*_g_ values increased with the increased content of rigid **3 b** structure, from 17 °C (**PU‐C**) to 110 °C (**PU‐3 b**).

DMA was used to further analyze the thermo‐mechanical properties of the series of copolyurethanes (**PU‐3b_33_C_67_**, **PU‐3b_50_C_50_**, and **PU‐3b_67_C_33_**). As a result, (Figure 2E,F), a characteristic rubbery plateau was observed for all copolymers after a two‐orders‐of‐magnitude drop at the *T*_g_, showing that these polymers were sufficiently long to form stable entanglements. Furthermore, the materials displayed storage moduli of 1.9–2.1 GPa at the glassy plateau (30 °C below *T*_g_), which decreased to 9–38 MPa at the rubbery plateau (Supplementary Table S12). The moduli at the rubbery plateau increased with the relative content of **3 b**, which was consistent with the increased concentration of hard segments.^[67]^ The *T*_g_ values (the maximum of the loss modulus curve in Figures 2F) increased with increasing content of **3 b**, which was generally consistent with the trend shown by the DSC results (Table 1). Some disparity between the absolute values was observed, which is a common observation since the two methods (DMA and DSC) are measuring different physical phenomena.[^68^, ^69^, ^70^, ^71^, ^72^]

### Reversible Crosslinking by Diels–Alder Reaction

The potential to crosslink the obtained thermoplastic polyurethanes by a Diels–Alder reaction of the furan groups in the polymers was investigated. The three copolymers (i. e., **PU‐3b_33_C_67_**, **PU‐3b_50_C_50_**, and **PU‐3b_67_C_33_**) were dissolved in THF and reacted with an equimolar amount of bis‐maleimide at 60 °C for 24 h (Supplementary Scheme S1) to form insoluble swollen gels (i. e., **PU‐3b_33_C_67_g**, **PU‐3b_50_C_50_g**, and **PU‐3b_67_C_33_g**, where the letter g stands for “gel” in this nomenclature). TGA measurements of the dried gels indicated similar thermal stability (*T_5_*) after crosslinking (Supplementary Table S13), which could be due to the fact that the least thermally stable units (the urethane groups) were unaffected by the crosslinking.^[64]^ An increased *T*_g_ after crosslinking was also observed by conventional DSC measurements during the second heating cycle (Supplementary Figure S52). However, it was suspected that Diels–Alder or retro‐Diels–Alder reactions might have occurred during the normal DSC scans, which may have affected the measured *T*_g_ values. Therefore, a more sophisticated special DSC measurement was conducted with in total 15 heating/cooling cycles. The result of **PU‐3b_33_C_67_g** is shown in Supplementary Figure S53A. All the heating cycles (cycles with odd numbers) were carried out with a heating rate of 10 °C/min.

During three of the cooling cycles (Cycle 4, 8, and 12), the samples were isothermally cured at 60 °C for 10 h before further cooling, in order to progressively crosslink the polymer further by the Diels–Alder reaction. During the other four cooling cycles (Cycle 2, 6, 10, 14), the samples were cooled down normally without curing (to serve as references). As a result, the *T*_g_ values observed during the heating cycles after the curing process (Cycle 5, 9, 13) were significantly higher than those observed during the conventional heating cycles (Cycle 3, 7, 11, 15). This confirmed the different degrees of crosslinking during the heating/cooling cycles, which were caused by the Diels–Alder and retro‐Diels–Alder reactions respectively. Furthermore, the *T*_g_ values were highly consistent during the heating cycles (Cycle 5, 9, 13, as well as Cycle 3, 7, 11, 15), which indicated the thermal reversibility of the crosslinking/decrosslinking reactions. Similar DSC measurements were conducted for the other two copolymers **PU‐3b_50_C_50_g** and **PU‐3b_67_C_33_g**. However, no significant increase of the *T*_g_ was observed during the heating cycles after the curing process (Supplementary Table S14). This could be attributed to the fact that these two polymers have higher *T*_g_ than the curing temperature (60 °C), which restricted the chain mobility at 60 °C, and thus lowered the rate of the Diels–Alder reactions (Supplementary Figure S53).

### Fibre Spinning and Analysis

**Figure 3:**
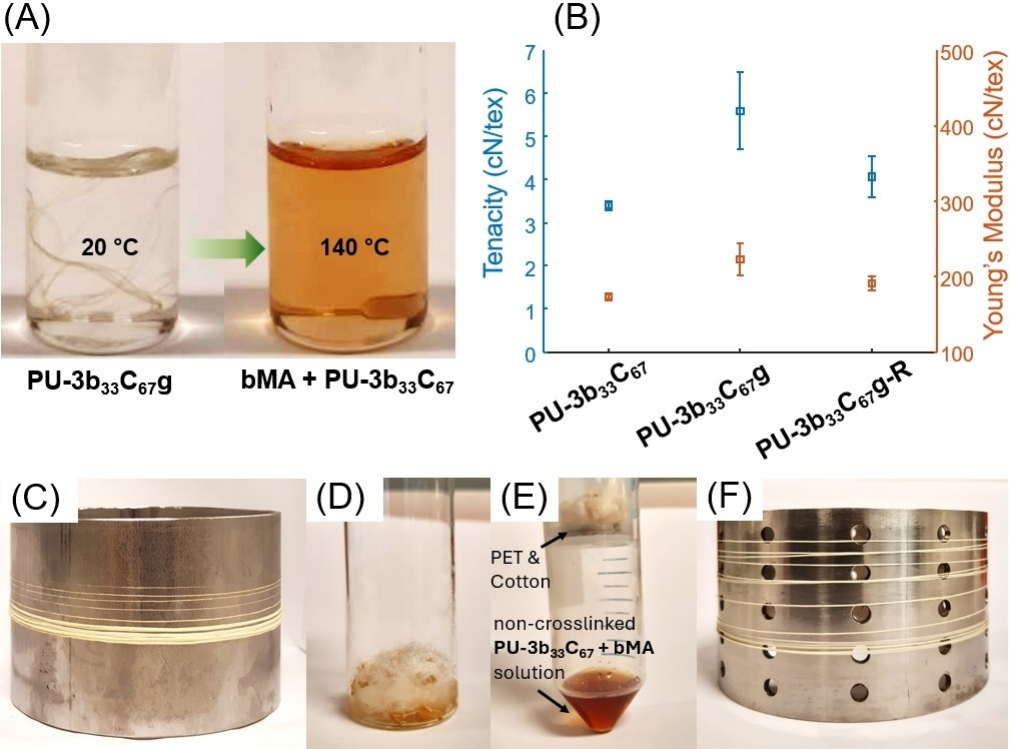
(A) De‐crosslinking of **PU‐3b_33_C_67_g** by the retro‐Diels–Alder reaction in DMF at 140 °C to form a solution of bis‐maleimide (bMA) and **PU‐3b_33_C_67_**. The crosslinked polymer was completely dissolved after 8 min at 140 °C. (B) Mechanical properties of the fibres before and after crosslinking. Standard deviation is indicated by error bars. (C) Collected fibres of crosslinked **PU‐3b_33_C_67_g** after wet spinning and curing. (D) Mixed fibres of crosslinked **PU‐3b_33_C_67_g**, PET, and cotton. The fibres are wetted by DMF. (E) The **PU‐3b_33_C_67_** solution separated from PET and cotton fibres. (F) Re‐produced **PU‐3b_33_C_67_g‐R** fibre using the recycled spinning solution.

The non‐crosslinked **PU‐3b_33_C_67_** and crosslinked **PU‐3b_33_C_67_g** were subjected to wet spinning experiments. For comparison, the polymer without furan unit (i. e. **PU‐C**, Scheme 1) was also subjected to the same fibre spinning investigations. Prior to dissolving in DMF, **PU‐3b_33_C_67_g** was heated to 140 °C for 5 minutes to de‐crosslink the polymer chains and facilitate solubilization. **PU‐C, PU‐3b_33_C_67_** and **PU‐3b_33_C_67_g** were then dissolved in DMF and extruded through a spinneret with 12 holes (D=80 μm) into a coagulation bath with deionized water. The multi‐filament fibres were successfully collected at pick‐up speeds of 5 and 6 m/min. Increasing the pick‐up speed to 10 m/min resulted in fibre breakage. TGA and DSC analysis of the crosslinked **PU‐3b_33_C_67_g** fibre showed similar thermal properties to the polymer powder (Supplementary Figures S55–56 and Supplementary Table S15), confirming that the wet spinning process did not significantly affect thermal properties. To gain further insight into the de‐crosslinking process, **PU‐3b_33_C_67_g** was swollen in DMSO‐*d*_6_ and then heated to 140 °C. The obtained gel was dissolved completely in 10 minutes, indicating de‐crosslinking (Figure 3A). The obtained solution (containing the regenerated non‐crosslinked polymer and bMA) was then directly analyzed by ^1^H NMR spectroscopy (Supplementary Figure S57C), which showed all the peaks for the original non‐crosslinked polymer (**PU‐3b_33_C_67_**, Fig. S57B Supplementary) and crosslinking agent bMA (Supplementary Figure S57A). This further confirmed a complete clean de‐crosslinking based on retro‐Diels‐Alder chemistry without any noticeable side reactions.

Recently, chemical recycling has emerged as a promising way to recycle complex textile and other polymer materials, which mainly includes the chemical/physical processes based on alcoholysis, solvolysis, pyrolysis, or solvent extraction.[^73^, ^74^, ^75^, ^76^, ^77^, ^78^, ^79^] In this work, the recyclability of crosslinked **PU‐3b_33_C_67_g** fibre in a mixture with other common textile fibres was investigated. First, the obtained crosslinked **PU‐3b_33_C_67_g** fibre was cut into pieces (approximately 1 cm in length) and mixed with PET and cotton fibres (both commercially available) and suspended in DMF (Figure 3C,D). The mixture was heated to 140 °C with mechanical stirring for 10 minutes, and then separated by gravity filtration. The obtained solution (containing non‐crosslinked regenerated polymer and the crosslinking agent bMA) was directly subjected to the same wet‐spinning and curing process, yielding the recycled crosslinked fibre **PU‐3b_33_C_67_g‐R** (Figures 3E,F, where the letter R stands for recycled). According to TGA and DSC measurements, the thermal properties (*T*_g_ and thermal stability) of this recycled fibre were similar to those of the fully crosslinked **PU‐3b_33_C_67_g** fibre (Supplementary Figure S60 and Supplementary Table S14–15).^1^H NMR spectroscopy also showed that the chemical composition of the re‐produced fibre **PU‐3b_33_C_67_g‐R** (Fig. S57D, ESI) was consistent with that of the original fibre **PU‐3b_33_C_67_g** (Figure S57C, ESI).

The mechanical properties of the crosslinked fibre (*i. e*. **PU‐3b_33_C_67_g**) were significantly enhanced after crosslinking. As shown in Figure 3B and Table S16 (expected, the crosslinked fibre **PU‐3b_33_C_67_g** showed significantly higher tenacity and Young′s modulus (5.6 cN/tex and 222.5 cN/tex, respectively) compared to that of the non‐crosslinked fibre **PU‐3b_33_C_67_** (3.4 cN/tex and 173.3 cN/tex, respectively). The recycled fibre (**PU‐3b_33_C_67_g‐R**) showed slightly lower tenacity than the fully crosslinked fibres but higher than the non‐crosslinked fibres (4.1 cN/tex). This may be explained by the existence of trace amounts of DMF in the recycled fibres. Finally, all these obtained fibres (**PU‐3b_33_C_67_**, **PU‐3b_33_C_67_g** and **PU‐3b_33_C_67_g‐R)** presented higher Young′s Modulus than the fibre produced by using **PU‐C** (the polymer without using monomer **3 b**, Scheme 1), whereas other mechanical properties (specially for the fibres obtained at draw ratio of 1.2) were somewhat similar.

## Conclusions

We have designed and synthesized new polyurethane fibres using renewable resources, including a sugar‐based platform molecule, CMF, and other potential lignin‐based molecules. Preliminary LCA results based on gram scale synthesis procedures indicated comparable environmental impacts of CMF as that of a more intensively studied sugar‐based analog, HMF. The LCA results also revealed the importance of starting materials and solvent amount for the gram scale synthesis process of CMF in terms of GHG emissions. A series of three rigid diols with 0–2 methoxy groups (per phenyl) were conveniently synthesized under mild conditions using CMF and three lignin‐based monomeric phenolic aldehydes. The diol made from vanillin and CMF were used to prepare a series of thermoplastic polyurethanes with high‐molecular weights (up to 62.9 kDa), relatively high thermal stability, and tunable *T*_g_s (17–110 °C, depending on the copolymer composition). We have also demonstrated that the polymers could be conveniently wet‐spun into fibres, and their *T*_g_ and mechanical properties could be improved by crosslinking the furan moieties with bis‐maleimide at 60 °C. This crosslinking process was completely reversible at 140 °C, which enabled effective isolation of polyurethane thermoplastic from a mixture of PET and cotton fibres. The recycled polyurethane in solution could also be conveniently spun into fibres again, which demonstrated its potential in fibre‐to‐fibre recycling.

## Conflict of Interests

The authors declare no conflict of interest.

1

## Supporting information

As a service to our authors and readers, this journal provides supporting information supplied by the authors. Such materials are peer reviewed and may be re‐organized for online delivery, but are not copy‐edited or typeset. Technical support issues arising from supporting information (other than missing files) should be addressed to the authors.

Supporting Information

## Data Availability

The data that support the findings of this study are available from the corresponding author upon reasonable request.
