# Supplementary material for: Reversibly Crosslinked Polyurethane Fibres from Sugar‐Based 5‐Chloromethylfurfural: Synthesis, Fibre‐Spinning and Fibre‐to‐Fibre Recycling
Source: ChemSusChem. 2024 Nov 11;18(4):e202402067. doi: 10.1002/cssc.202402067 (PMC11826136; doi:10.1002/cssc.202402067)
Supplement: Supplementary file 1 — Supporting Information [file CSSC-18-e202402067-s001.pdf]

ChemSusChem

Supporting Information

Reversibly Crosslinked Polyurethane Fibres from Sugar-Based 5-Chloromethylfurfural: Synthesis, Fibre-Spinning and Fibre-to-Fibre Recycling

Niklas Warlin, Maria Nelly Garcia Gonzalez, Rafael N. L. de Menezes, Andras Karajos, Emma Olsson, Caroline Almqvist, Mahmoud Sayed, Smita V. Mankar, Nitin G. Valsange, Omar Y. Abdelaziz, Christian P. Hulteberg, Fredrik G. Bäcklund, Zengwei Guo, Nicola Rehnberg, Stefan Lundmark, Rajni Hatti-Kaul, Patric Jannasch,* and Baozhong Zhang*

Supporting Information
©Wiley-VCH 2021
69451 Weinheim, Germany

Reversibly Crosslinked Polyurethane Fibres from Sugar-based 5-Chloromethylfurfural: Synthesis, Fibre-spinning and Fibre-to-fibre Recycling

Niklas Warlin,[a],[b] Maria Nelly Garcia Gonzalez,[c] Rafael N. L. de Menezes,[a] Andras Karajos,[a] Emma Olsson,[a] Caroline Almqvist,[a] Mahmoud Sayed,[d] Smita V. Mankar,[a] Nitin Valsange,[a] Omar Y. Abdelaziz,[e],[f] Christian P. Hulteberg,[g] Fredrik G. Bäcklund,[h] Zengwei Guo,[h] Nicola Rehnberg,[a],[i] Stefan Lundmark,[j] Rajni Hatti-Kaul,[c] Patric Jannasch*[a] and Baozhong Zhang*[a].

Table of Contents

Experimental Procedures.. 2
Calculations.. 5
Results and Discussion.. 7
References 45
Author Contributions... 46

Experimental Procedures

Materials

5-Hydroxymethylfurfural (5-HMF, 98%) was purchased from Nanjing Confidence Chemical Co., Ltd. Dibutyltin dilaurate (DBTDL, Metatin katalysator 712 ES) was purchased from Dow Chemicals. Methanol and dimethylformamide (DMF) were supplied by VWR Chemicals. Silica gel 60 (0.015 - 0.040 mm), *tert*-butyl methyl ether (TBME, HPLC grade, 99.8%), tetrahydrofuran (THF, 99.9%), dimethyl carbonate (DMC, 99%), NH_4Cl (>99.5%), syringaldehyde (98%), vanillin (98%), 4-hydroxybenzaldehyde (98%), Celite 545, dichloromethane (DCM, 99.9%), HCl (37 %), 2-butanone (99 %), methylene diphenyl diisocyanate (MDI, 98%), LiBr (99%), DMAc (99 %), DMSO-d_6 (99.9% atom D) were purchased from Sigma–Aldrich. $NABH_4$ (95%) was purchased from Riesel de Haën. Dibutylamine (DBA, >99%) was purchased from BASF. Na_2SO_4 (anhydrous, >95%) was purchased from Honeywell. NaOMe (>95%) was purchased from Acros Organics. PCL (CAPA2043, M_n = 400 Da) was obtained from Perstorp AB. Bio-based vanillin derived from Norway spruce (*Picea abies*) was obtained from Borregaard (Borregaard ASA, Sarpsborg, Norway).

Methods

^{1}H and ^{13}C NMR measurements were performed on a Bruker DR X400 spectrometer at 400.13 MHz and 100.61 MHz, respectively, and chemical shifts were reported as δ values (ppm). HRMS was taken on a Micromass QTOF mass spectrometer (ESI). SEC measurements for the polyurethanes were carried out with an Agilent 1100/1200 Infinity HPLC System, equipped with three columns (GPC column PSS GRAM 3000 Å, 10 µm; GPC column PSS GRAM 1000 Å, 10 µm; GPC column PSS GRAM 30 Å, 10 µm) connected in sequence at 40 °C. Measurements were performed using DMAc with LiBr (5 g L^{-1}) at a flow rate of 1 mL min^{-1}. Calibration was carried out with ReadyCal-Kit poly(methyl methacrylate) standards M_p = 202–2 200 000 Da. Fourier transform infrared (FTIR) spectra were measured with an attenuated total reflection (ATR) setup using a Bruker Alpha FT-IR spectrometer. The polyurethanes were taken directly from the reaction solution and placed on the cell. For all samples 16 successive scans over the range of 400–4000 cm^{-1} were recorded. All polymers were dried for 2 h at 20 °C above their respective T_g to remove any trace of solvent residues from the sample before any thermal analysis. TGA measurements were carried out on a TA instruments TGA Q500. The samples were heated from 50–600 °C with a heating rate of 10 °C min^{-1}. DSC measurements were performed on a DSC Q2000 analyzer from TA instruments. Data for **PU-3a**-**c** and **PU-3b$_x$C$_y$** was recorded from -50 to 180 °C with a heating rate of 10 °C min^{-1}, and T_g was determined from the second heating cycle. Data for **PU-3b$_x$C$_y$** was recorded from -70 to 180 °C with a heating rate of 10 °C min^{-1}, and T_g was determined from the heating cycles. Annealing was performed at 60 °C for 10 hours during every second cooling cycle. The polyurethanes were made into films (17.5 × 5 × 1 mm) for dynamic mechanical analysis (DMA). PU films were made by hot pressing the polymers at 30 °C above their respective T_g. The mold was fixed in the press and the polymer was allowed to melt for 1 min, after which the pressure was increased to 3 kg/cm^2 for 1 min, 6 kg/cm^2 for 1 min, 10 kg/cm^2 for 1 min and finally 20 kg/cm^2 for 1 min. DMA measurements were performed in a stretching mode using TA instruments Q800 analyzer. The measured samples were heated from -50 °C to 150 °C at a heating rate of 3 °C min^{-1} and frequency of 1 Hz with a strain of 0.05%. Tensile testing was performed on a Vibrodyn/Vibroscope machine from Lenzing Technique GmbH & Co KG, Austria. The testing speed was 20 mm/min and the gauge length was 20 mm. The crosslinked **PU-3b$_{33}$C$_{67}$g** fibers were cured for 15 hours at 60 °C before the measurement to trigger the Diels-Alder reaction. Each fiber was tested 10 times.

Life Cycle Assessment

The comparative analysis of the CMF and HMF production was performed following the LCA methodology, which is standardized in the ISO 14040-14044 series by the International Organization of Standardization.[1,2] This comparison was based on 8 different cases

from the literature using various biomass raw materials (i.e., 6 for CMF and 2 for HMF). At this early stage, the study was based on the IPCC 2021 method, which contains the Global Warming Potential (GWP100) climate change factors of IPCC with a timeframe of 100 years.

The data was incorporated into the SimaPro LCA software (version 9.3) and the Ecoinvent database version 3.8 (allocation at the point of substitution (APOS) system model) was used as a background source for each synthesis used (details in Tables. S2-4). The foreground data was then evaluated. The functional unit was "kg of monomer", which was the basis for the comparison of CMF versus HMF. The system boundary was set up following a cradle-to-factory gate approach. The solvents of the synthesis were included. Organic solvents were assumed to be recycled infinitely. HCl was assumed to be recycled until fully consumed by the reaction (1 eq. HCl / carbohydrate). The electricity needed to maintain the reaction at a constant temperature was calculated from the enthalpy of the reaction (Table S1, Eq. 1-4), assuming perfect insulation. The energy required to heat the solvents from 25 °C to the reaction temperature was calculated from the specific heat capacities (Table S1, Eq. 5). Since lab-scale synthesis generally does not optimize the volume of solvents, only the energy required to maintain constant reaction temperature was considered as the base case. The total energy consumption was also used to calculate the GHG emissions (Tables S6 and S7).

Electricity required for other parts of the synthesis (e.g., pumping) was not included. Considering the mass balance for LCA, a compost treatment was added for handling the waste from each process. The distance from the production location to the compost treatment location was assumed to be 20 km. Transportation of the feedstock and solvents from one factory to the other for the CMF/HMF production was excluded. At lab scale, CMF can be produced from many biomass substrates in high yield. In this work, for the GHG emissions calculation of CMF production, 6 cases from literature using various biomass raw materials were studied and selected, which used (A) corn stover,[3] (B) glucose,[3] (C) sucrose,[3] (D) sucrose, [4] (E) fructose,[4] and (F) fructose.[5] The data for LCA on corn stover (case A) was obtained from literature, where the worst-case scenario was chosen (0.15 kg CO_2-eq/kg Corn stover dry matter).[6] The data on glucose (case B) from corn cultivation was based on the worst case scenario in the paper (1.1 kg CO_2-eq/kg glucose dry solid[7]), which matches the corresponding one in the Ecoinvent database. The LCA data on fructose (0.94 kg CO_2 eq/kg fructose, case E) and sucrose (0.71 kg CO_2-eq/kg sucrose) from sugar beet (cases C and D) were based on Garcia Gonzalez's work.[8,9] For the GHG emissions calculation of HMF production, 2 cases from the literature were studied, both of which used fructose as the raw biomass material for HMF production. They differ primarily in solvent systems. One case relied on the use of a biphasic water/DMC solvent system (case G),[10] while the other used a monophasic DMSO solvent system (case H).[11] The LCA on DMC was obtained from the literature (0.39 kg-CO_2-eq per kg-DMC).[8]

In the LCA model, the Swedish electricity production mix was assumed to be used as an energy source in the base case. A sensitivity analysis was performed by comparing it with an EU electricity production mix, which has a slightly higher proportion of fossil-fuel-based electricity. This analysis was based only on the energy required to maintain constant reaction temperature (the base case).

Synthesis of bio-based HMF

Bio-based HMF was produced, recovered, and purified following our established procedures.[10] HMF was produced by the continuous dehydration of 30% fructose in a water–DMC biphasic system (volume ratio 1:3, respectively). The reaction was carried out continuously at 180 °C by feeding 9 mL/min of the reaction mixture using an HPLC pump (515 HPLC pump, Waters, Massachusetts, USA). The outcome solution from the reaction was cooled down in an ice bath during the whole experiment. From the produced solution, the organic phase was separated from the aqueous phase and then used for the recovery of HMF. The recovery steps of HMF were started by removing the dark color using 5% (w/v) activated carbon (AC), followed by neutralizing the pH using 2% (w/v) sodium carbonate (Na_2CO_3) before concentrating *in vacuo*. The purity of the obtained HMF was confirmed by ^{1}H NMR spectroscopy (ESI, Fig. S1).

Synthesis of CMF

HMF (3.35 g, 23.2 mmol) was ground into a fine powder and dissolved in dichloromethane (DCM, 35 mL) in a 100 mL round bottom flask. HCl (15 mL, 37%) was added and the reaction mixture was stirred overnight at room temperature. Next, the reaction mixture was extracted with DCM (3 × 35 mL). The combined organic phase was mixed with silica gel (1.17 g) and Na_2SO_4 (3.52 g, 24.8 mmol) to remove the black humins and residual water, respectively. The solution was vacuum filtered through a membrane filter filled with Celite (~10 g). The filtrate was concentrated *in vacuo* to give CMF as a black viscous liquid (2.90 g, 76%). The product was stored below 5 °C to prevent decomposition.^{1}H NMR (DMSO-d_6, 400 MHz): δ ppm 9.60 (s, 1H, O=CH), 7.51 (d, 1H, J=3.56 Hz, Fur-H), 6.83 (d, 1H, J=3.56 Hz, Fur-H), 4.92 (s, 2H, Fur-CH_2). ^{13}C NMR (DMSO-d_6, 101 MHz): δ ppm 178.5, 156.0, 152.5, 123.9, 112.7, 36.8.

Synthesis of 2a

To a 500 mL round bottom flask was added CMF (9.51 g, 65.8 mmol), 4-hydroxybenzaldehyde (8.06 g, 66.0 mmol) and DMSO (150 mL) with stirring at room temperature under nitrogen. When all reagents were fully dissolved, a powder of K_2CO_3 (9.12 g, 66.0 mmol) was added. The mixture was stirred overnight at room temperature. Subsequently, the black reaction mixture was diluted with water (300 mL) and extracted with DCM (3 × 300 mL). The combined organic phase was washed with distilled water (4 × 200 mL), dried over Na_2SO_4, suction filtered and concentrated *in vacuo*. The resulting solid was dried under vacuum at 50 °C overnight, yielding **2a** as a

brown solid (13.8 g, 91%, mp: 76 °C, DSC). ^{1}H NMR (DMSO-d_6, 400 MHz): δ ppm 9.90 (s, 1H), 9.62 (s, 1H), 7.90 (d, 2H, *J*=2.12, 2.76, 8.61 Hz), 7.55 (d, 1H, *J*=3.49 Hz), 7.26 (d, 2H, *J*=2.00, 2.76, 8.61 Hz), 6.93 (d, 1H, *J*=3.48 Hz), 5.36 (s, 2H). ^{13}C NMR (DMSO-d_6, 400 MHz): δ ppm 92.18, 179.01, 162.93, 155.90, 153.02, 132.26, 130.06, 124.20, 115.68, 113.64. HR-MS (ESI+, m/z): [M+H$^+$] calculated for $C_{13}H_{11}O_4^+$: 231.0657 found 231.0650.

Synthesis of 2b

To a 2 L round bottom flask was added CMF (62.0 g, 0.430 mol), vanillin (65.4 g, 0.430 mol) and DMSO (800 mL) at room temperature with stirring. When the reagents were fully dissolved, K_2CO_3 (59.4 g, 0.430 mol) was added. The reaction mixture was stirred overnight at room temperature. Next, the black reaction mixture was poured into distilled water (8 L) and the formed precipitate was filtered, washed with distilled water, and dried under vacuum at 50 °C overnight, yielding a light brown powder as **2b** (101.1 g, 90%, mp: 137.8 °C, DSC). ^{1}H NMR (DMSO-d_6, 400 MHz): δ ppm 9.87 (s, 1H), 9.62 (s, 1H), 7.58 (dd, 1H, *J*=8.02, 1.65 Hz), 7.56 (d, 1H, *J*=3.49 Hz) 7.44 (d, 1H, *J*=1.94 Hz), 7.36 (d, 1H, *J* = 8.2 Hz), 6.92 (d, 1H, *J* = 3.52 Hz), 5.33 (s, 2H), 3.84 (s, 3H). ^{13}C NMR (DMSO-d_6, 101 MHz): δ ppm 191.90, 179.05, 155.87, 153.05, 152.74, 149.78, 130.79, 126.06, 124.21, 113.86, 113.23, 110.40, 62.68, 56.02. HR-MS (ESI+, m/z): [M+H$^+$] calculated for $C_{14}H_{13}O_5^+$ Calculated mass: 261.0763 found 261.0765.

Synthesis of 2c

To a 250 mL round bottom flask was added CMF (10.0 g, 69.2 mmol), syringaldehyde (12.6 g, 69.2 mmol) and DMSO (180 mL) with stirring at room temperature. When all reagents were fully dissolved, K_2CO_3 powder (9.56 g, 69.2 mmol) was added. The reaction mixture was stirred overnight at room temperature. In the next step, the black reaction mixture was poured into distilled water (2 L) and the precipitate was filtered, washed with distilled water, and dried under vacuum at 50 °C overnight, yielding **2c** as a light brown powder (17.9 g, 89%, mp: 90.4 °C, DSC). ^{1}H NMR (DMSO-d_6, 400 MHz): δ ppm 9.90 (s, 1H), 9.60 (s, 1H), 7.48 (d, 1H, *J*=3.57 Hz), 7.26 (s, 2H), 6.71 (d, 1H, *J*=3.55 Hz), 5.10 (s, 2H), 3.83 (s, 6H) ^{13}C NMR (DMSO-d_6, 101 MHz): δ ppm 192.38, 178.93, 157.21, 153.95, 152.81, 132.69, 113.23, 107.10, 66.10, 56.61 HR-MS (ESI+, m/z): [M+H$^+$] calculated for $C_{15}H_{15}O^+$: 291.0869 found 291.0865.

Synthesis of 3a

To a 25 mL round bottom flask was added **2a** (82.0 mg, 0.356 mmol) and acetonitrile (4 mL) with stirring at 0 °C until fully dissolved. $NaBH_4$ (20.4 mg, 0.539 mmol) was then added in small portions over 10 min with stirring under nitrogen. The reaction was carried out for 2 h at 20 °C before saturated NH_4Cl solution (2 mL) was added. After evaporation of the acetonitrile, additional water (2 mL) was added. The resulting solution was then extracted with DCM (3 × 4 mL). The combined organic phase was dried with Na_2SO_4 (880 mg), filtered and concentrated *in vacuo* to give **3a** as a pale yellow solid (63.3 mg, 76%, mp: 97.3 °C, DSC). ^{1}H NMR (THF-d_8, 400 MHz): δ 7.25 (d, 2H, *J* = 8.71 Hz, Ar-H), 6.93 (d, 2H, *J* = 8.71 Hz, Ar-H), 6.37 (d, 1H, *J* = 3.11 Hz, Fur-*H*), 6.21 (d, 1H, *J* = 3.11 Hz, Fur-*H*) 4.97 (s, 2H, Fur-C*H*$_2$), 4.50 (d, 2H, *J* = 3.00 Hz, ArC*H*$_2$), 4.46(d, 2H, *J* = 3.84 Hz, FurC*H*$_2$), 4.23 (t, 1H, *J* = 5.06 Hz, FurCH$_2$O*H*), 3.98 (t, 1H, *J* = 4.87 Hz, ArCH$_2$O*H*). ^{13}C NMR (THF-d_8, 101 MHz): δ ppm 158.61, 157.62, 150.97, 136.26, 128.45, 115.11, 111.05, 107.79, 64.50, 62.99, 57.58 HR-MS (ESI+, m/z): [M+Na$^+$] calculated for $C_{13}H_{14}O_4Na^+$: 257.0788 found 257.0790.

Synthesis of diol 3b

In a 25 mL round-bottom flask equipped with a magnetic stirrer, **2b** (100 mg, 0.386 mmol) was dissolved in methanol (5 mL) and cooled to 0 °C. Next, NaOMe (1.6 mg, 0.030 mmol) was added, followed by the addition of $NaBH_4$ (30.8 mg, 0.814 mmol) under N_2. The reaction was stirred at 20 °C for 3 h before a saturated NH_4Cl solution (2.5 mL) was added under stirring. After 10 min, methanol in the reaction mixture was evaporated, followed by the addition of water (2.5 mL). The resulting aqueous solution was then extracted with DCM (3 × 2.5 mL), and the combined organic phase was dried over Na_2SO_4 (880 mg) and concentrated *in vacuo* to give a yellow oil as the crude product. Crystallization of the crude oily product was induced by scratching the bottom of the round-bottom flask with a spatula, yielding **3b** as light yellow crystals (78 mg, 77%).

Synthesis of 3c

To a solution of **2c** (92.7 mg, 0.319 mmol) in methanol (5 mL) under nitrogen at 0 °C was added NaOMe (1.5 mg, 0.028 mmol), followed by the addition of small portions of $NaBH_4$ (19.8 mg, 0.523 mmol) during 15 min. The reaction was stirred for 2 h at 20 °C. Next, saturated NH_4Cl (2.5 mL) was added and stirred for another 10 min. Afterwards, methanol in the mixture was evaporated, and additional water (2.5 mL) was added. The resulting solution was then extracted with DCM (3 × 2.5 mL), and the combined organic phase was dried over Na_2SO_4 (880 mg) and concentrated *in vacuo* to give a yellow oil as the crude product. Crystallization of the crude oily product was induced by scratching the bottom of the round bottom flask with a spatula, yielding pure **3c** as light-yellow crystals (79.3 mg, 85%, mp: 85.2 °C, DSC). ^{1}H NMR (THF-d_8, 400 MHz): δ 6.62 (s, 2H, Ar-H), 6.19 (d, 1H, *J*=3.08 Hz, Fur-H), 6.13 (d, 1H, *J*=3.08 Hz, Fur-H), 4.83 (s, 1H, Fur-CH$_2$), 4.51 (d, 2H, *J*=5.90 Hz, Ar-CH$_2$), 4.45 (d, 2H, *J* = 6.13 Hz, Fur-CH$_2$), 4.24 (t, 1H, *J*=6.37 Hz, Fur-CH$_2$-OH), 4.11 (d, 1H, *J*=5.95 Hz, Ar-CH$_2$-OH), 3.78 (s, 6H, OMe). ^{13}C NMR (THF-d_8, 101 MHz): δ ppm 157.07, 154.70, 152.39, 139.56, 136.51, 110.61, 107.62, 104.36, 64.95, 57.63, 56.33. HR-MS (ESI+, m/z): [M+Na$^+$] calculated for $C_{15}H_{18}O_6Na^+$: 317.1001 found 317.0999.

Polyurethane synthesis

Homopolymers (**PU-3b** and **PU-C**) and copolymers (**PU-3b$_x$C$_y$**) were synthesized similarly. The synthetic procedure of non-crosslinked **PU-3b$_{67}$C$_{33}$** is described below as a typical example. To a 50 mL round-bottom flask with MDI (2.65 g, 10.6 mmol), **3b** (2.00 g, 7.57 mol), and PCL (M_n = 400 Da, 1.00 g, 2.50 mmol) was added 2-butanone (5.5 mL) dropwise with stirring at room temperature. During the addition of 2-butanone, the reaction temperature was kept below 30 °C. An additional portion of 2-butanone (2 mL) was added to flush down the residue on the reaction flask. Dibutyltin dilaurate (0.1 mL, 10 v%) was added dropwise, and the temperature was then increased to 40 °C. The viscosity immediately started to increase rapidly, and more 2-butanone (12 mL) was added to prevent coagulation. The reaction was then stirred for 1 h, until FTIR-analysis confirmed the depletion of isocyanate groups. The reaction was then quenched by DBA (1 mL, 2 M in 2-butanone). Next, the viscous reaction mixture in the round-bottom flask was rinsed and dissolved in 2-butanone (10 mL) and THF (10 mL). The resulting solution was then diluted with THF (40 mL) and precipitated into 400 mL TBME. The polymer was collected by filtration and dried overnight at 50 °C to yield a light-yellow solid as **PU-3b$_{67}$C$_{33}$** (5.01 g, 81%).

Reversible crosslinking by Diels-Alder reaction

Reversible crosslinking of **PU-3b$_{67}$C$_{33}$** is described as a typical example.
The obtained non-crosslinked **PU-3b$_{67}$C$_{33}$** (187 mg) was dissolved in THF (2 mL) at room temperature in a vial. When the polymer was completely dissolved, bis-maleimide (44.2 mg) was added, and the temperature was increased to 60 °C. After 20 h, gelation was observed, and another portion of THF (3 mL) was added to fully submerge the gel. After 24 h, the solvent was decanted and the remaining gel was collected and dried under vacuum at 116 °C (~30 °C above the T_g), yielding the crosslinked **PU-3b$_{67}$C$_{33}$g** as an orange solid (190 mg). The supernatant was dried separately to give a yellow solid (34.5 mg) for analysis.

Preparation of the solutions for wet spinning

Non-crosslinked PU-3b$_{33}$C$_{67}$

PU-3b$_{33}$C$_{67}$ (0.925 g), and DMF (5 mL), were added to a vial equipped with a magnetic stirrer. The vial was heated to 60 °C for 18 h to facilitate dissolution. The resulting spinning solution was then filtered through a 20 µm filter to remove any particles.

Non-crosslinked PU-3b$_{33}$C$_{67}$ with crosslinking agent

The non-crosslinked polymer **PU-3b$_{33}$C$_{67}$** (0.83 g), the crosslinking agent bis-maleimide (0.112 g), and DMF (5 mL) were added to a vial equipped with a magnetic stirrer. The vial was heated to 60 °C for 18 h to induce the crosslinking by Diels-Alder reaction, yielding an insoluble gel **PU-3b$_{67}$C$_{33}$g**. Afterwards, the obtained gel **PU-3b$_{67}$C$_{33}$g** was heated to a higher temperature at 140 °C for 5 minutes (to trigger the de-crosslinking by retro Diels-Alder reaction) until the gel was completely dissolved, yielding a mixture of non-crosslinked **PU-3b$_{67}$C$_{33}$g** and the crosslinking agent bis-maleimide in the solution. The resulting solution was then filtered through a 20 µm filter to remove any particles, and then it was directly subjected to wet-spinning.

Wet spinning

The spinning solution was loaded into a 10 mL syringe pump and extruded at 5 m/min (0.3 mL/min) through a spinneret (6 holes, D = 80 µm) into a 90 cm coagulation bath with deionized water. The fiber was collected by a take-up roller at take-up speeds of 5 m/min, and 6 m/min. After the spinning process, the fibers were air-dried at 60 °C for 12 hours. For the obtained fibre without crosslinking agent, the dried fibre was referred to as **PU-3b$_{67}$C$_{33}$** fibre. For the obtained fibre containing crosslinking agent, crosslinking by Diels-Alder reaction took place during the drying period, and thus the obtained fibre was referred to as **PU-3b$_{33}$C$_{67}$g** fibre. The draw ratio was calculated by dividing the take-up speed by the extrusion speed.

Fiber recycling

The crosslinked **PU-3b$_{33}$C$_{67}$g** fibre (433 mg) was cut into pieces and mixed with PET (96 mg) and cotton (62 mg) fibres. DMF (3 mL) was added into the heterogenous mixture. The mixture was heated in an oil bath to 140 °C for 7 min for de-crosslinking. Afterwards, the brown slurry was filtered through a 20 µm filter using centrifugation (4000 RPM, 2 min). The filtrate (containing de-crosslinked **PU-3b$_{33}$C$_{67}$** polymer and the free crosslinking agent) was directly used as spinning solution in the procedure above to give an originally non-crosslinked fibre, which was crosslinked after being dried at 60°C for 12 hours, yielding a crosslinked recycled fibre namely **PU-3b$_{33}$C$_{67}$g-R.**

Calculations

The enthalpy of the reaction was calculated from the enthalpies of formation at standard pressure and temperature (1 atm, 25 °C) using the following equations:

Eq. 1 $\Delta H_{rxn} = \Delta H^0_{rxn} + \Delta C_p \times \Delta T$

Eq. 2 $\Delta C_p = -C_p(products) + C_p(reactants)$

Eq. 3 $\Delta H^0_{rxn} = \sum \Delta H^0_f(products) - \sum \Delta H^0_f(reactants)$

where ΔH_{rxn} = enthalpy of the reaction at the reaction temperature, ΔH^0_{rxn} = enthalpy of the reaction at standard conditions, ΔT = difference between the reaction temperature and room temperature, ΔH^0_f= standard heat of formation, and C_p = molar heat capacity. Assuming full insulation of the reactor, the energy required to maintain a constant temperature can be calculated as:

Eq. 4 $q_{rxn} = \Delta H_{rxn} \times F$

where q_{rxn} = the energy required to maintain a constant temperature, and F = rate of production.
Furthermore, the energy required to heat the solvents and reagents to the reaction temperature can be calculated as:

Eq. 5 $q_{heating} = \sum C_p \times \Delta T$

where $q_{heating}$ is the energy required to heat the reagents to the reaction temperature.

Table S1. Physical properties used for the calculations.[12–22] Due to the lack of accurate data on the physical properties of corn stover or cellulose, glucose was used instead.

	$\Delta H°_f$ kJ/mol	Cp J/g*K
Glucose	-1267.8	1.22
Fructose	-1266	1.12
Sucrose	-2226.1	1.26
HCl (36 %)	-167.2	2.46
HCl (32 %)	-167.2	2.55
Water	-285.8	4.1667
CMF	-227.1	0.92
HMF	-437	1.2
DMSO	-	1.96
PhCl	-	1.33
toluene	-	1.70
DCE	-	1.29
DCM	-	1.20
DMC	-	1.88

Results and Discussion

Table S2. Reaction components for each synthesis protocol of CMF (A-F) and HMF (G-H).

Case	Starting material	Product	Aq phase	Org phase	Temperature (°C)
**(A)**	Corn Stover	CMF	HCl (36%)	DCE	100
**(B)**	Glucose	CMF	HCl (36%)	DCE	100
**(C)**	Sucrose	CMF	HCl (32%)	DCE	130
**(D)**	Sucrose	CMF	HCl (36 %)	DCE	100
**(E)**	Fructose	CMF	HCl (36%)	PhCl	75
**(F)**	Fructose	CMF	HCl (32%)	DCM	100
**(G)**	Fructose	HMF	H_2O	DMC	180
**(H)**	Fructose	HMF	-	DMSO	130

Table S3. Inventory analysis for each synthesis of CMF (A-F) and HMF (G-H) – lab data from literature.

Case	Starting material	Starting material mass (g)	Product	Product mass (g)	Energy[a] [Base Case] (kJ/g sugar)	Energy[b] [Total] (kJ/g sugar)	Aq. Solvent (mL)	Org. Solvent (mL)
**(A)**	Corn Stover	0.77	CMF	0.21	0.37	42.4	70	140
**(B)**	Glucose	0.70	CMF	0.46	0.37	46.3	70	140
**(C)**	Sucrose	10.0	CMF	5.13	0.33	5.26	100	100
**(D)**	Sucrose	0.71	CMF	0.55	0.31	45.6	70	140
**(E)**	Fructose	18.0	CMF	13.7	0.36	2.68	25	500
**(F)**	Fructose	10.0	CMF	6.32	0.36	3.86	100	100
**(G)**	Fructose	320	HMF	166	0.11	5.27	1000	3000
**(H)**	Fructose	0.50	HMF	0.32	0.13	2.99	-	6

[a] The energy required for maintaining a stable reaction temperature, this considered the base case.

[b] Two parts of energy-use are considered: 1. the energy required for maintaining a stable reaction temperature, and 2. the energy required for heating solvents and reagents from 25 °C to the reaction temperature (the total energy consumption).

Table S4. Inventory analysis for each synthesis of CMF (A-F) and HMF (G-H) - Resources / Processes used into SimaPro software.

Resources[a] / Processes[b]	
**Energy**	Electricity, low voltage {SE}| market for[c] (base case) (Ecoinvent database).<br>Electricity, low voltage {Europe without Switzerland}| market group for[c] (sensitivity analysis) (Ecoinvent database).
**DMSO**	Dimethyl sulfoxide {RER}| production.
**HCl**	Hydrochloric acid, without water, in 30% solution state {RER}| Mannheim process[d].
**DCE**	Ethylene dichloride {RER}| production.
**Compost treatment**	Municipal waste collection service by 21 metric ton lorry {CH}| processing.<br>Compost {CH}| treatment of biowaste, industrial composting.
**PhCl**	Monochlorobenzene {RER}| benzene chlorination.
**DCM**	Dichloromethane {RER}| production.
**Water**	Tap water {Europe without Switzerland}| tap water production, conventional treatment.

[a] These processes include other processes (inputs). They can be found in Ecoinvent database v3.8.
[b] "Transformation processes" were chosen for all the chemicals (the process in the Ecoinvent database). All the inputs required to manufacture a product, and inputs from its associated emissions and resource extractions, are included. Transportation and related losses were not considered.
[c] "Market process for electricity, low voltage" (name of the process in the Ecoinvent database) was chosen for modelling, as this is recommended when using supply mixes on lab scale in LCA studies.[23]
[d] This process was chosen since this was the one used in the laboratory.
The starting materials sources for all the synthesis and the solvent dimethyl carbonate (DMC) data are not included in this table since they are taken from the literature (explained in the main paper).

Table S5. All contributions related to GHG emissions of the synthesis of CMF (A-F) and HMF (G-H) from various carbohydrate biomass sources as predicted by LCA, assuming all streams have the same temperature, which is equal to the reaction temperature (only energy required to maintain constant reaction temperature was considered, i.e. the base case).

Case	Starting material	Product	Starting material emissions (kg CO_2-eq/kg)	Aq. Solvent (kg CO_2-eq/kg)	Org. Solvent[a] (kg CO_2-eq/kg)	Waste collection service (kg CO_2-eq/kg)	Swedish energy (kg CO_2-eq/kg)	Compost treatment (kg CO_2-eq/kg)
**(A)**	Corn Stover	CMF	0.545	0.589	-	0.065	0.02	-0.21
**(B)**	Glucose	CMF	1.66	0.246	-	0.13	0.0083	-0.040
**(C)**	Sucrose	CMF	0.919	0.222	-	0.072	0.0059	-0.023
**(D)**	Sucrose	CMF	1.34	0.382	-	0.22	0.0092	-0.069
**(E)**	Fructose	CMF	1.49	0.303	-	0.14	0.0085	-0.045
**(F)**	Fructose	CMF	1.24	0.213	-	0.0077	0.0070	-0.024
**(G)**	Fructose	HMF	1.81	0.00152	-	0.023	0.0032	-0.072
**(H)**	Fructose	HMF	1.47	-	-	0.14	0.0030	-0.044

[a] No contribution for the production of organic solvents since they were assumed to be recycled infinitely.

SUPPORTING INFORMATION

Table S6. The total GHG emissions results of the synthesis of CMF (A-F) and HMF (G-H) from various carbohydrate biomass sources as predicted by LCA, assuming the solvents and reagents need to be heated to the reaction temperature and that no heat is reused in the process (the total energy consumption- Swedish electricity production). The largest and the energy contributions are also shown.

Case	Starting material	Product	Total GHG (kg CO_2-eq/kg)	Largest contribution (kg CO_2-eq/kg)	Energy contribution (Swedish electricity production-base case) (kg CO_2-eq/kg)
(A)	Corn Stover	CMF	3.26	2.26 (production of electricity)	2.26
(B)	Glucose	CMF	2.92	1.66 (production of glucose)	1.04
(C)	Sucrose	CMF	2.00	0.92 (production of sucrose)	0.877
(D)	Sucrose	CMF	1.82	1.34 (production of sucrose)	0.147
(E)	Fructose	CMF	1.85	1.49 (production of fructose)	0.0908
(F)	Fructose	CMF	1.48	1.24 (production of fructose)	0.0523
(G)	Fructose	HMF	1.92	1.81 (production of fructose)	0.151
(H)	Fructose	HMF	1.63	1.47 (production of fructose)	0.069

Table S7. All contributions related to GHG emissions of the synthesis of Table S6 (the total energy consumption - Swedish electricity production)

Case	Starting material	Product	Starting material emissions (kg CO_2-eq/kg)	Aq. Solvent (kg CO_2-eq/kg)	Org. Solvent[a] (kg CO_2-eq/kg)	Waste collection service (kg CO_2-eq/kg)	Swedish energy (kg CO_2-eq/kg)	Compost treatment (kg CO_2-eq/kg)
(A)	Corn Stover	CMF	0.545	0.589	-	0.065	2.26	-0.21
(B)	Glucose	CMF	1.66	0.246	-	0.13	1.04	-0.040
(C)	Sucrose	CMF	0.919	0.222	-	0.072	0.877	-0.023
(D)	Sucrose	CMF	1.34	0.382	-	0.22	0.147	-0.069
(E)	Fructose	CMF	1.49	0.303	-	0.14	0.0908	-0.045
(F)	Fructose	CMF	1.24	0.213	-	0.0077	0.0523	-0.024
(G)	Fructose	HMF	1.81	0.00152	-	0.023	0.151	-0.072
(H)	Fructose	HMF	1.47	-	-	0.14	0.0694	-0.044

[a] No contribution for organic solvents since they were assumed to be recycled an infinite number of times.

Table S8. Sensitivity analysis comparing Swedish to EU electricity production of the synthesis of CMF (A-F) and HMF (G-H)

Case	Starting material	Product	Total GHG (Sensitivity analysis) (kg CO_2-eq/kg)	Total GHG (base case) (kg CO_2-eq/kg)	Energy contribution (EU electricity production) (kg CO_2-eq/kg)	Energy contribution (Swedish electricity production-base case) (kg CO_2-eq/kg)
(A)	Corn Stover	CMF	1.14	1.01	0.146	0.0200
(B)	Glucose	CMF	1.94	1.89	0.061	0.0083
(C)	Sucrose	CMF	1.17	1.13	0.044	0.0059
(D)	Sucrose	CMF	1.74	1.68	0.068	0.0092
(E)	Fructose	CMF	1.82	1.77	0.062	0.0085
(F)	Fructose	CMF	1.48	1.44	0.052	0.0070
(G)	Fructose	HMF	1.79	1.77	0.023	0.0031
(H)	Fructose	HMF	1.59	1.57	0.022	0.0030

Table S9. Sensitivity analysis using the EU electricity supply mix for comparison. All contributions related to GHG emissions of the synthesis of CMF (A-F) and HMF (G-H) from various carbohydrate biomass sources as predicted by LCA (considering only the energy required to maintain constant reaction temperature - base case).

Case	Starting material	Product	Startin material emissions (kg CO_2-eq/kg)	Aq. Solvent (kg CO_2-eq/kg)	Org. Solvent[a] (kg CO_2-eq/kg)	Waste collection service (kg CO_2-eq/kg)	Energy (EU electricity production) (kg CO_2-eq/kg)	Compost treatment (kg CO_2-eq/kg)
(A)	Corn Stover	CMF	0.545	0.589	-	0.065	0.15	-0.21
(B)	Glucose	CMF	1.66	0.246	-	0.13	0.061	-0.040
(C)	Sucrose	CMF	0.919	0.222	-	0.072	0.044	-0.023
(D)	Sucrose	CMF	1.34	0.382	-	0.22	0.068	-0.069
(E)	Fructose	CMF	1.49	0.303	-	0.14	0.062	-0.045
(F)	Fructose	CMF	1.24	0.213	-	0.0077	0.052	-0.024
(G)	Fructose	HMF	1.81	0.00152	-	0.023	0.023	-0.072
(H)	Fructose	HMF	1.47	-	-	0.14	0.022	-0.044

[a] The contribution from the production of organic solvents was excluded since they were assumed to be recycled an infinite number of times.

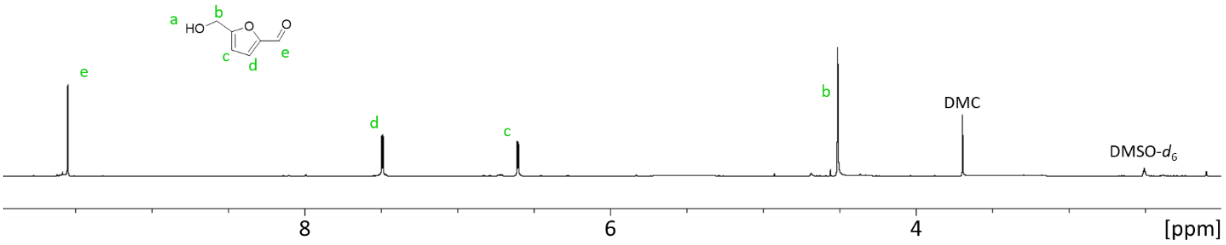

Figure S1. ^{1}H-NMR spectrum of the synthesized bio-based HMF from fructose.

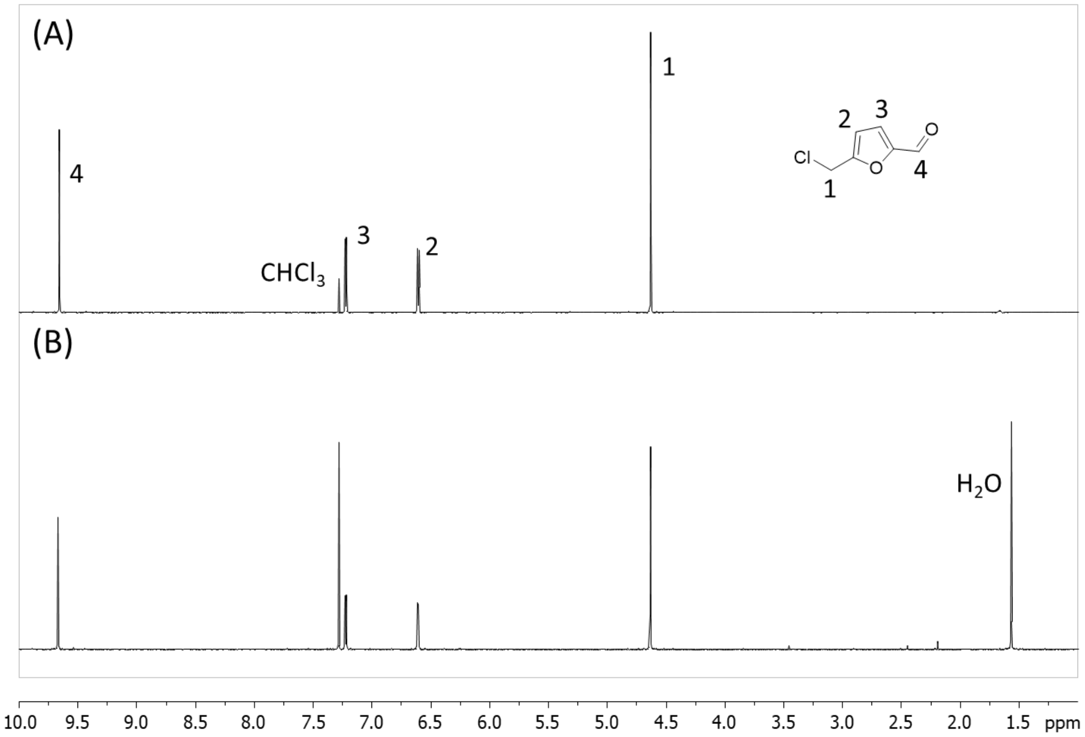

Figure S2. ^{1}H-NMR spectra of fossil-based CMF (A) and bio-based CMF(B).

Table S10. Conversion of the reaction between vanillin and CMF in different solvents, as determined by the peak integrals of the ^{1}H-NMR spectra.

Solvent	Conversion
DMSO	100 %
DMF	86 %
MeOH	37 %
Acetone	12 %
ACN	10 %
H_2O	9 %
DCM	2 %
EtOAc	2 %
THF	0 %

Table S11. Yields of dialdehyde **2b** using bio-based and fossil-based vanillin and CMF.

	Bio-based Vanillin	Fossil-based Vanillin
Bio-based CMF	62 %	63 %
Fossil-based CMF	61 %	62 %

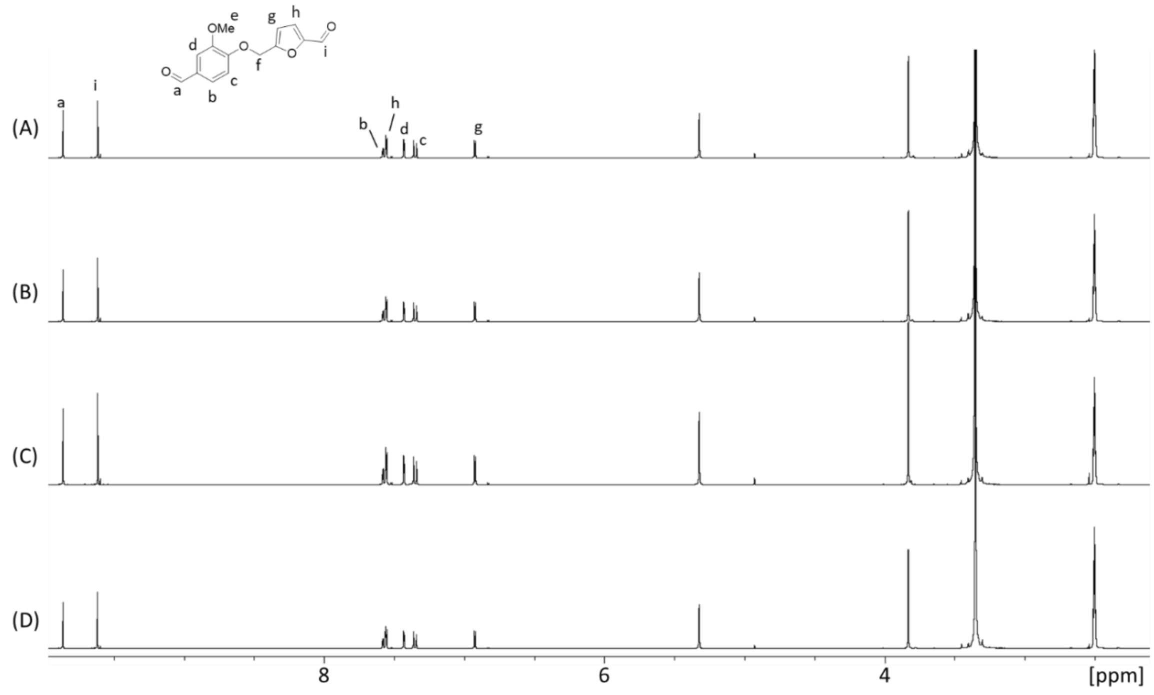

Figure S3. ^{1}H NMR spectra of (A) crude **2b** synthesized using bio-based CMF and fossil-based vanillin, (B) crude **2b** synthesized using bio-based CMF and bio-based vanillin, (C) crude **2b** synthesized using fossil-based CMF and bio-based vanillin, and (D) crude **2b** synthesized using fossil-based CMF and fossil-based vanillin.

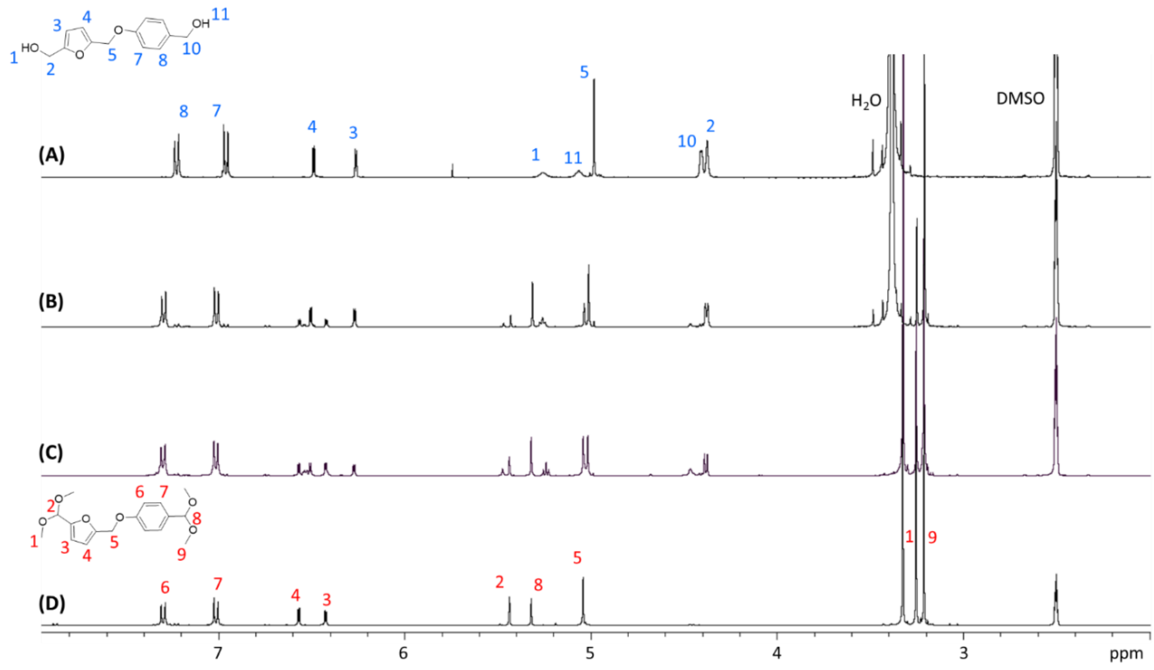

Figure S4. ^{1}H NMR spectra of (A) **3b** after reduction of **2b** with $NaBH_4$ in acetonitrile, (B) crude **3b** after reduction of **2b** with $NaBH_4$ in methanol without NaOMe, (C) crude **3b** after reduction of **2b** with $NaBH_4$ in methanol with 5 mol% NaOMe, and (D) crude **3b** after acetalation of **2b** with $NaBH_4$ in methanol with 10 mol% *p*-TsOH.

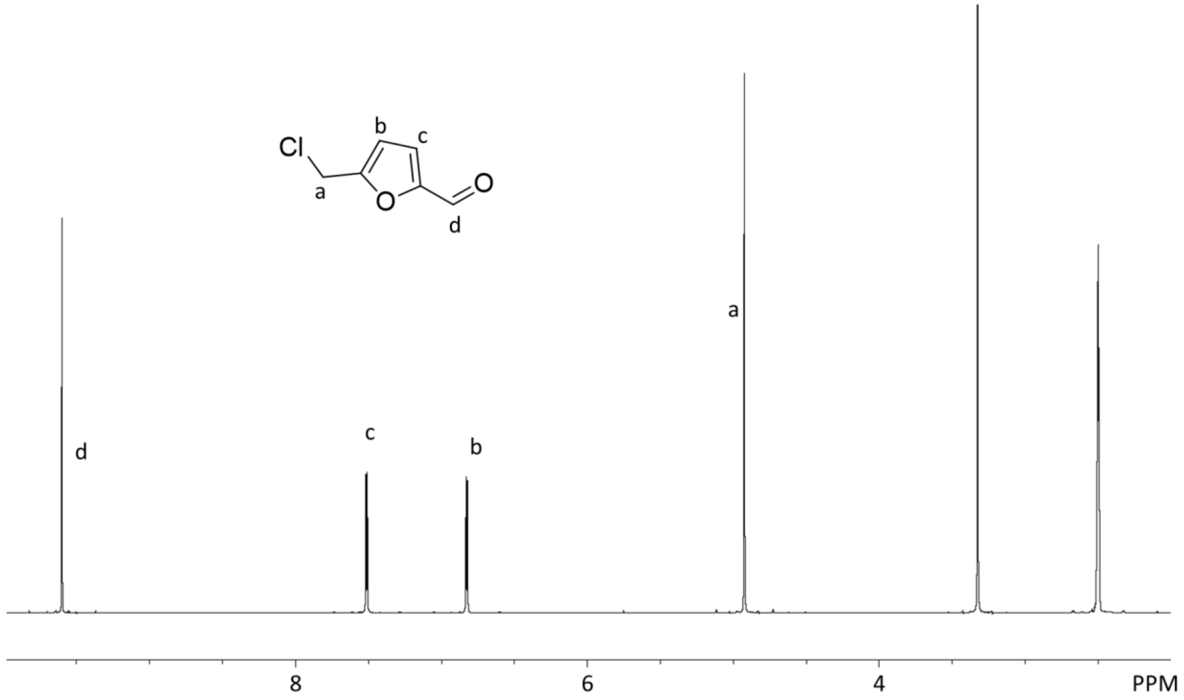

Figure S5. ^{1}H-NMR spectrum of CMF.

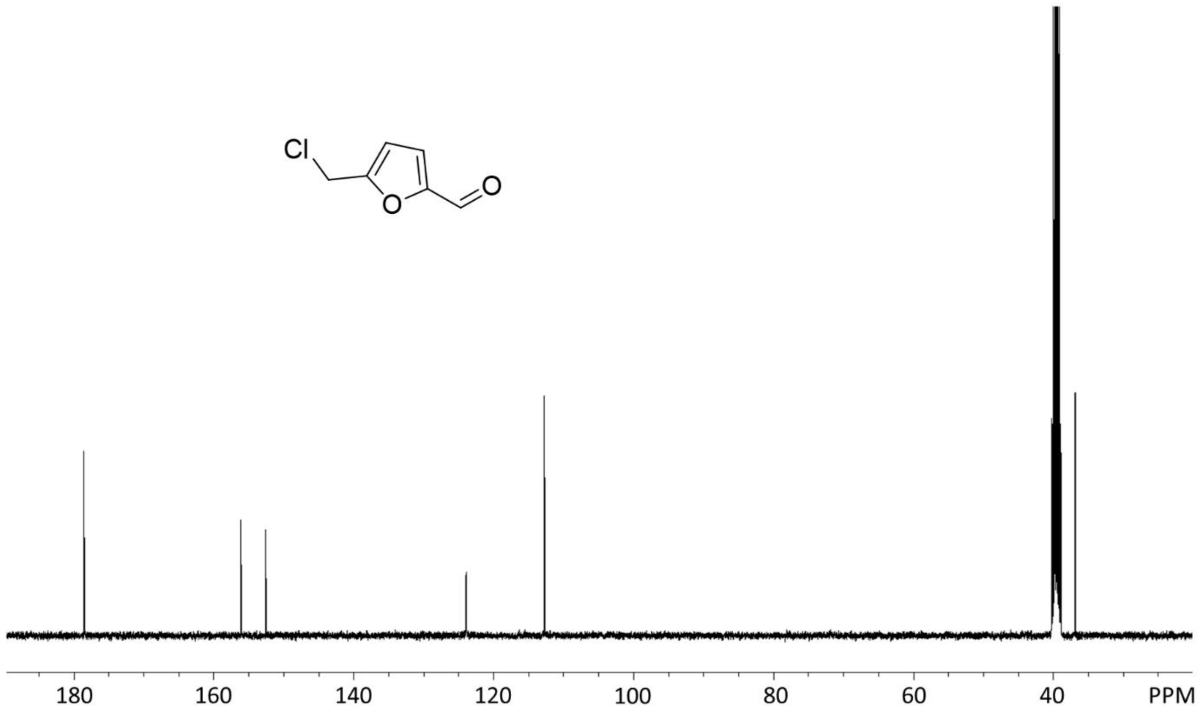

Figure S6. ^{13}C-NMR spectrum of CMF.

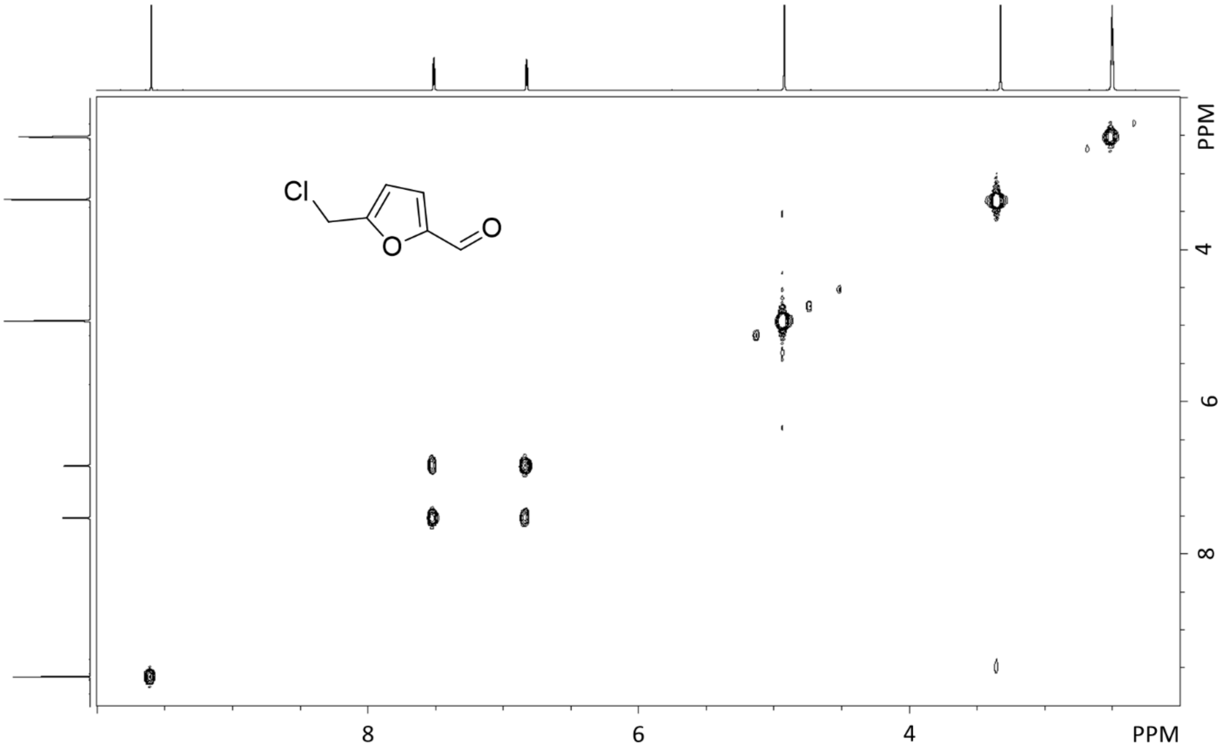

Figure S7. COSY spectrum of CMF.

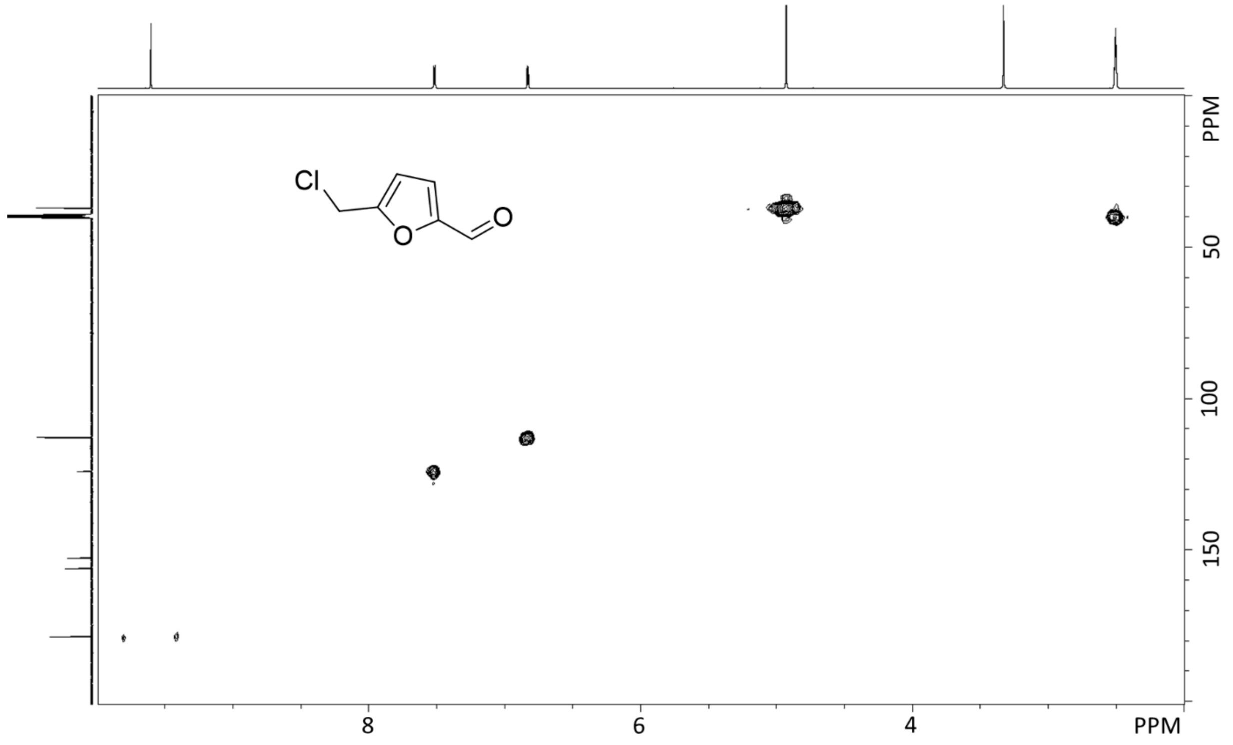

Figure S8. HMQC spectrum of CMF.

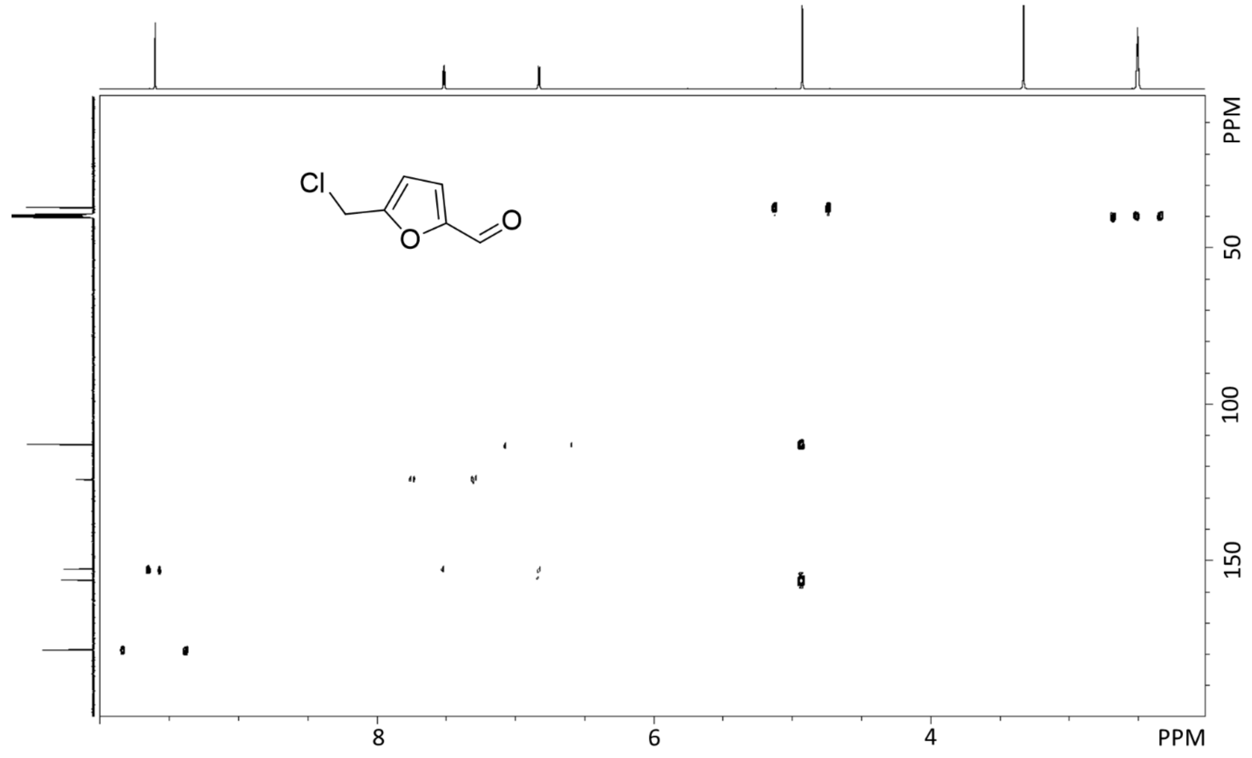

Figure S9. HMBC spectrum of CMF.

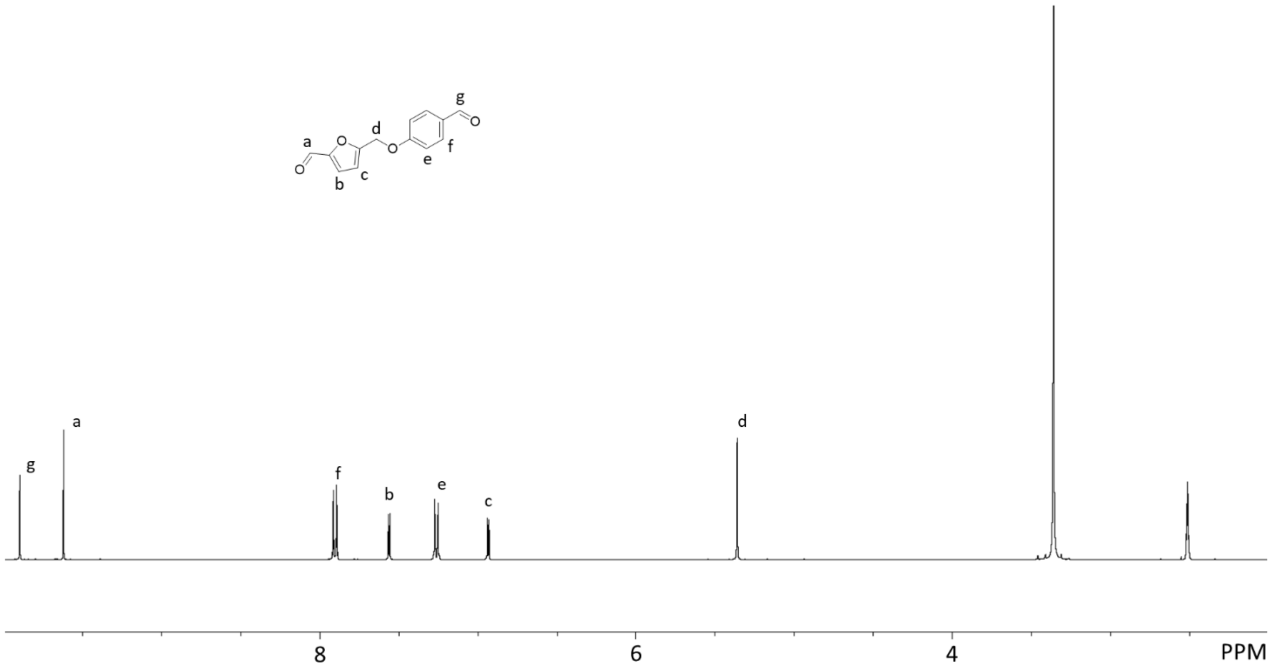

Figure S10. ^{1}H-NMR spectrum of **2a**.

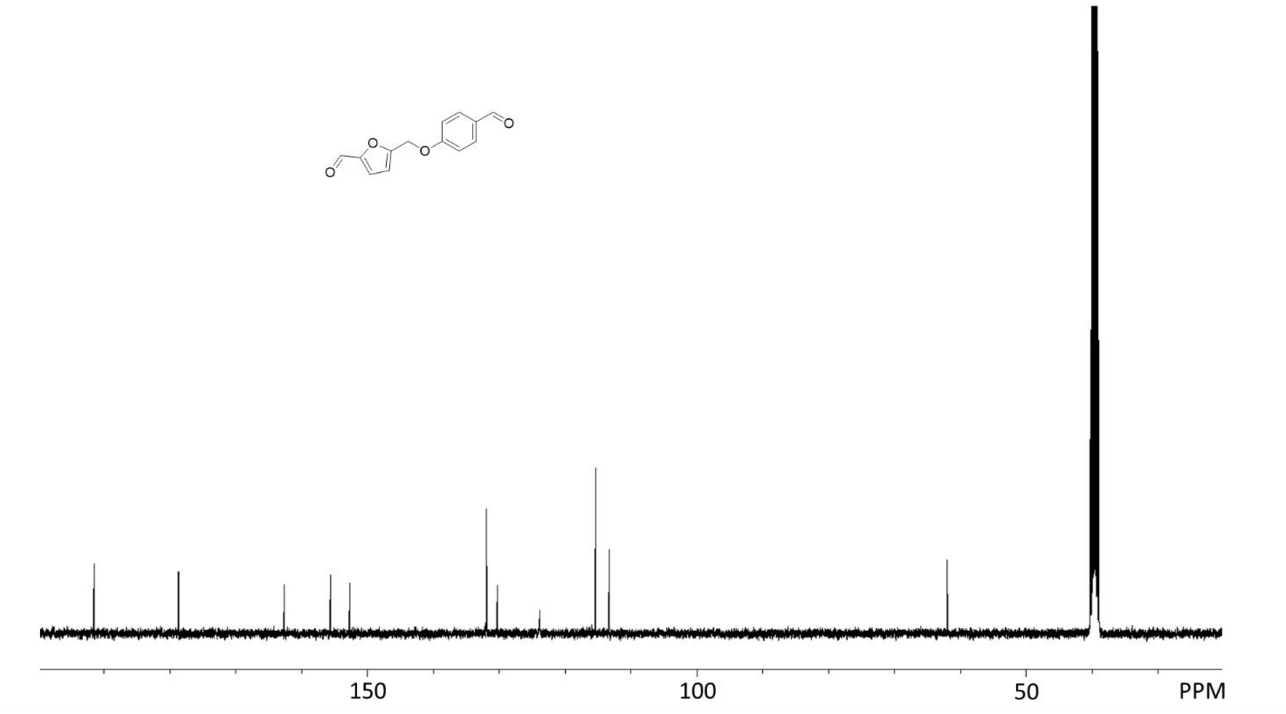

Figure S11. ^{13}C-NMR spectrum of **2a**.

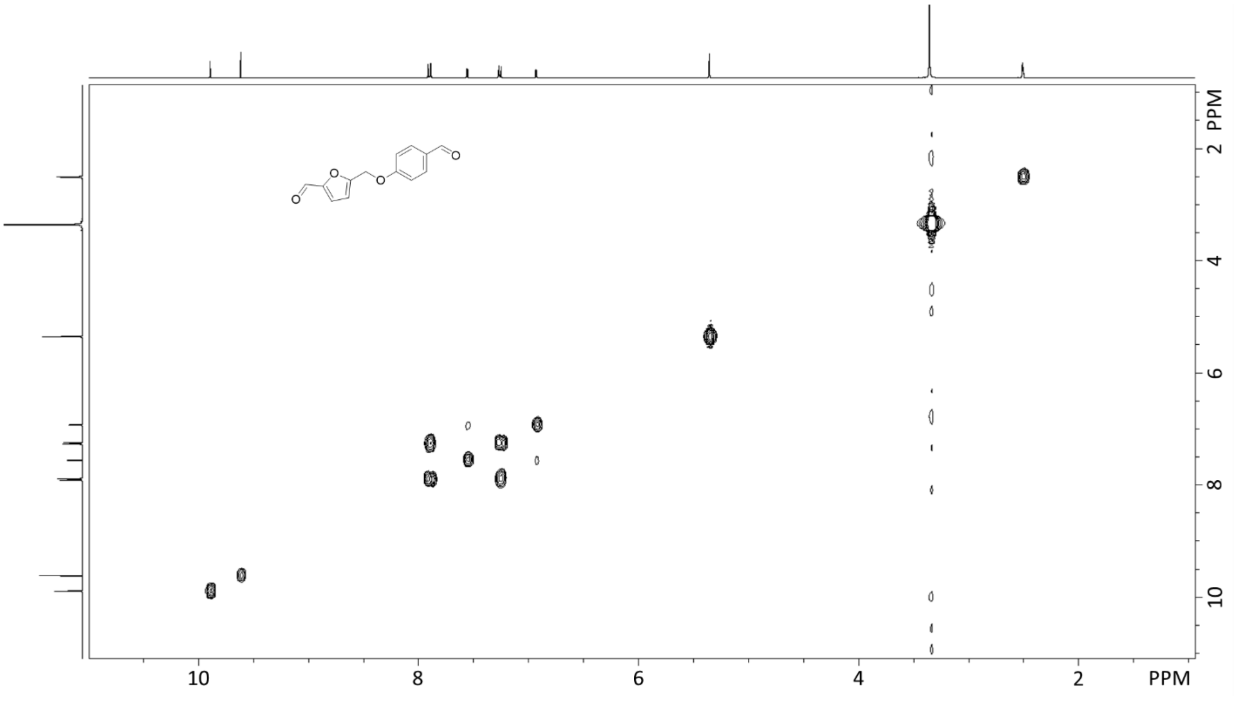

Figure S12. COSY spectrum of **2a**.

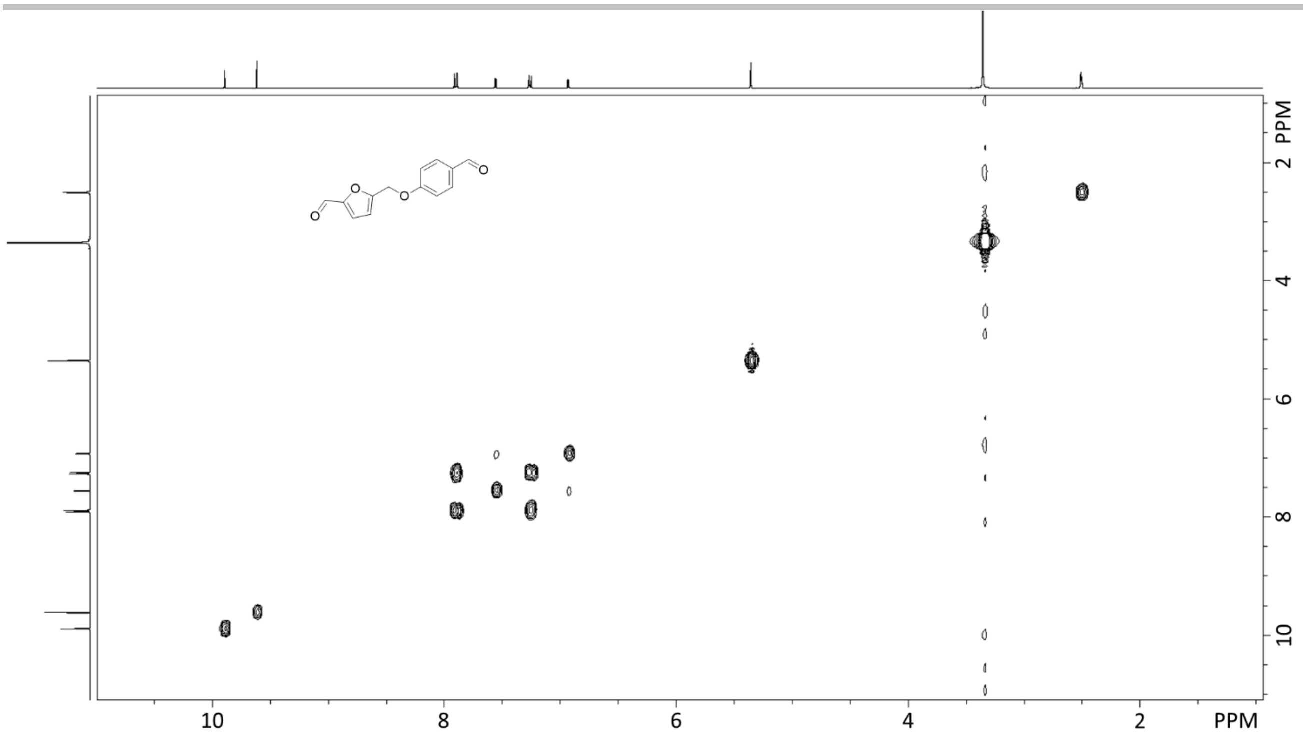

Figure S13. HMQC spectrum of **2a**.

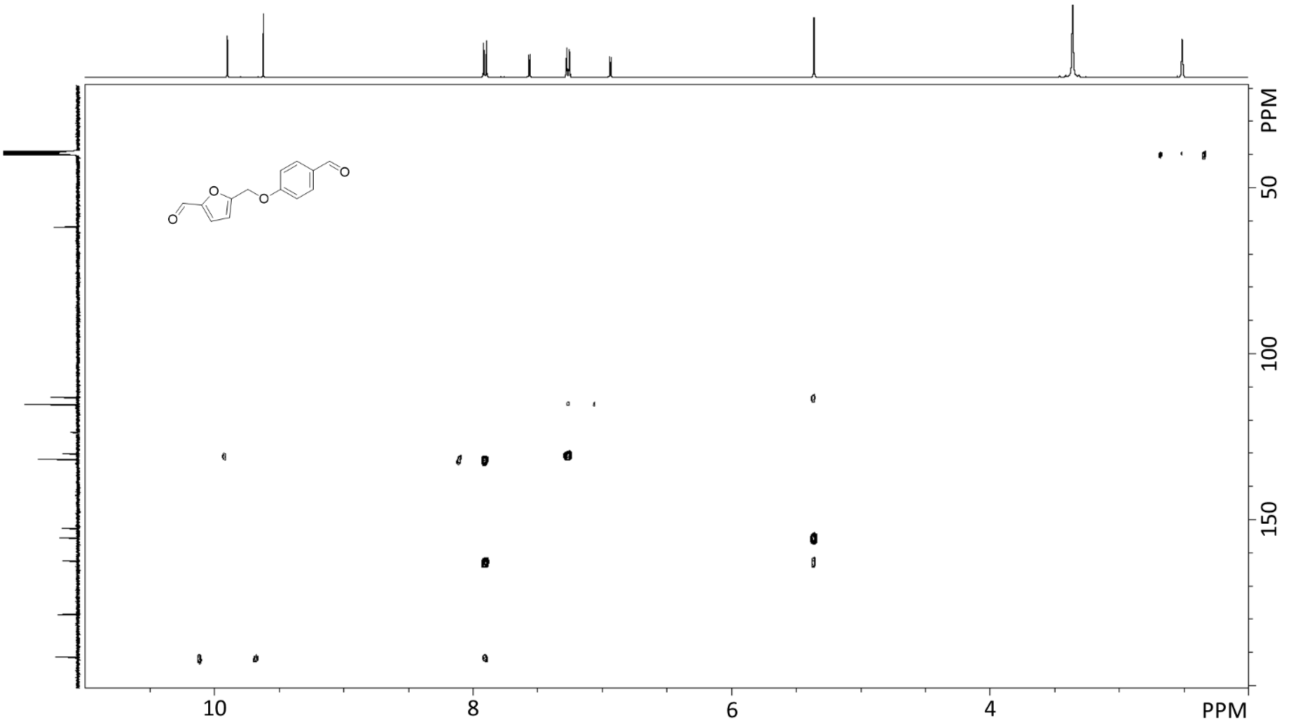

Figure S14. HMBC spectrum of **2a**.

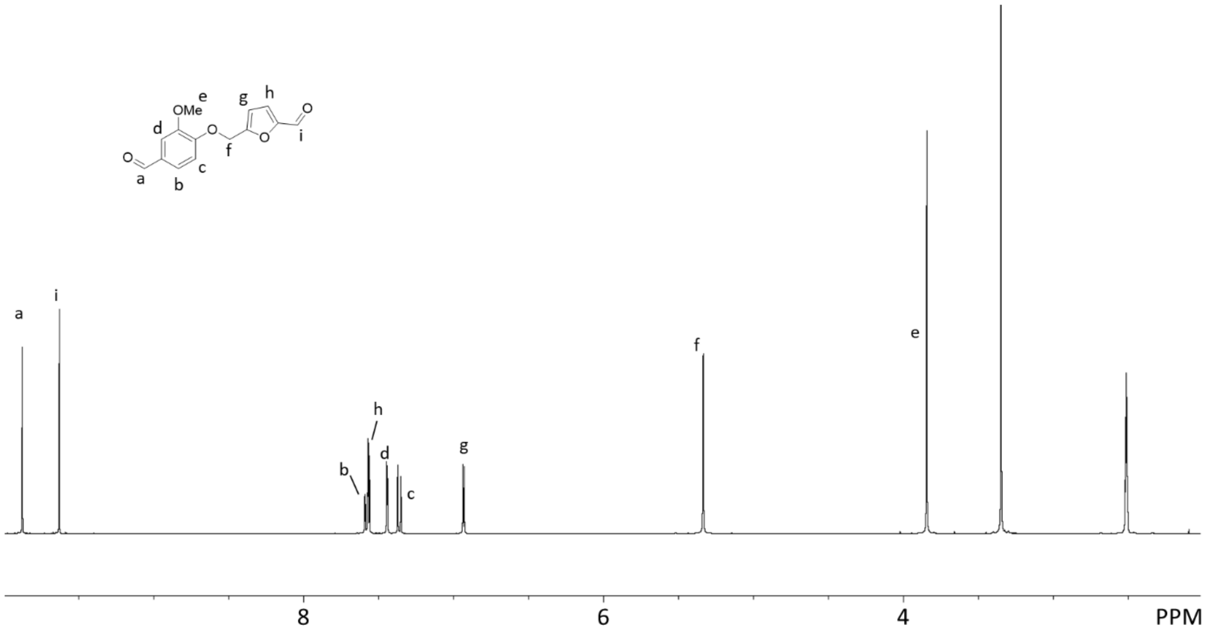

Figure S15. ^{1}H-NMR spectrum of **2b**.

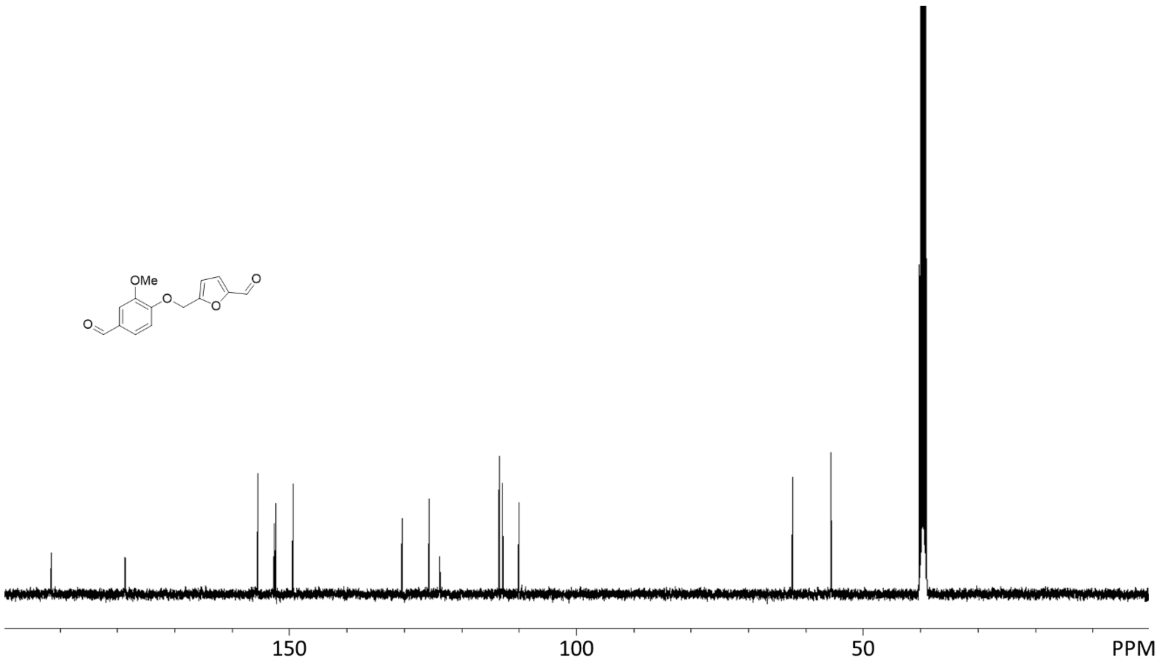

Figure S16. ^{13}C-NMR spectrum of **2b**.

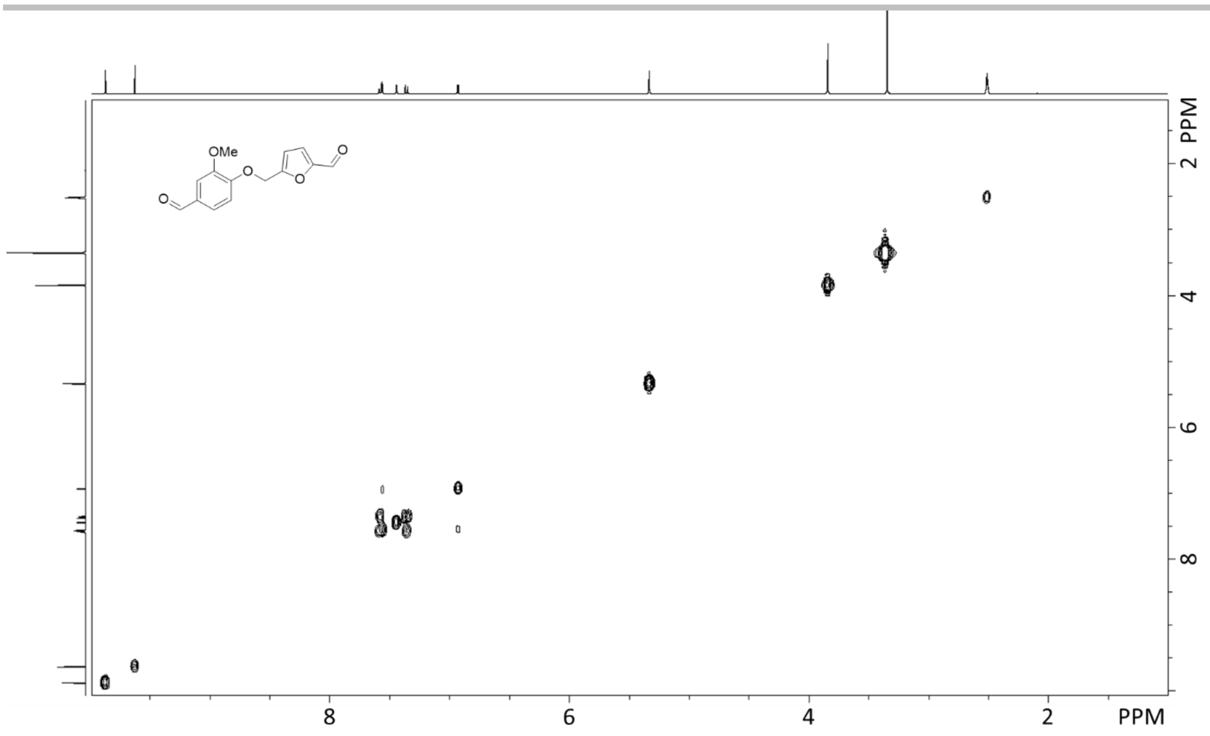

Figure S17. COSY spectrum of **2b**.

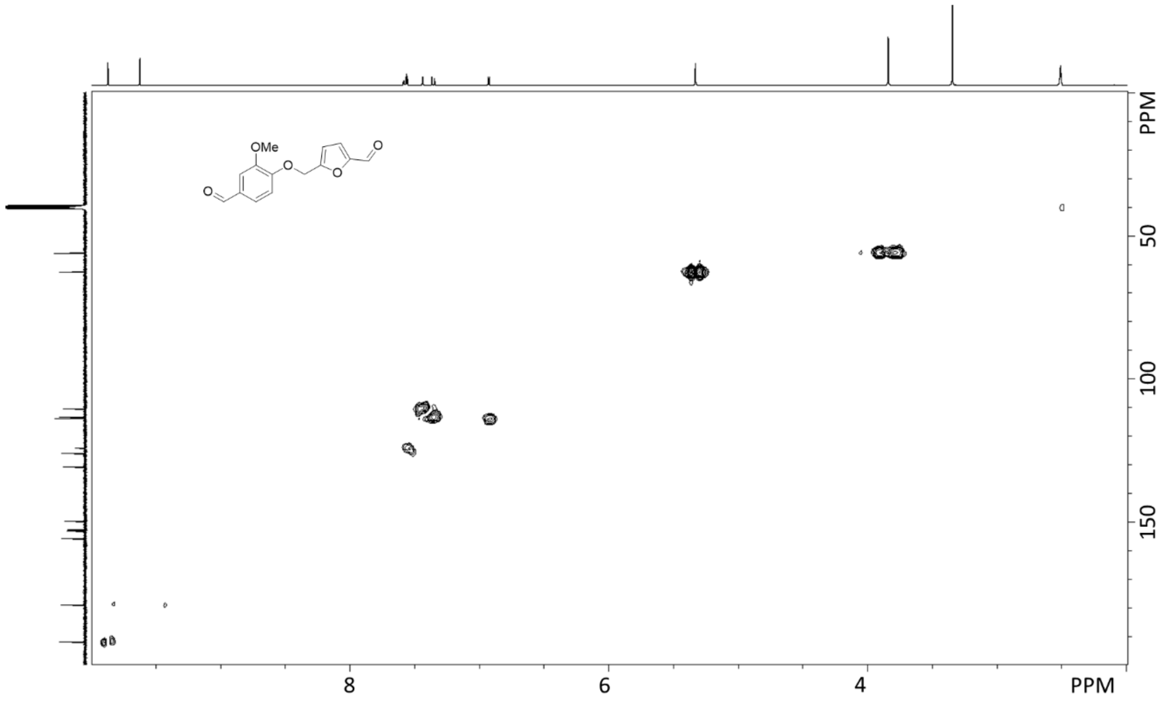

Figure S18. HMQC spectrum of **2b**.

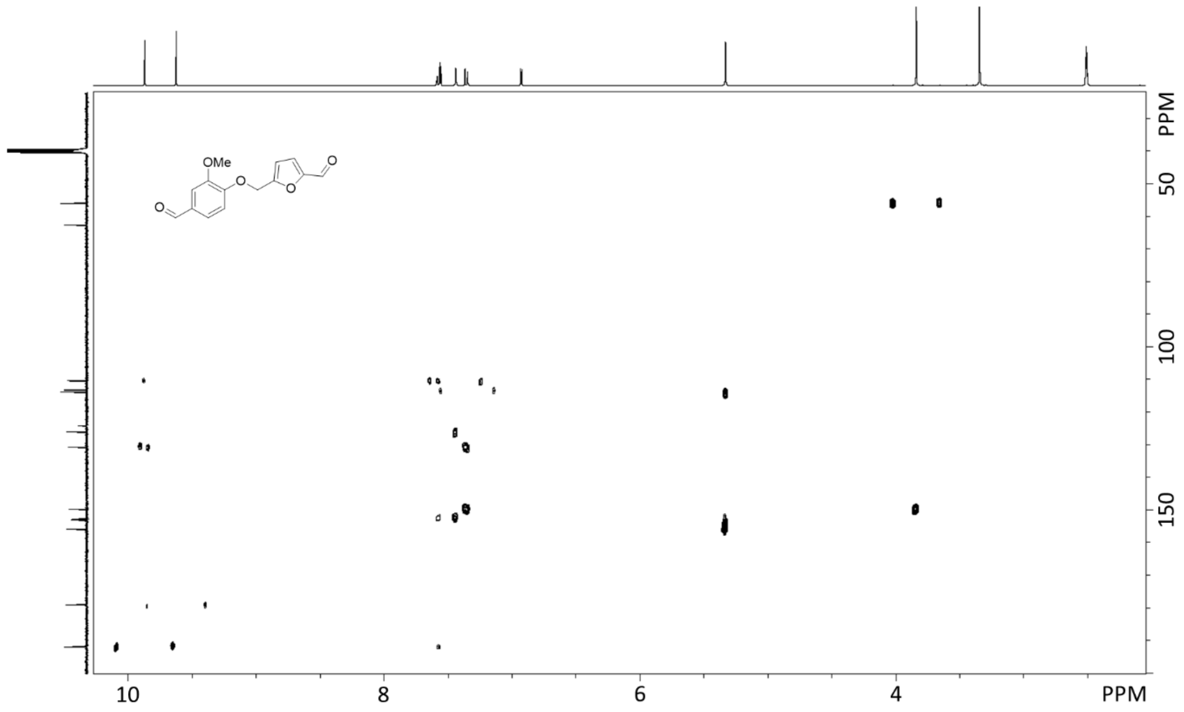

Figure S19. HMBC spectrum of **2b**.

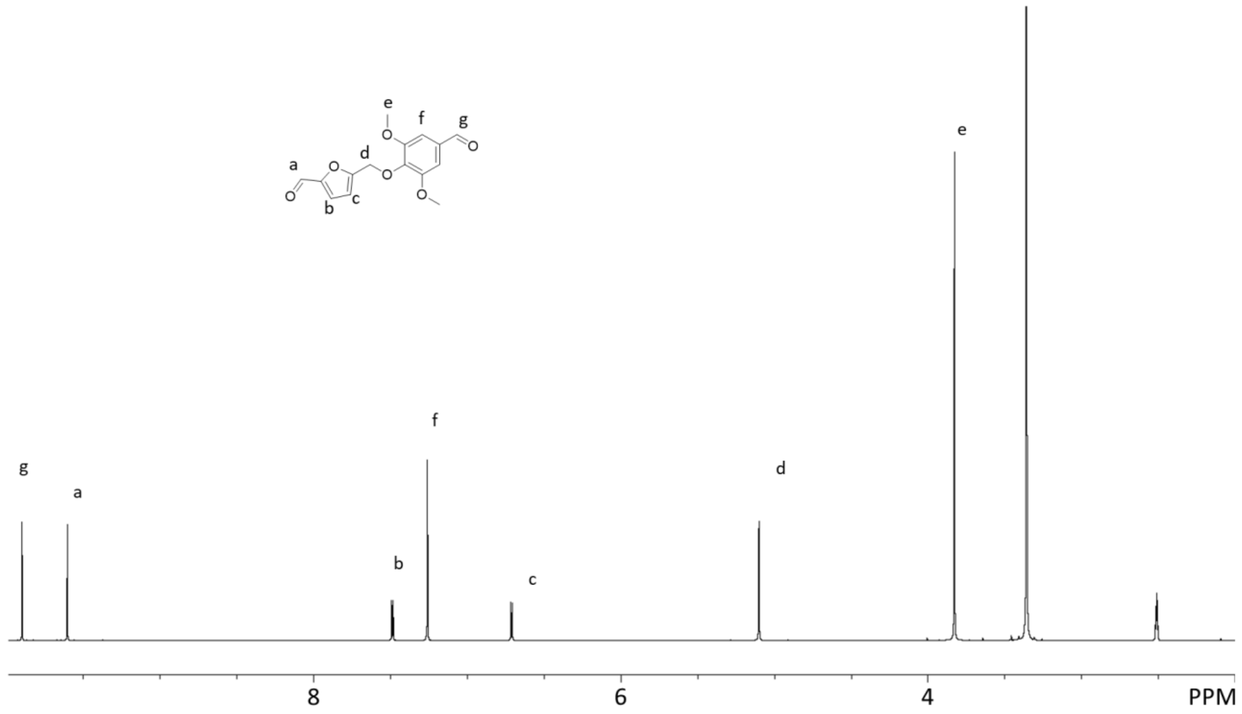

Figure S20. ^{1}H-NMR spectrum of **2c**.

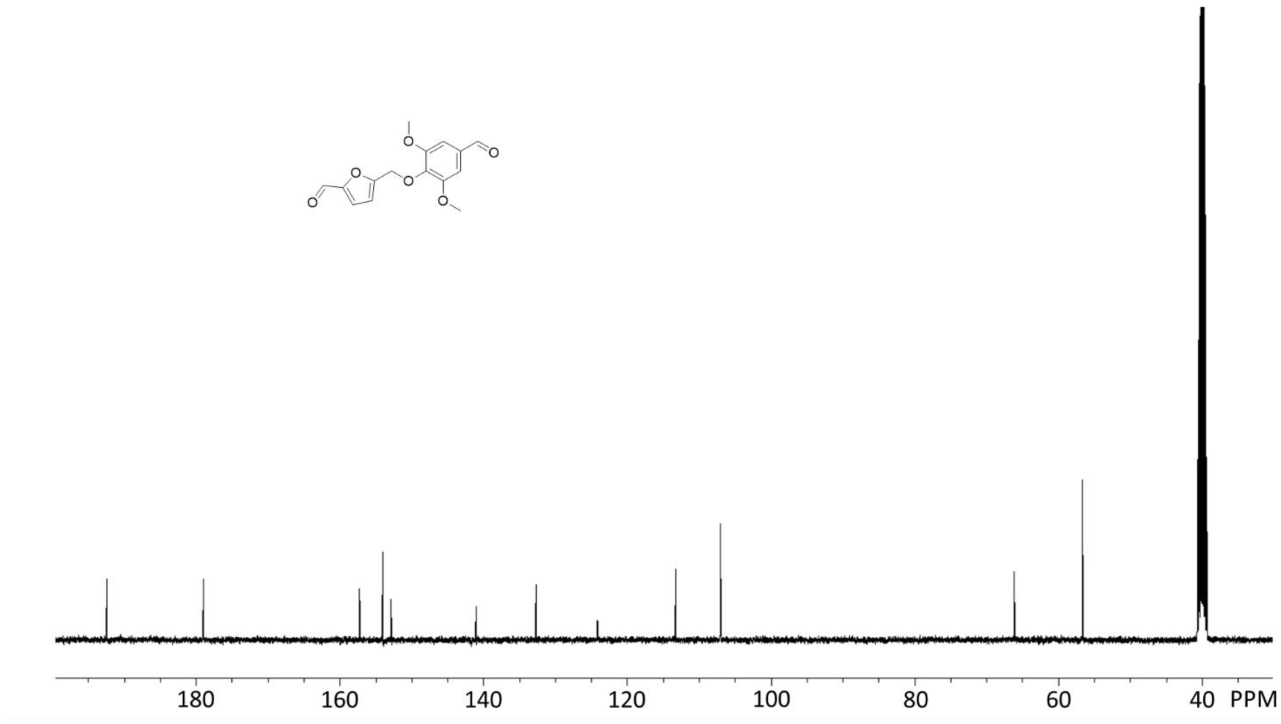

Figure S21. ^{13}C-NMR spectrum of **2c**.

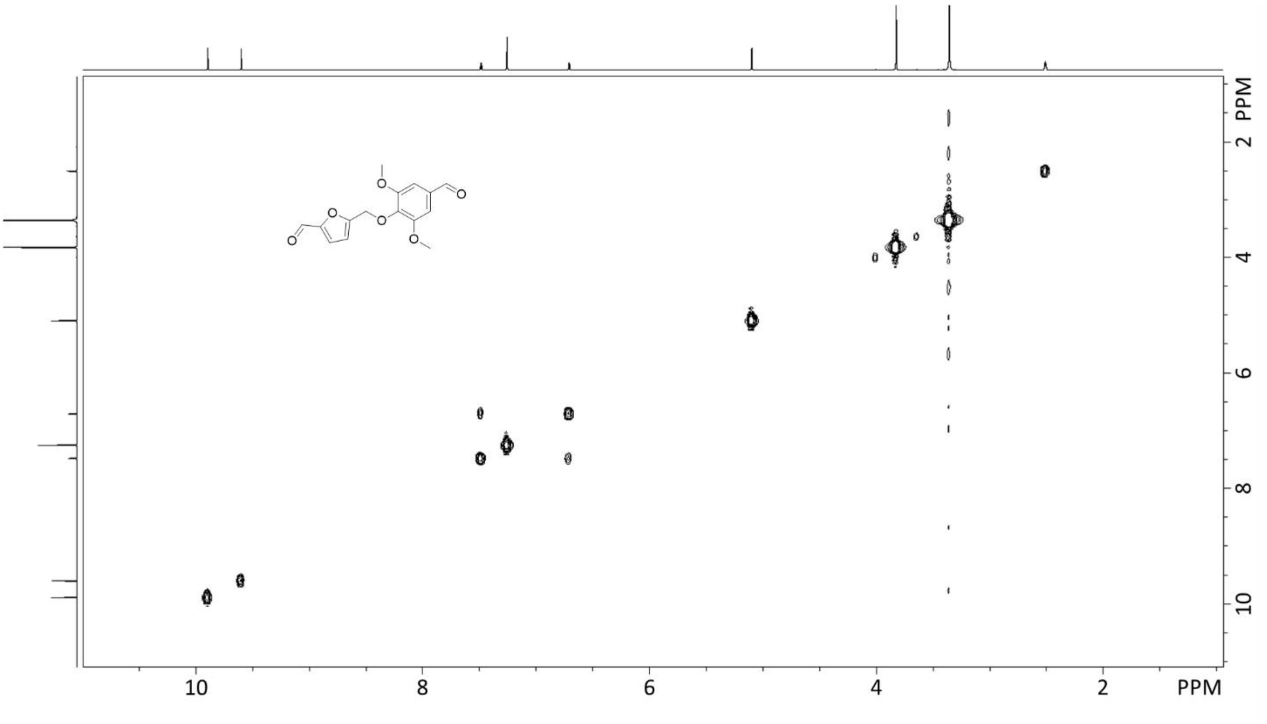

Figure S22. COSY spectrum of **2c**.

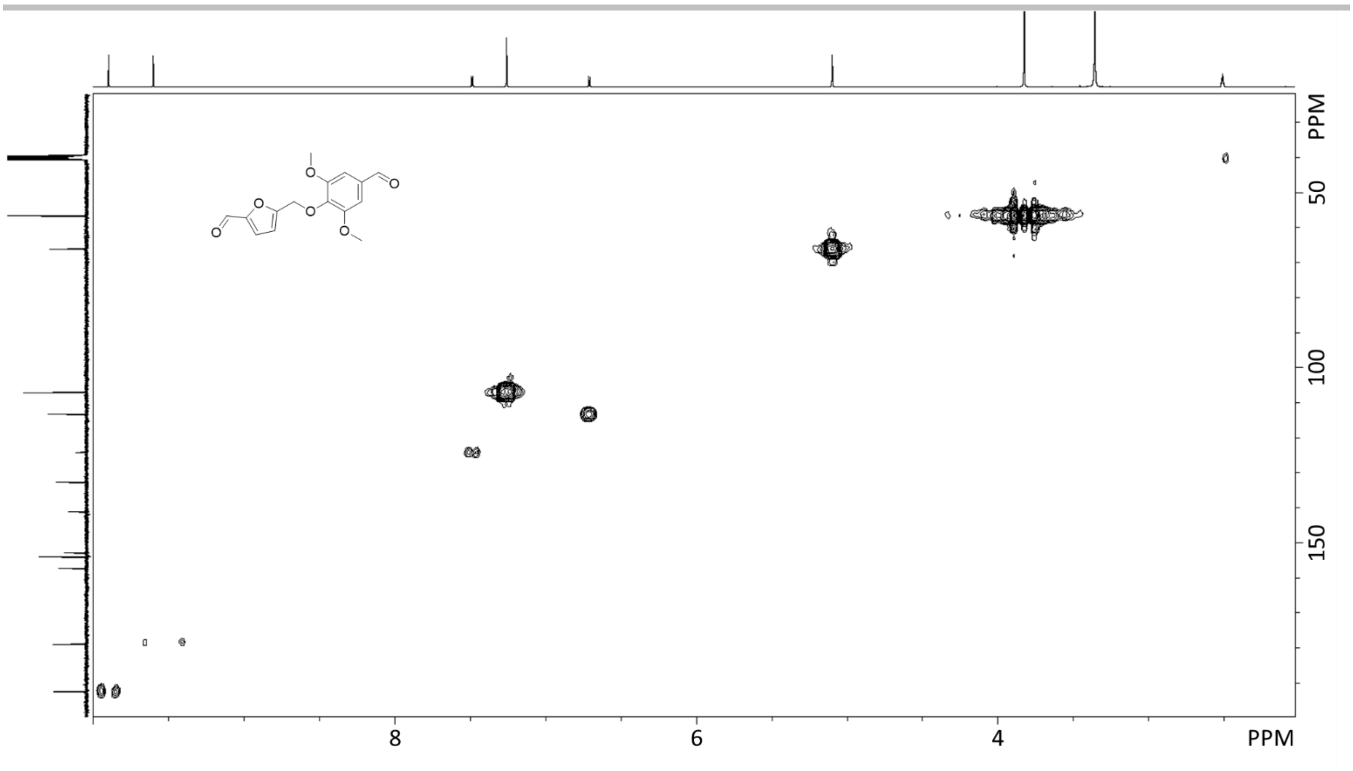

Figure S23. HMQC spectrum of **2c**.

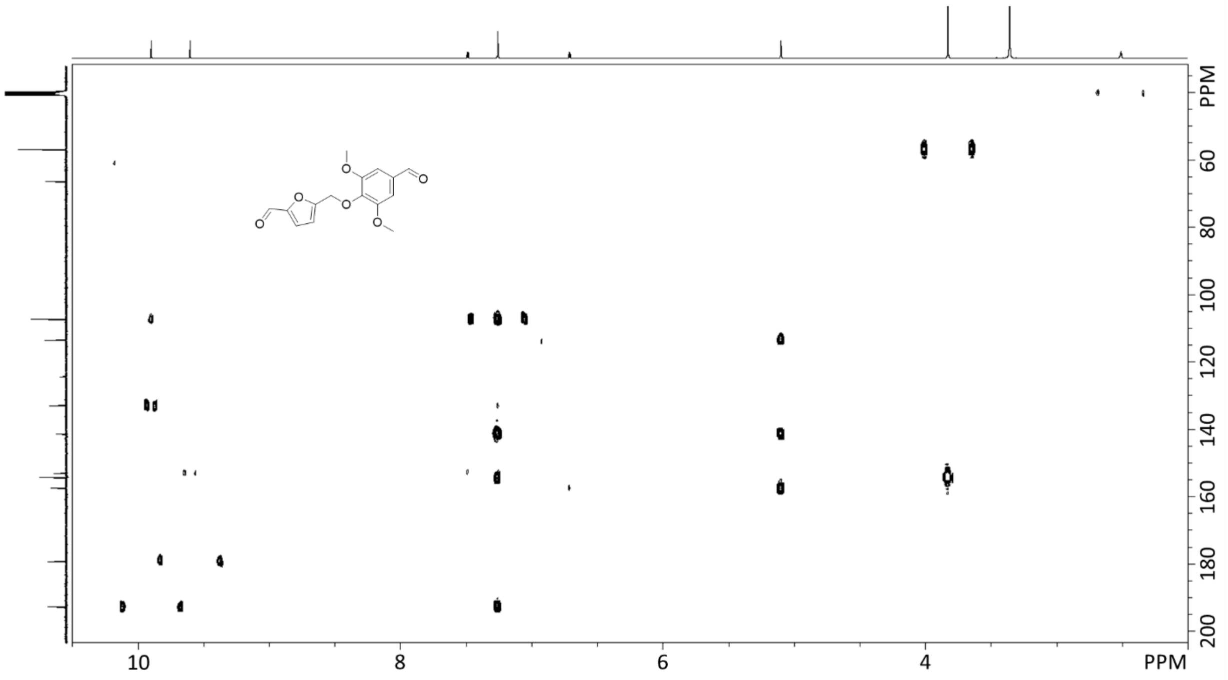

Figure S24. HMBC spectrum of **2c**.

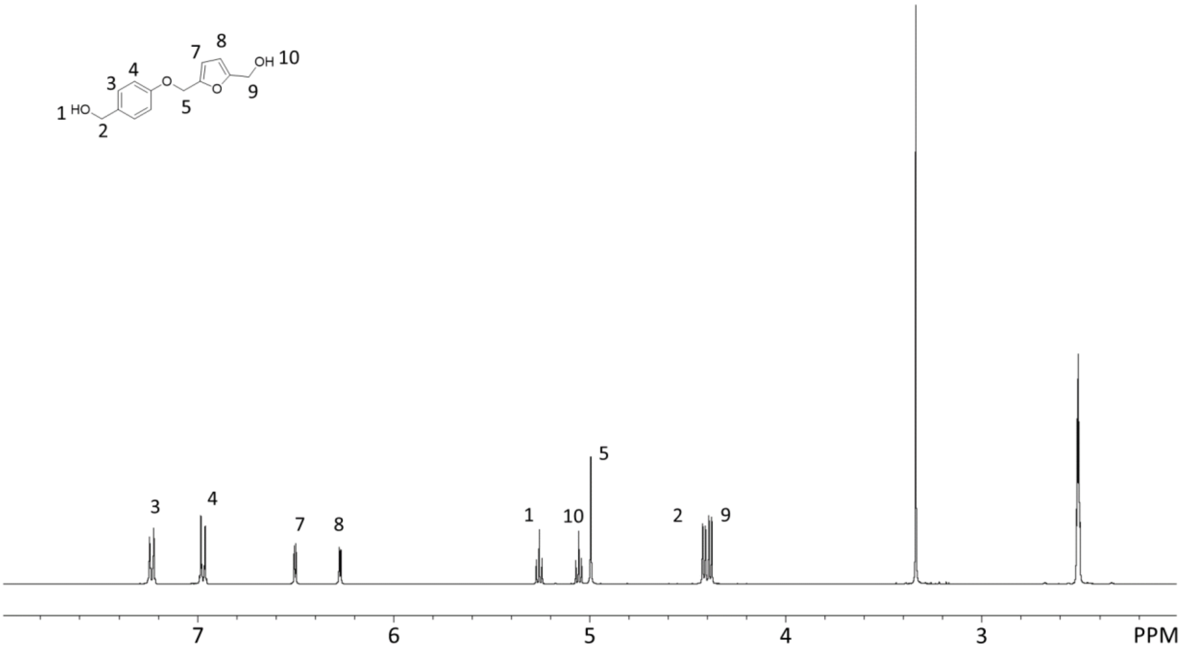

Figure S25. ^{1}H-NMR spectrum of **3a**.

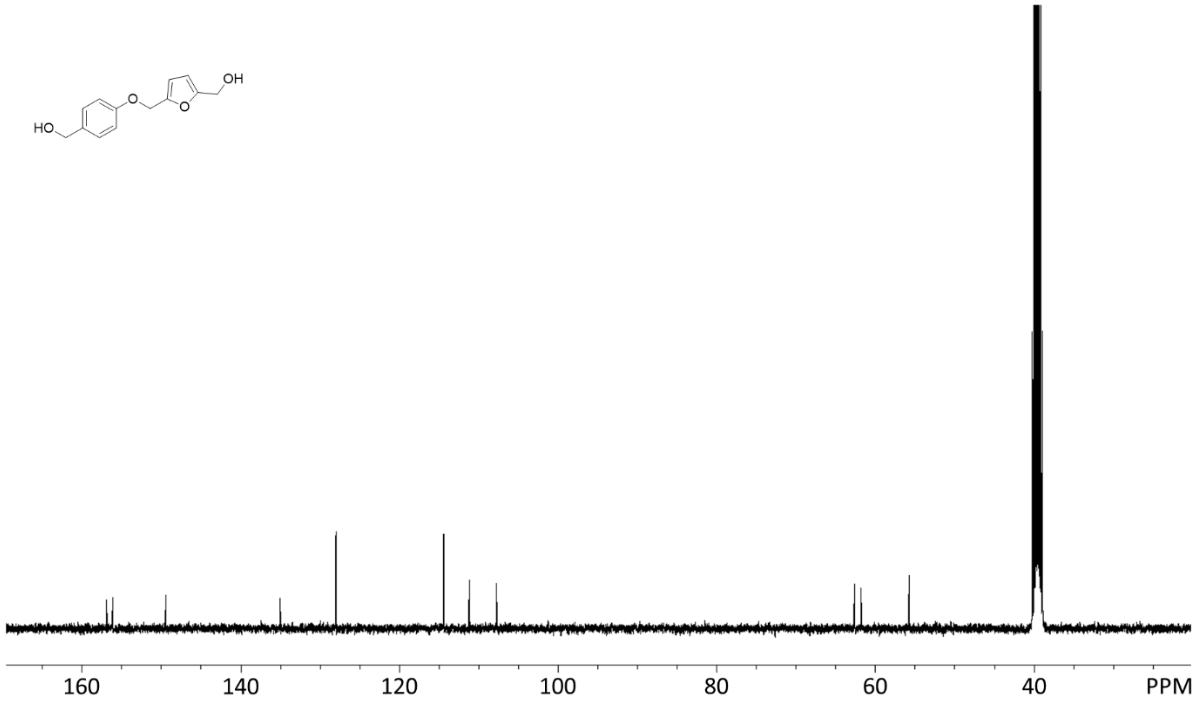

Figure S26. ^{13}C-NMR spectrum of **3a**.

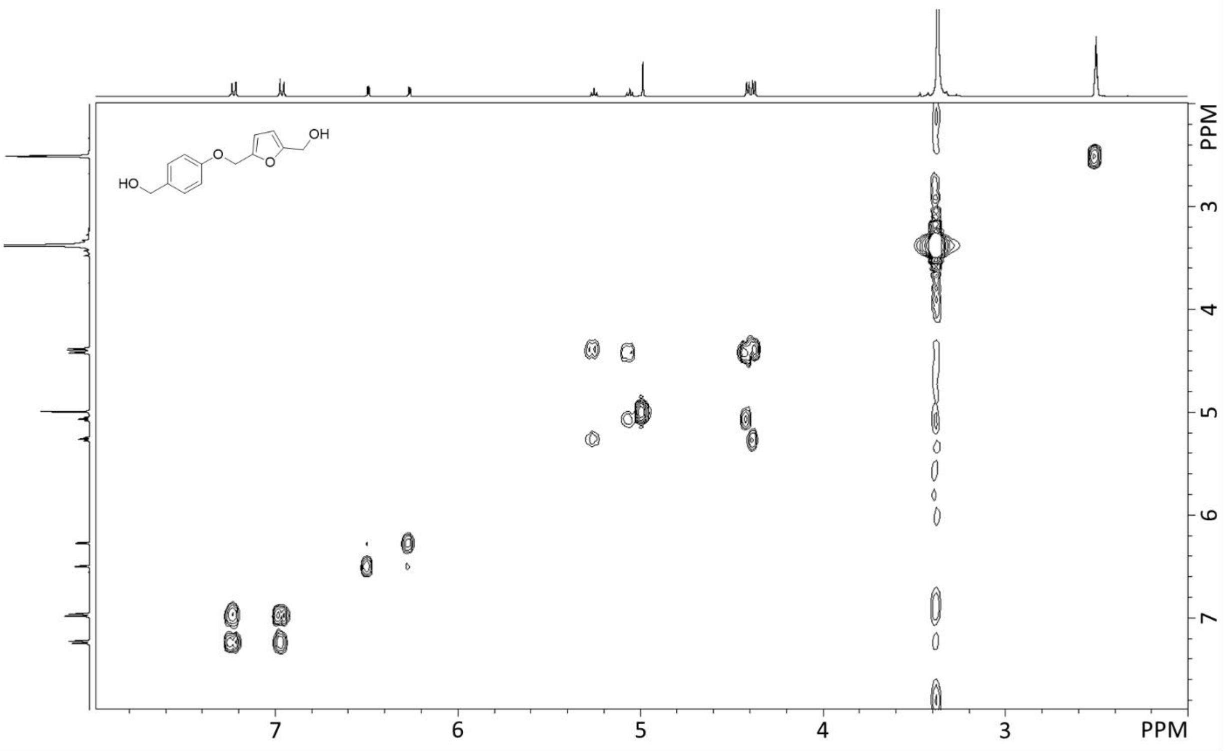

Figure S27. COSY spectrum of **3a**.

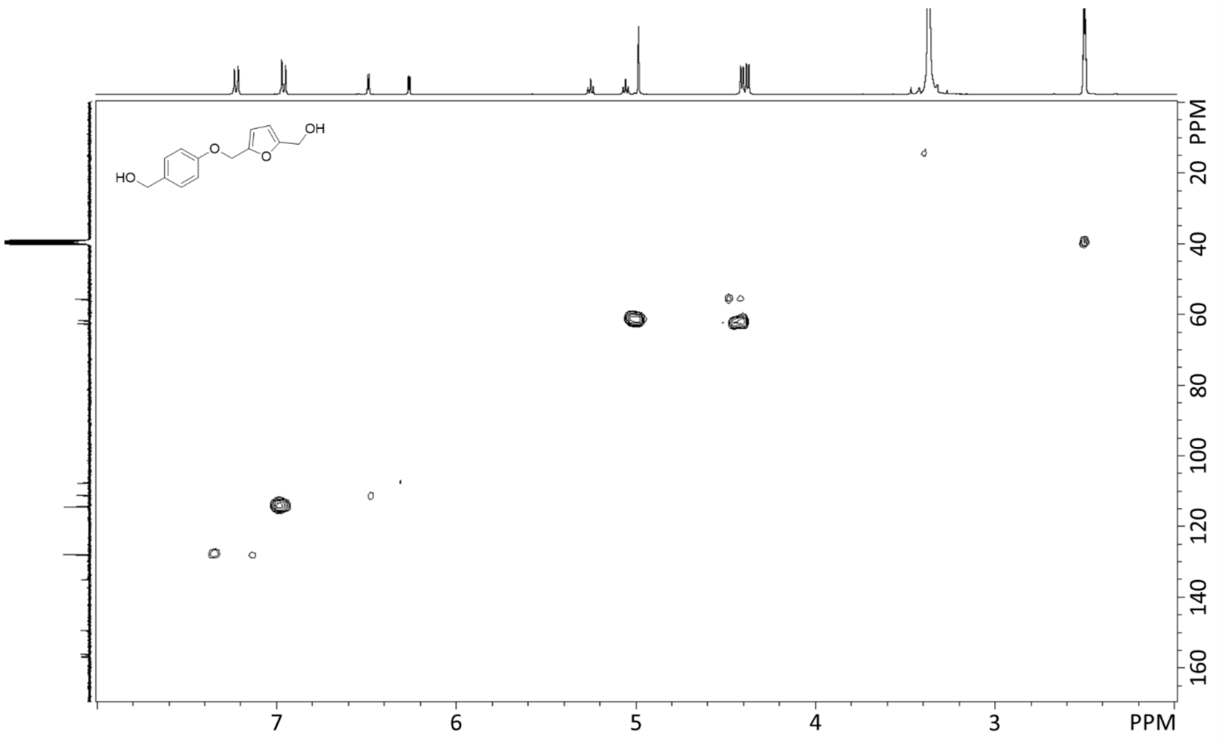

Figure S28. HMQC spectrum of **3a**.

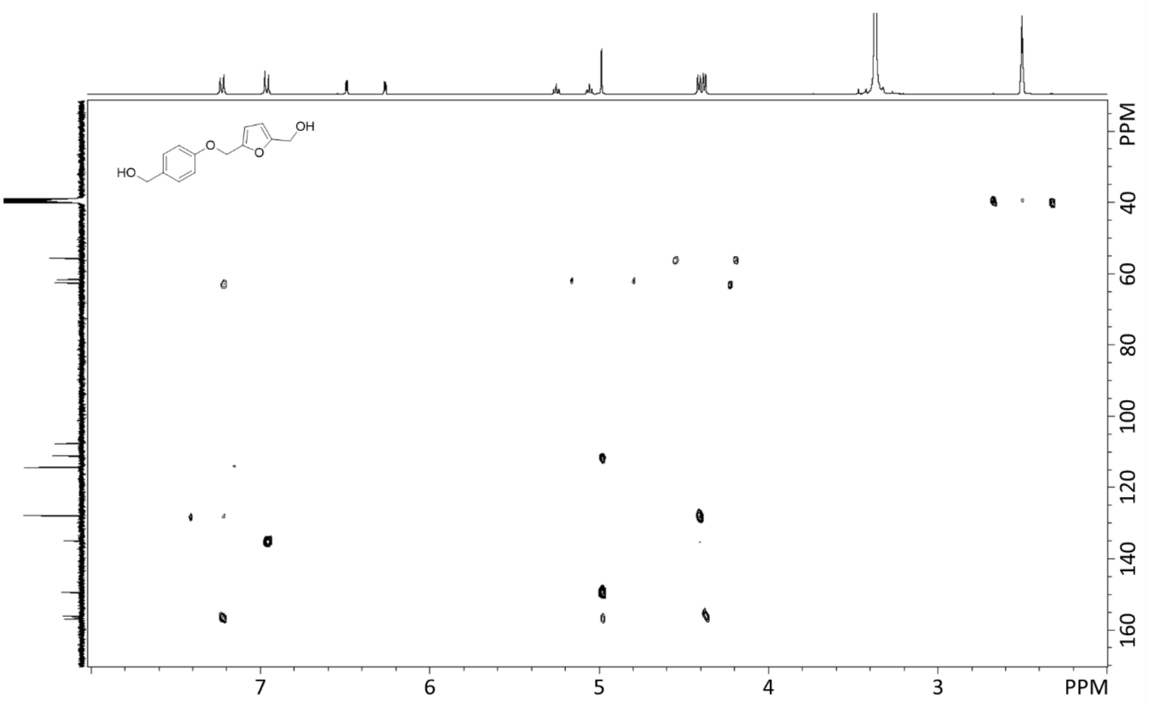

Figure S29. HMBC spectrum of **3a**.

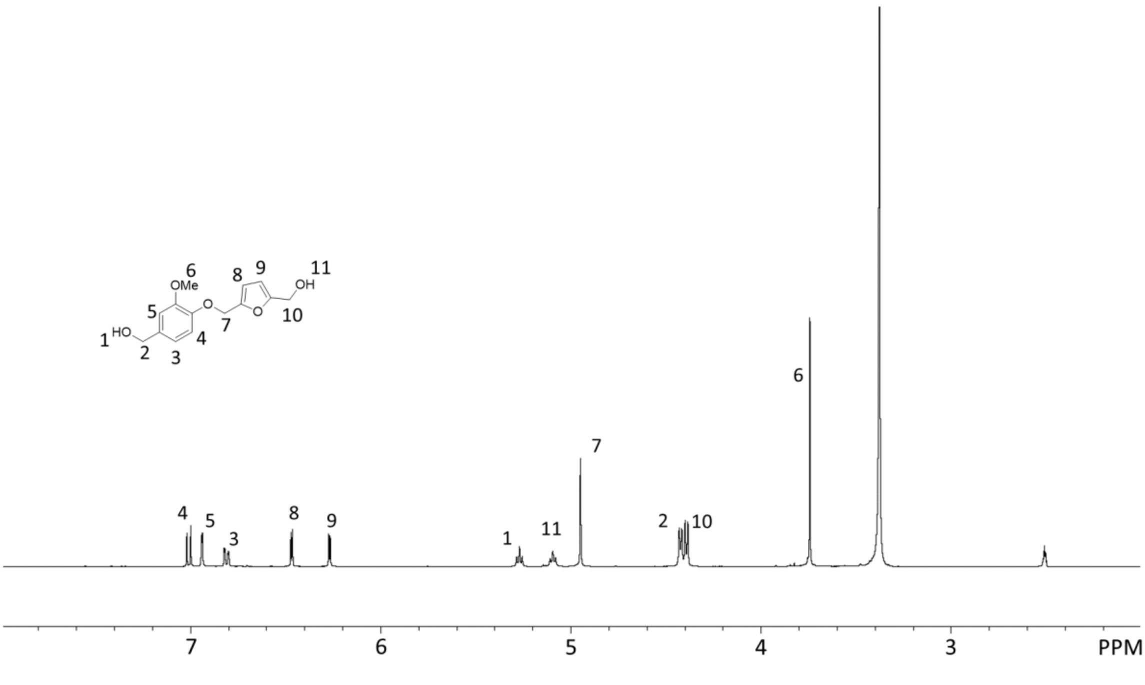

Figure S30. ^{1}H-NMR spectrum of **3b**.

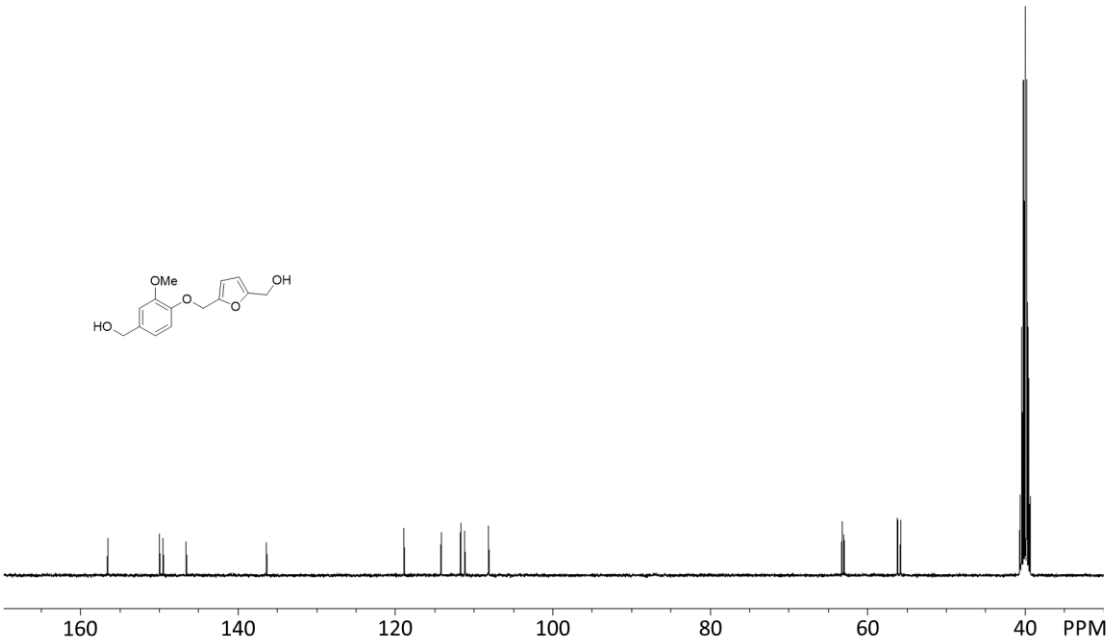

Figure S31. ^{13}C-NMR spectrum of **3b**.

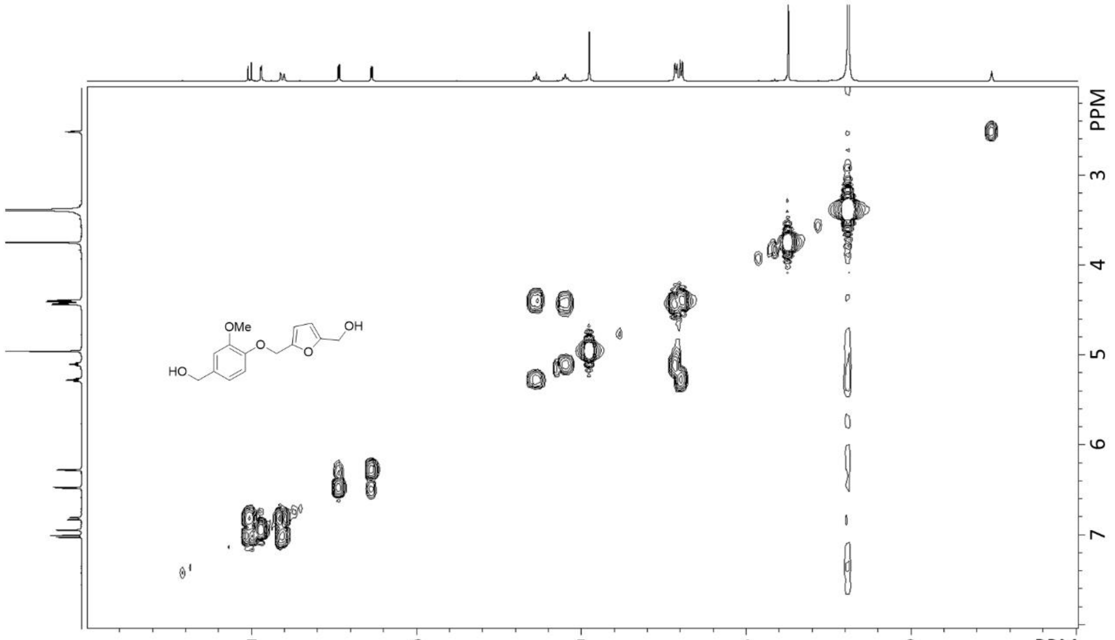

Figure S32. COSY spectrum of **3b**.

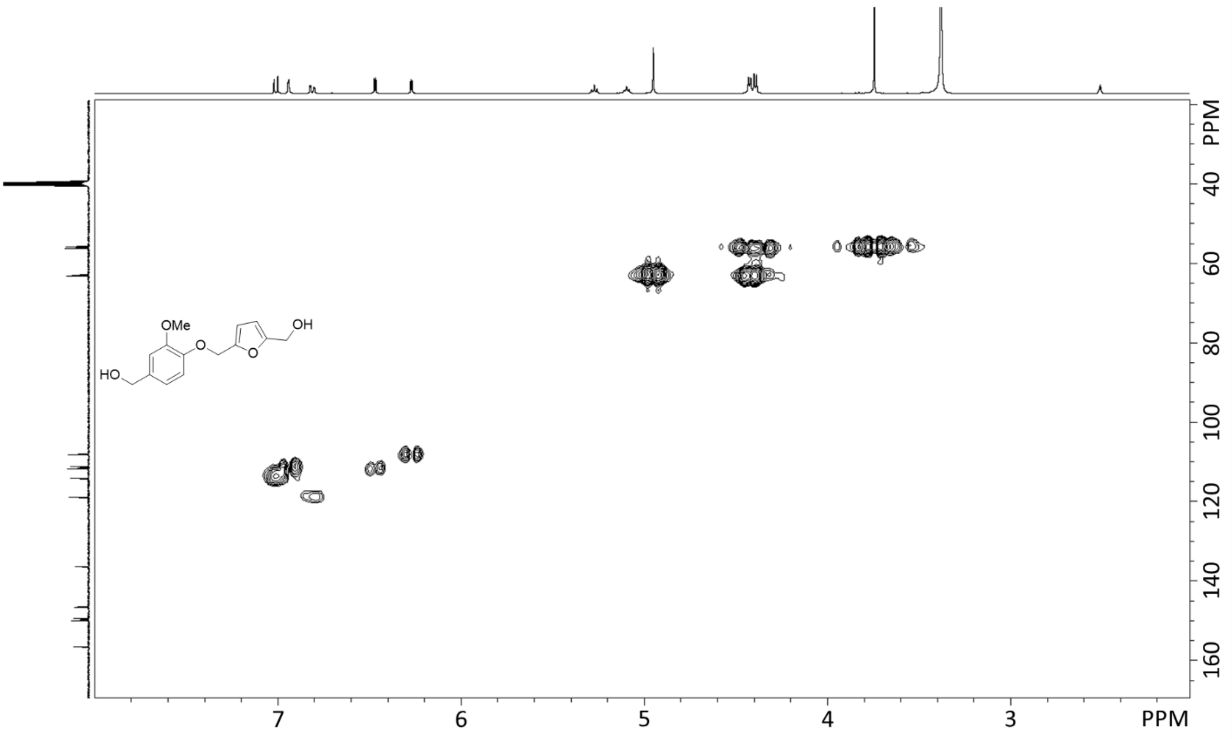

Figure S33. HMQC spectrum of **3b**.

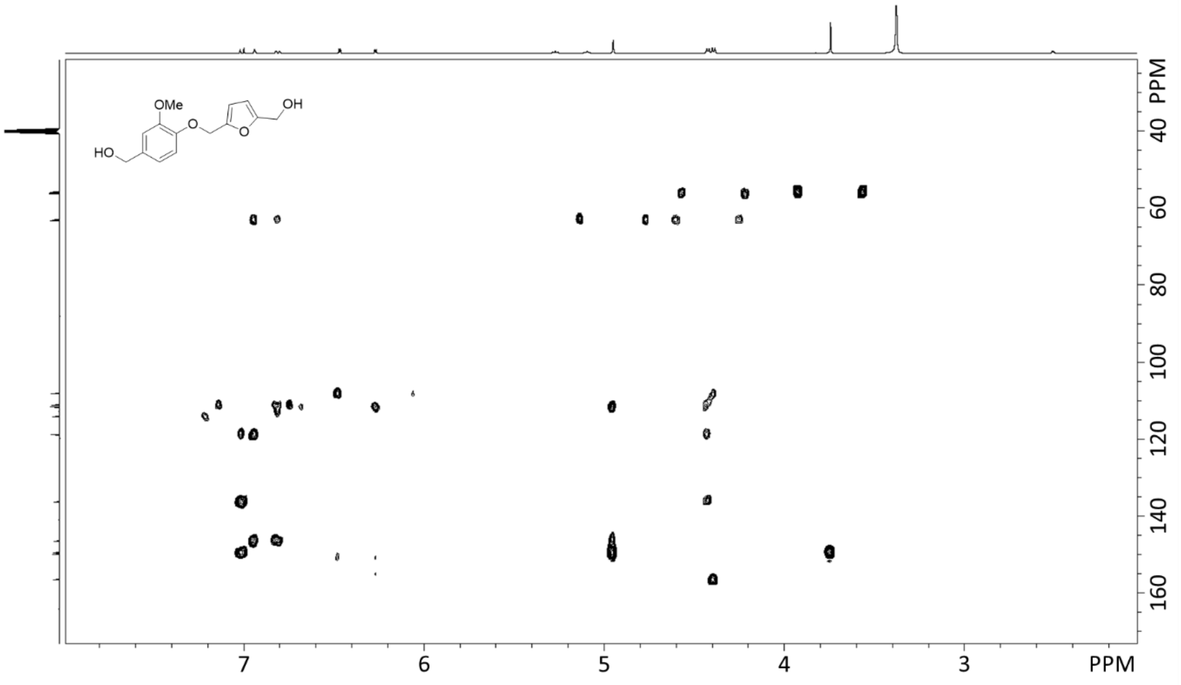

Figure S34. HMBC spectrum of **3b**.

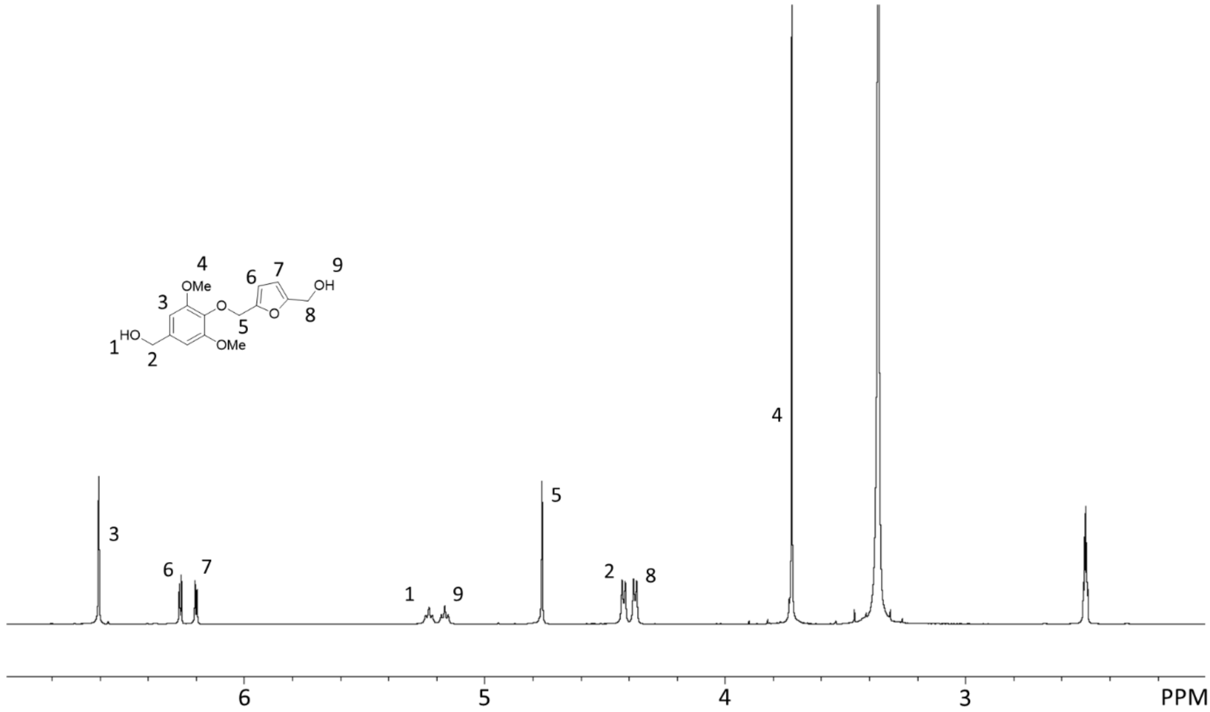

Figure S35. ^{1}H-NMR spectrum of **3c**.

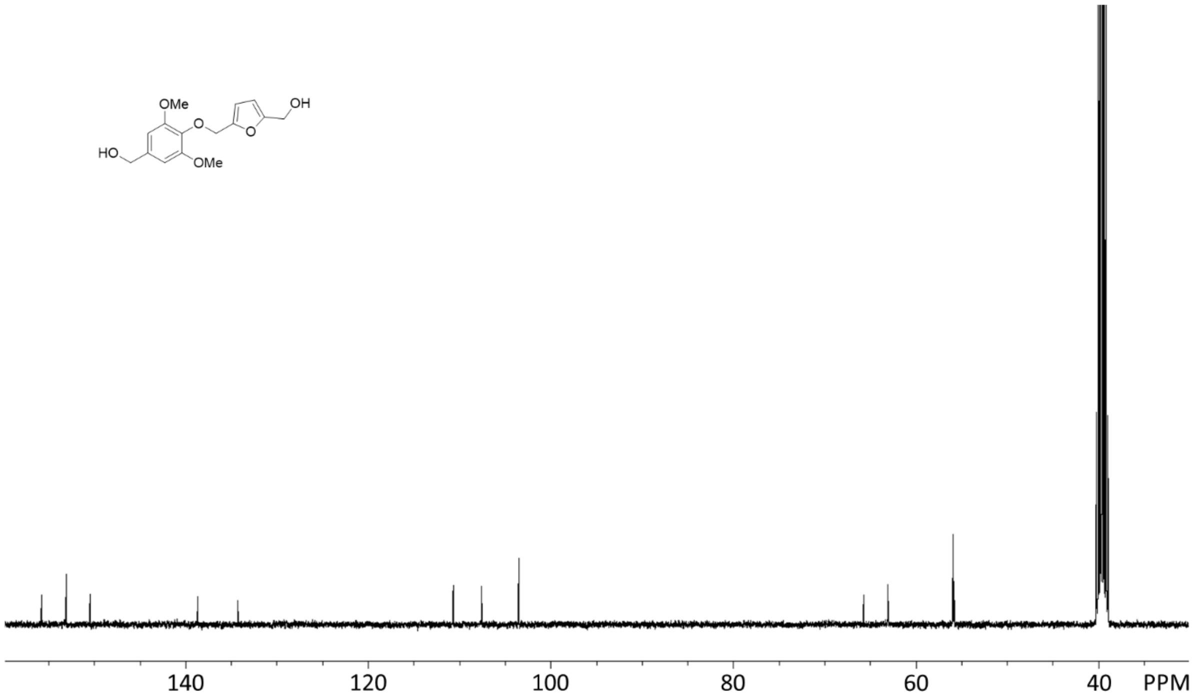

Figure S36. ^{13}C-NMR spectrum of **3c**.

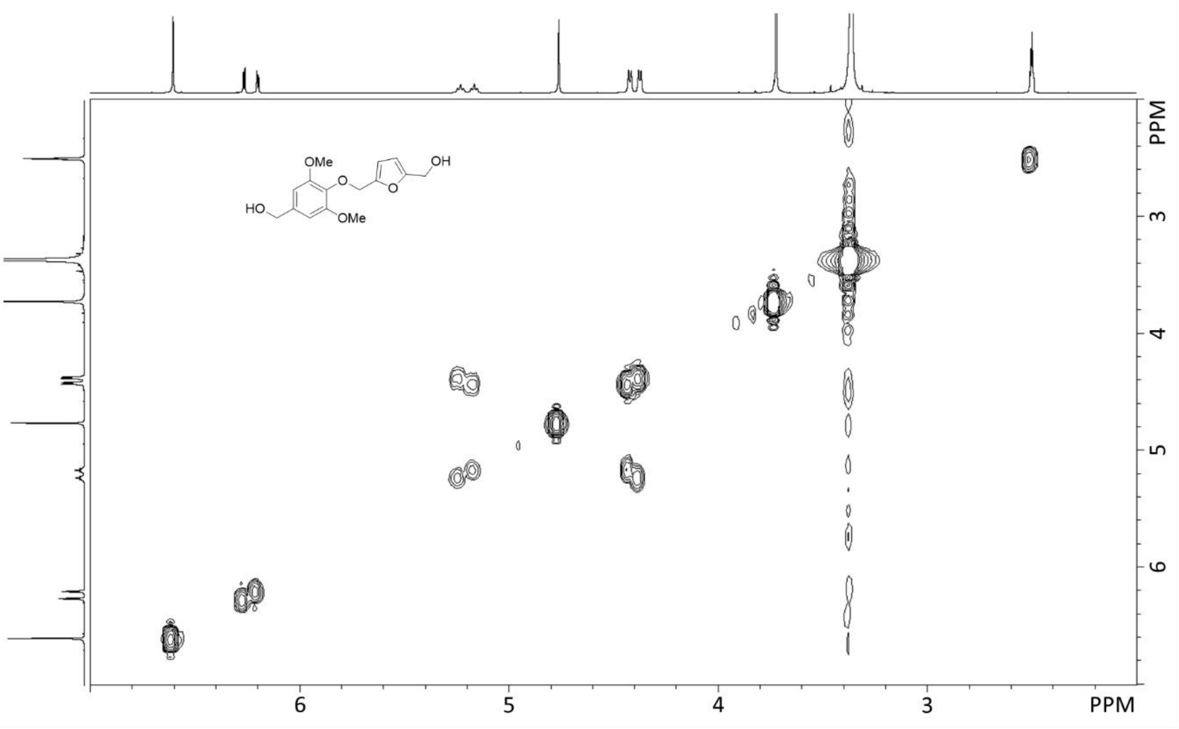

Figure S37. COSY spectrum of **3c**.

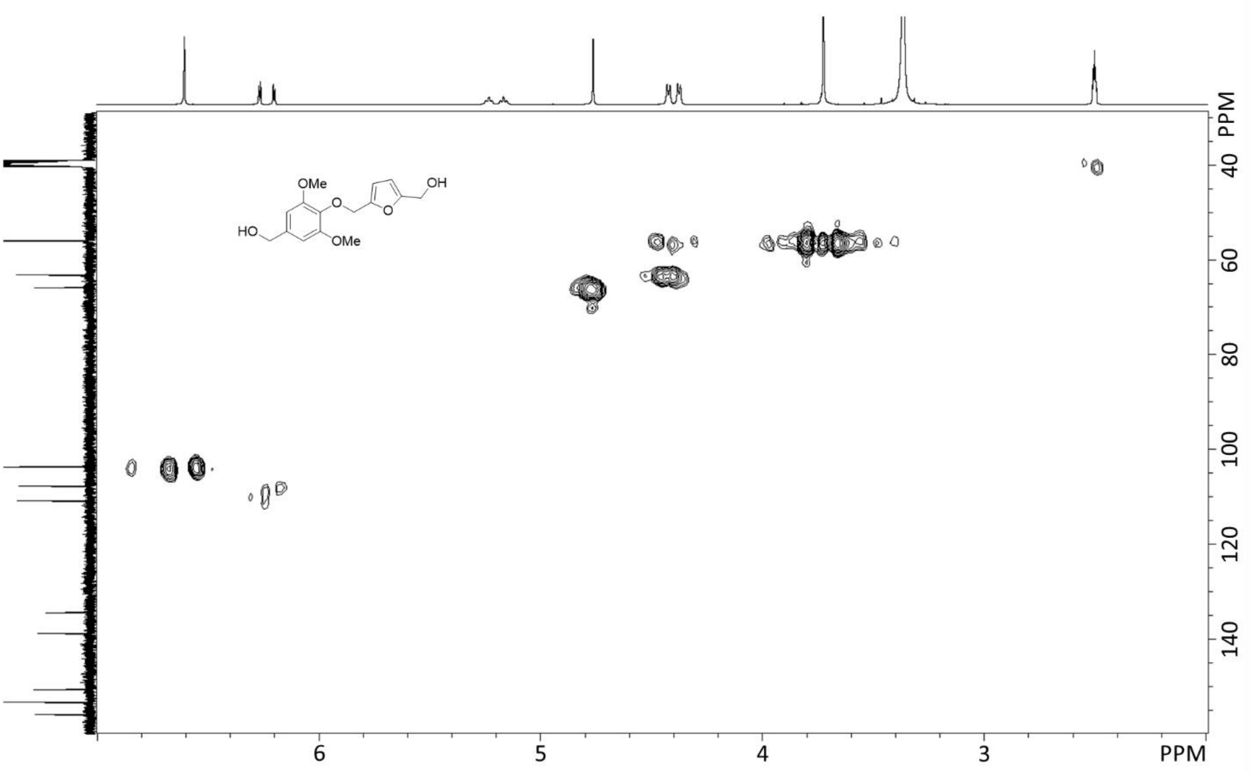

Figure S38. HMQC spectrum of **3c**.

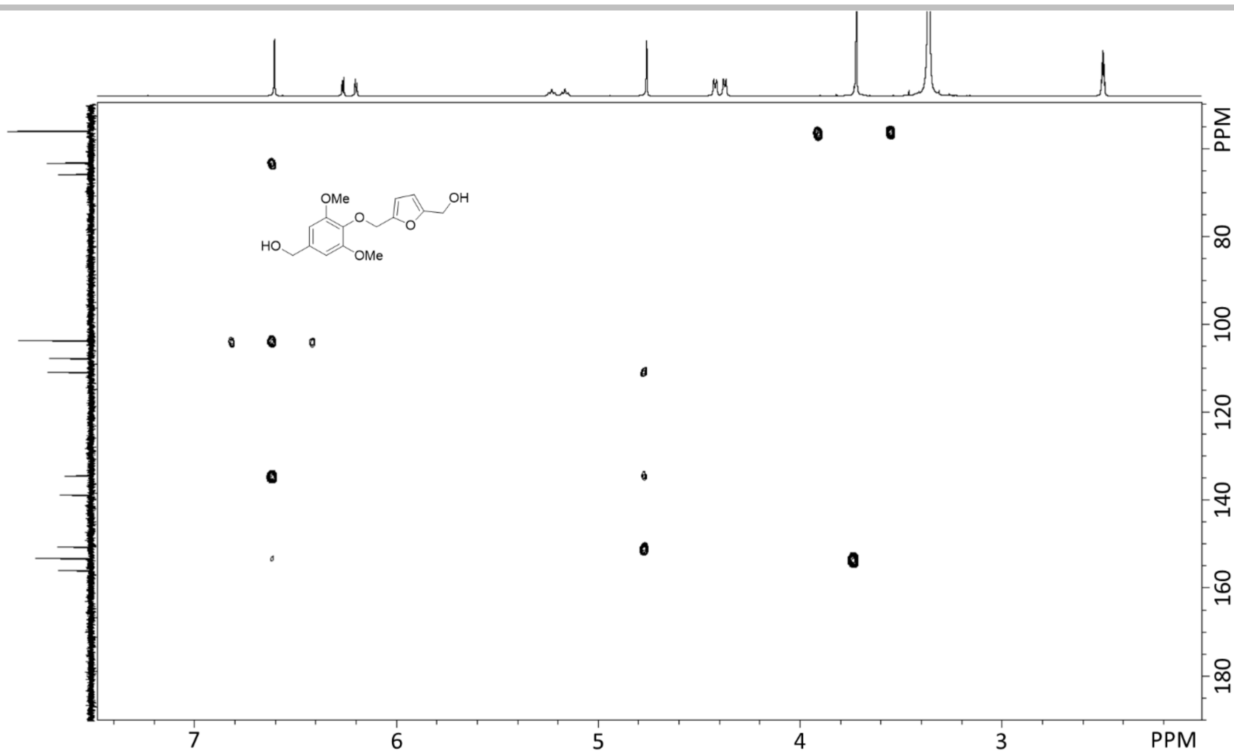

Figure S39. HMBC spectrum of **3c**.

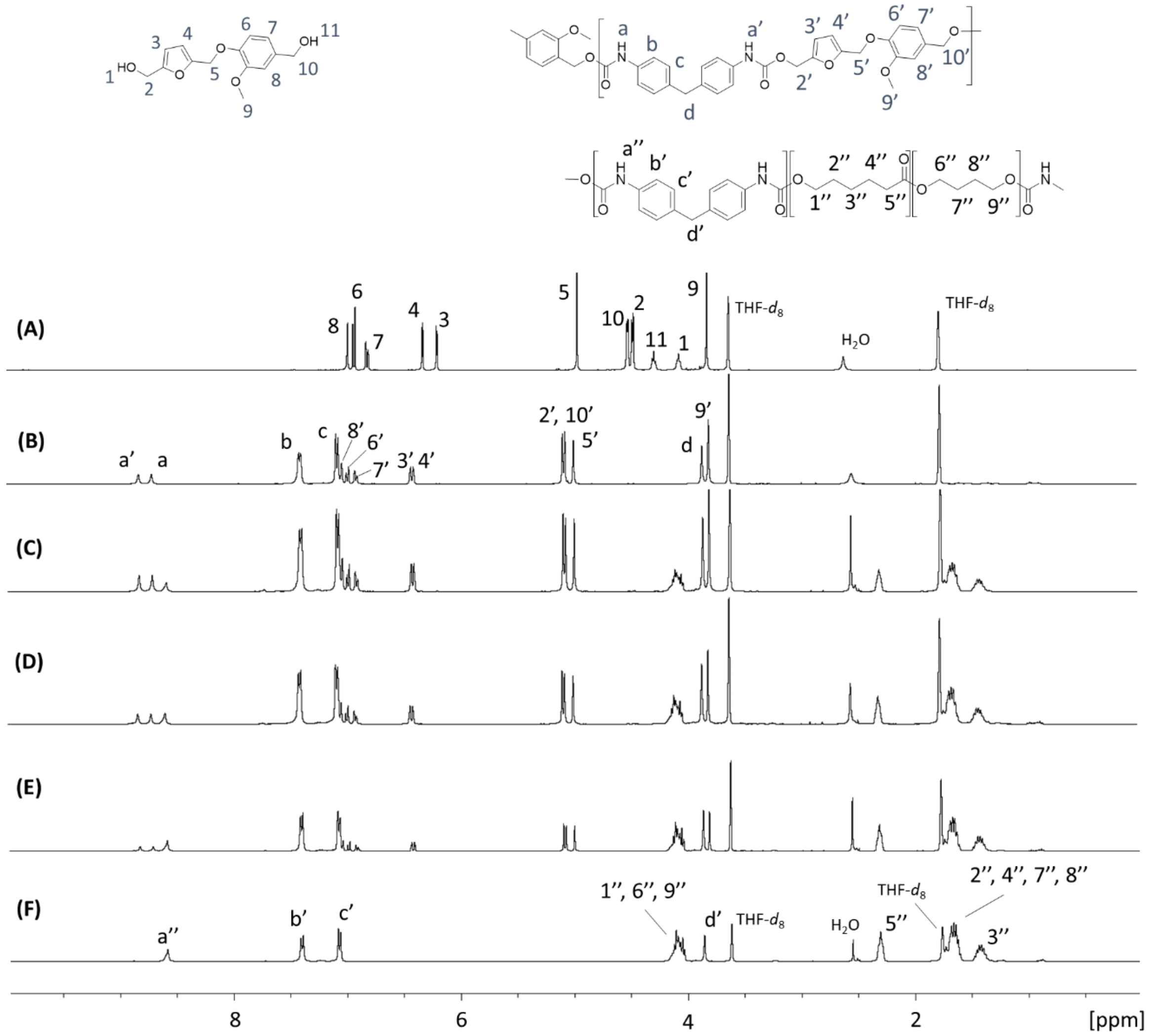

Figure S40. ^{1}H NMR spectra recorded in THF-d_8 of **3b** (A), **PU-3b** (B), **PU-3b$_{67}$C$_{33}$** (C), **PU-3b$_{50}$C$_{50}$** (D), **PU-3b$_{33}$C$_{67}$** (E), **PU-C** (F).

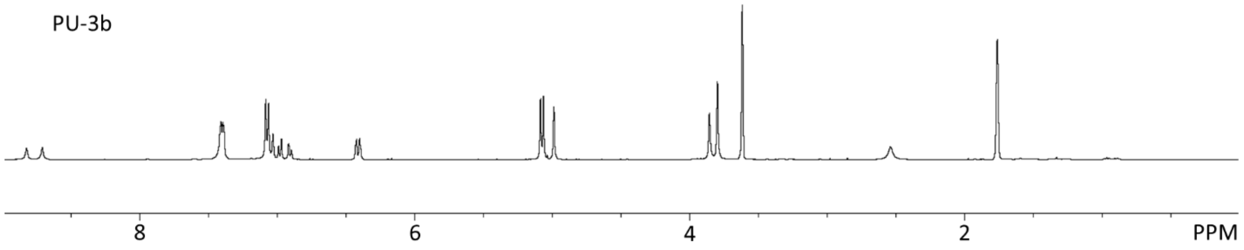

Figure S41. ^{1}H-NMR spectrum of **PU-3b**.

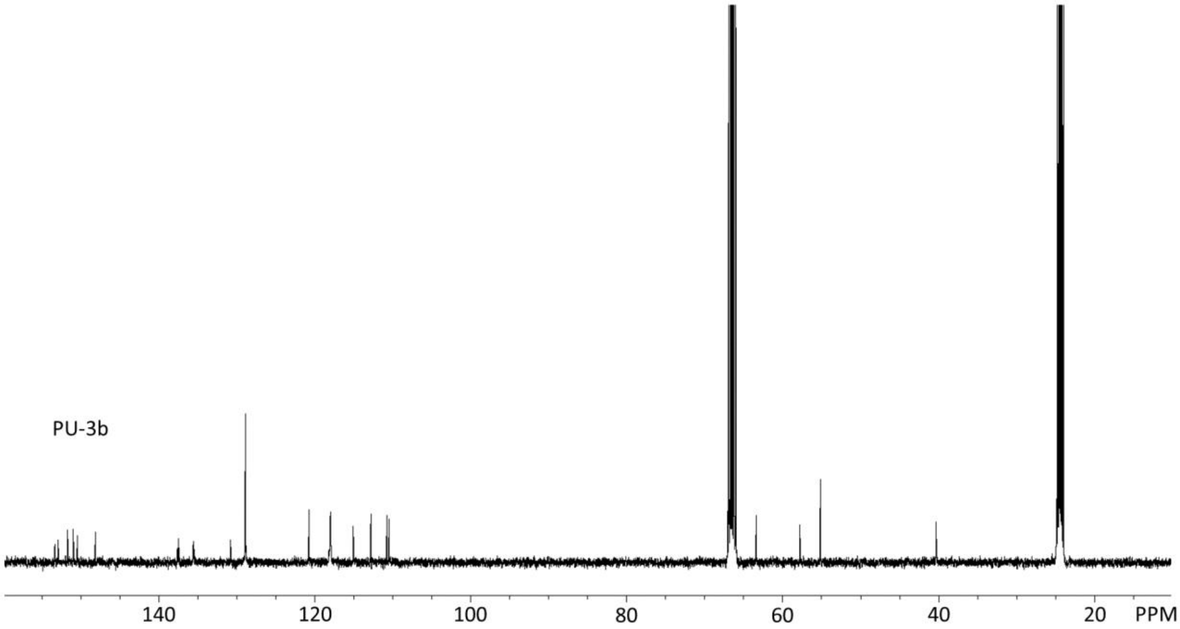

Figure S42. ^{13}C-NMR spectrum of **PU-3b**.

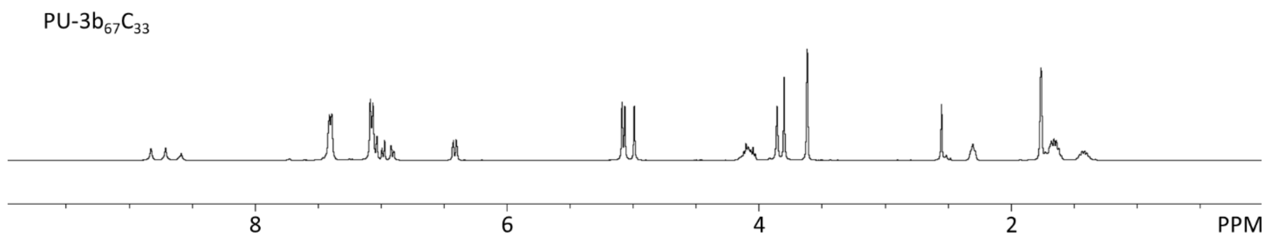

Figure S43. ^{1}H-NMR spectrum of **PU-3b$_{67}$C$_{33}$**.

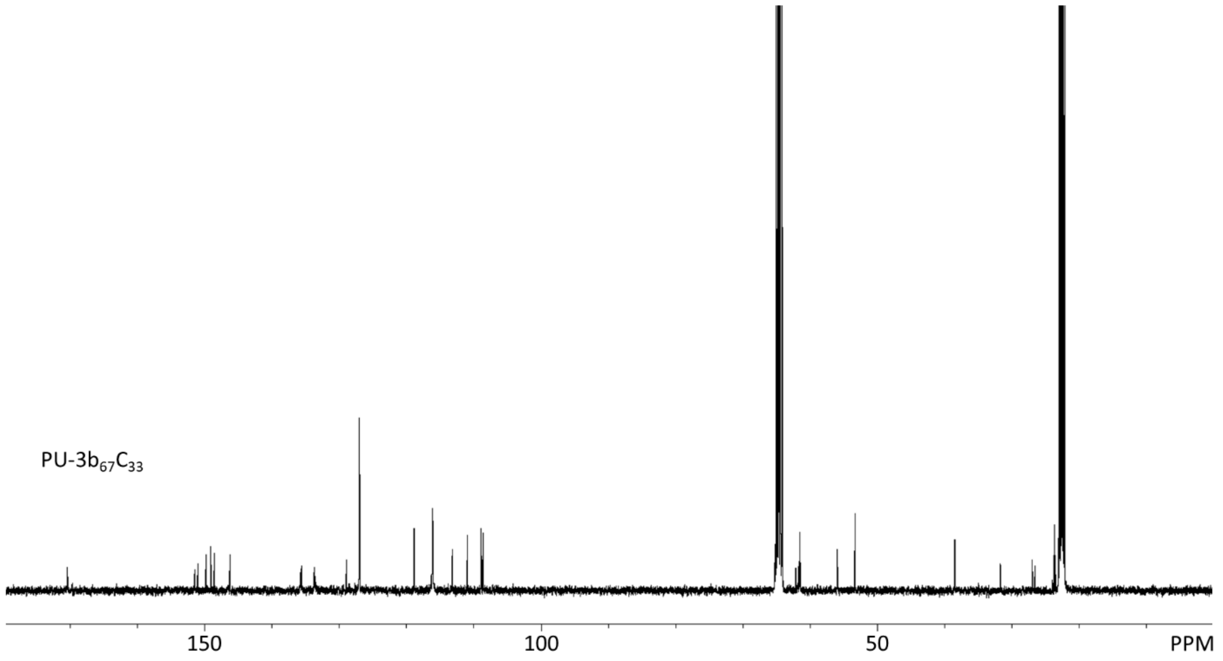

Figure S44. ^{13}C-NMR spectrum of **PU-3b$_{67}$C$_{33}$**.

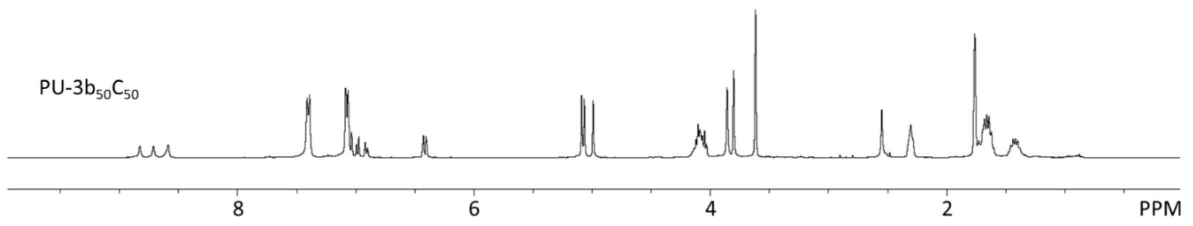

Figure S45. ^{1}H-NMR spectrum of **PU-3b$_{50}$C$_{50}$**.

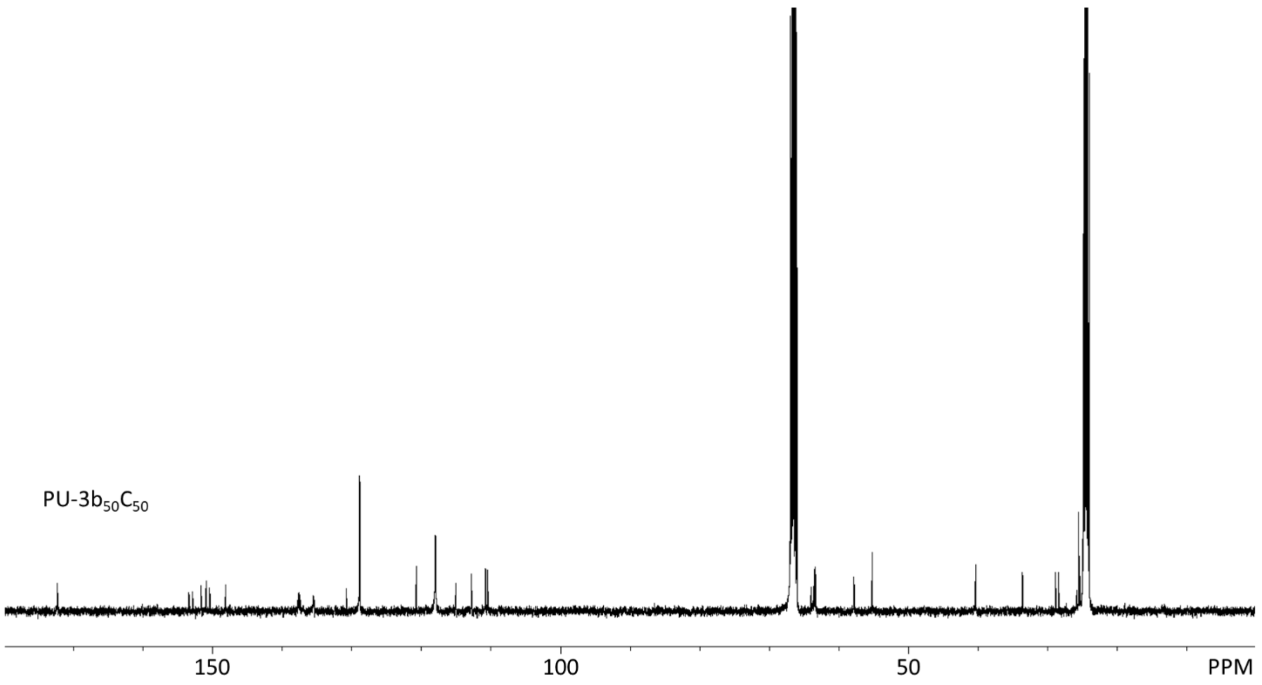

Figure S46. ^{13}C-NMR spectrum of **PU-3b$_{50}$C$_{50}$**.

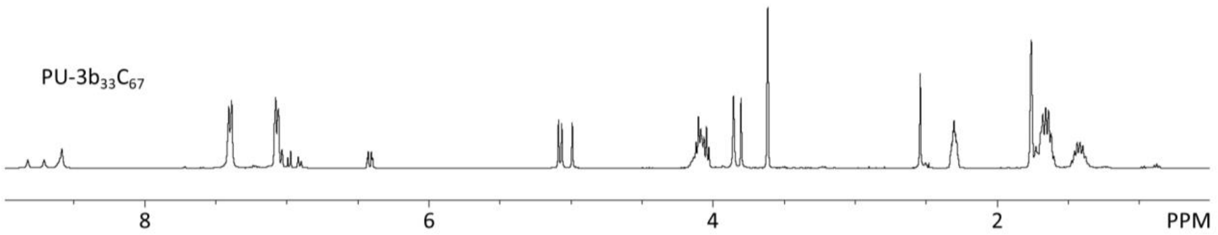

Figure S47. ^{1}H-NMR spectrum of **PU-3b$_{33}$C$_{67}$.**

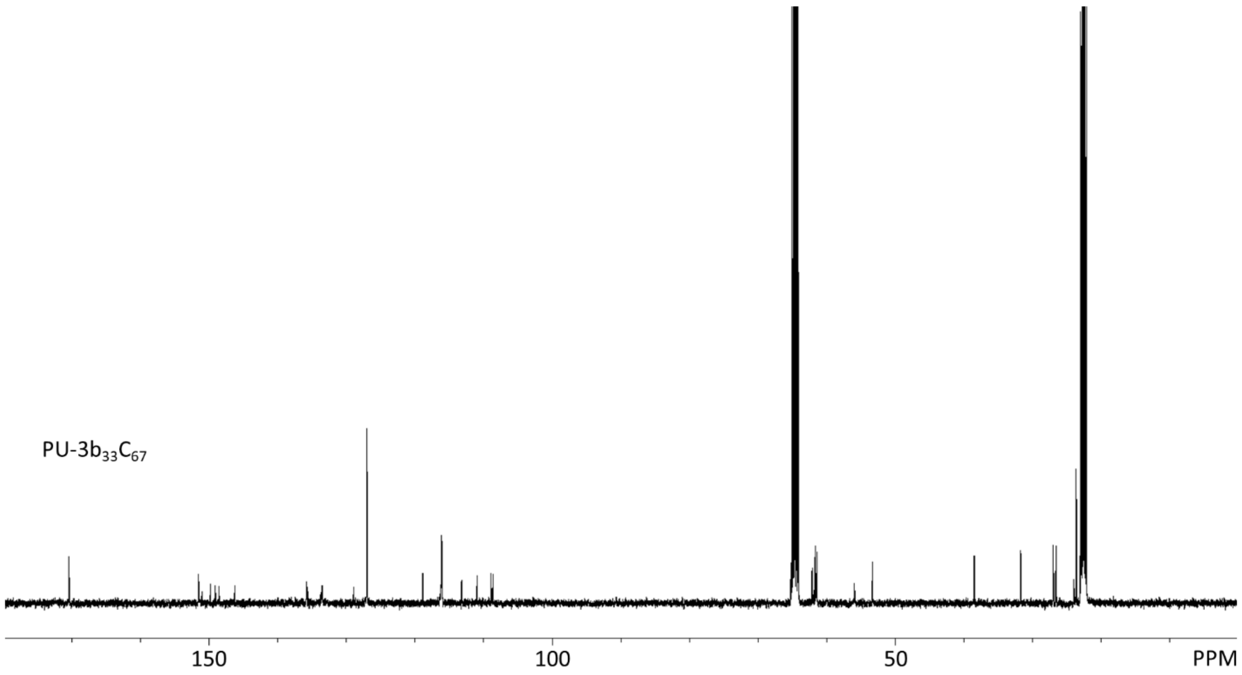

Figure S48. ^{13}C-NMR spectrum of **PU-3b$_{33}$C$_{67}$**.

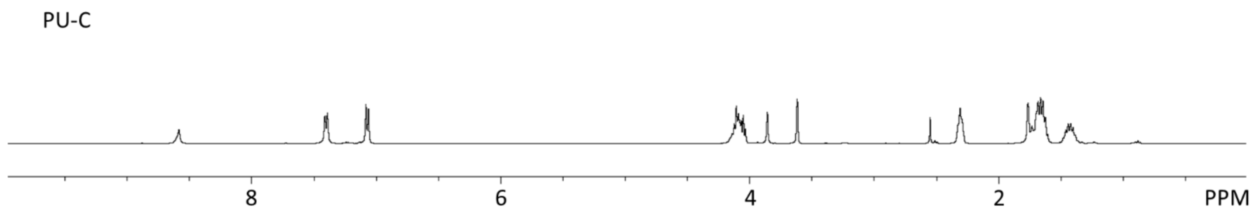

Figure S49. ^{1}H-NMR spectrum of **PU-C**.

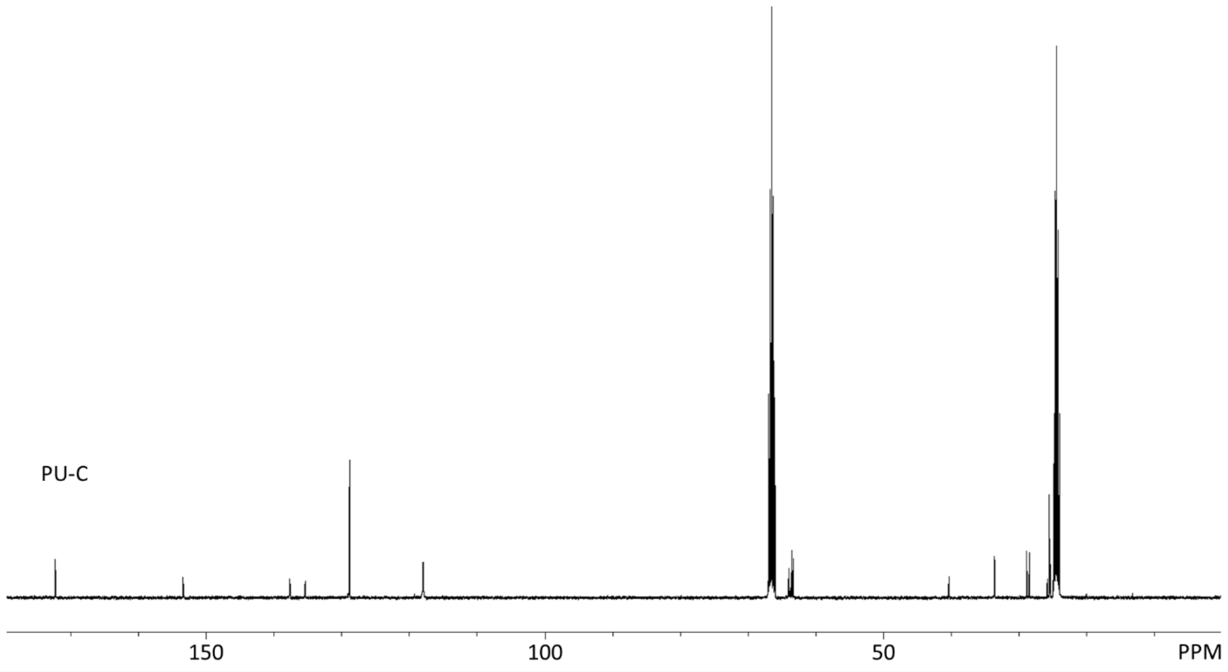

Figure S50. ^{13}C-NMR spectrum of **PU-C**.

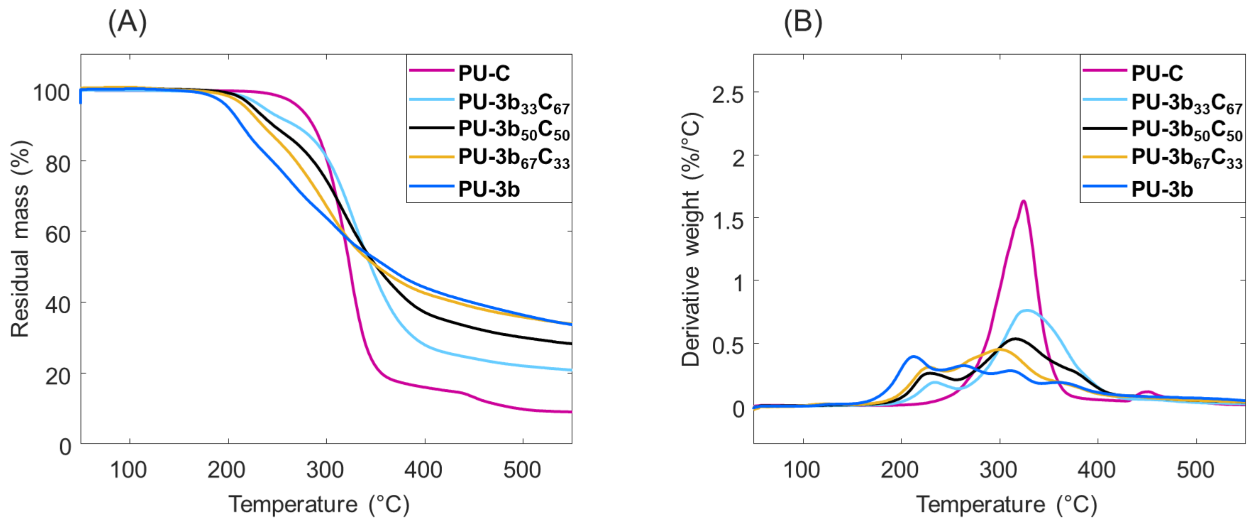

Figure S51. (A) TGA thermograms and (B) 1st derivative curves of the series of polyurethanes.

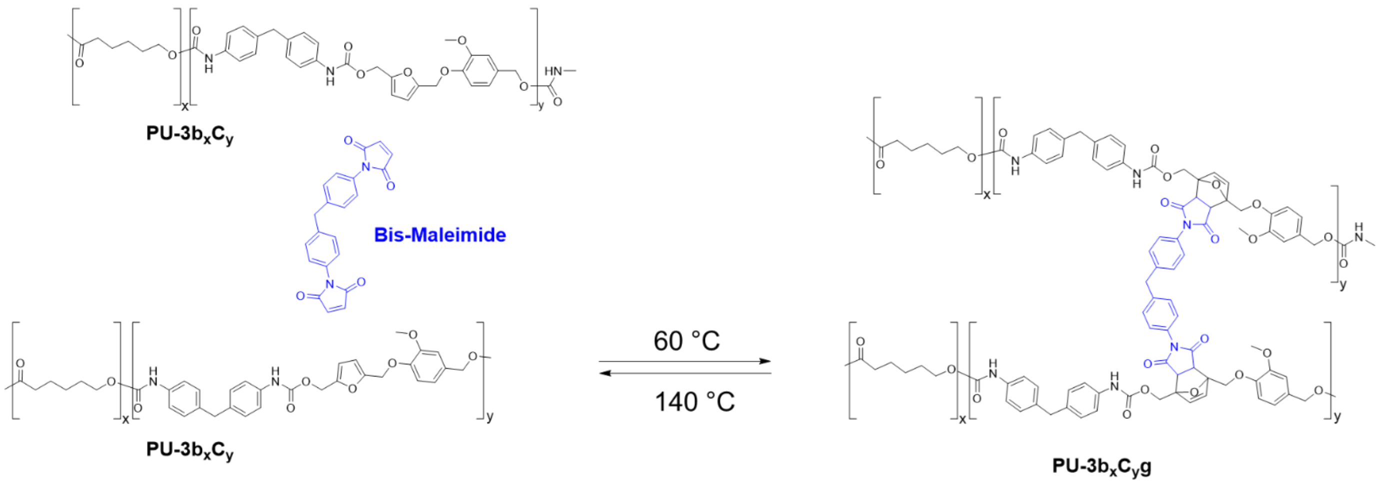

Scheme S1. Reversible Crosslinking of PU-3b$_x$C$_y$ with bis-maleimide using a reversible Diels-Alder reaction.

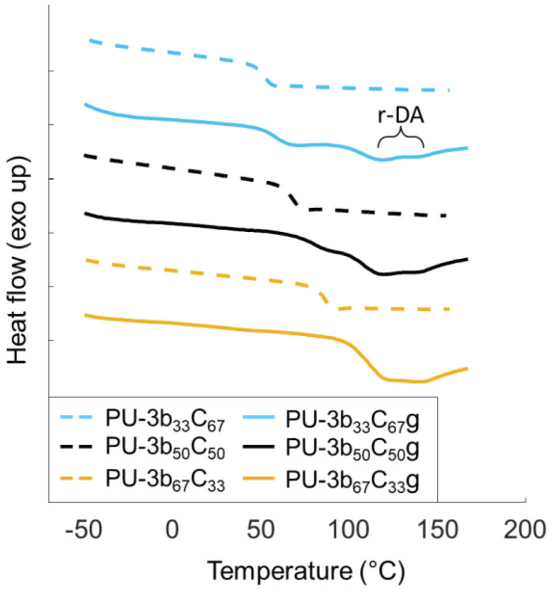

Figure S52. DSC traces from the 2nd heating cycle of the copolymers before and after crosslinking.

Table S12. Physical properties of the thermoplastic polyurethanes.

	E' (MPa) [a]	E' (MPa) [b]	E' (MPa) [c]	T_g (°C) [d]	T_g (°C) [e]
**PU-3b**	-	-	-	110	-
**PU-3b$_{67}$C$_{33}$**	1910	2134	38	86	77
**PU-3b$_{50}$C$_{50}$**	2120	2010	12	69	46
**PU-3b$_{33}$C$_{67}$**	2146	1177	9	53	24
**PU-C**	-	-	-	17	-

[a] Storage modulus taken 30 °C below T_g. [b] Storage modulus taken at room temperature. [c] Storage modulus taken at the rubbery plateau (40 °C above T_g). [d] T_g measured by DSC. [e] T_g measured by DMA as the maximum in E'' curves.

Table S13. Thermal stability of the copolyurethanes.

	TGA	
Polymer	T_5 [a] (°C) (Non-Crosslinked)	T_5 [b] (°C) (Crosslinked)
**PU-3b$_{33}$C$_{67}$**	236	232
**PU-3b$_{50}$C$_{50}$**	225	224
**PU-3b$_{67}$C$_{33}$**	218	221

[a] T_5 was measured by TGA as the temperature at 5% mass loss for the non-crosslinked polymers. [b] T_5 was measured by TGA as the temperature at 5% mass loss for the polymers crosslinked with bis-maleimide.

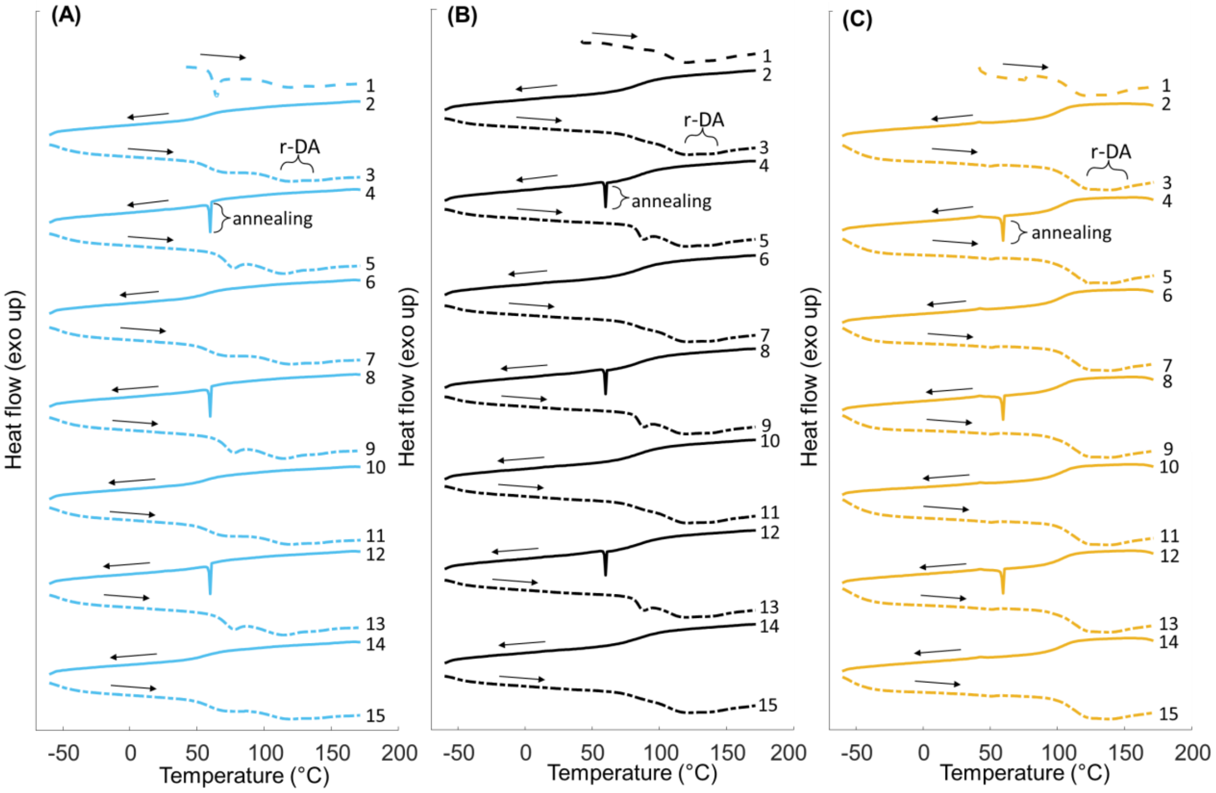

Figure S53. DSC traces of the cross-linked polymers **PU-3b$_{33}$C$_{67}$g** (A), **PU-3b$_{50}$C$_{50}$g** (B), and **PU-3b$_{67}$C$_{33}$g** (C).

Table S14. T_gs measured from the different heating cycles for the DSC experiment.

	**PU-3b$_{33}$C$_{67}$g**		**PU-3b$_{33}$C$_{67}$g-R**		**PU-3b$_{50}$C$_{50}$g**		**PU-3b$_{67}$C$_{33}$g**	
Cycle	T_g gel [a] (°C)	T_g Annealed [b] (°C)	T_g gel [a] (°C)	T_g Annealed [b] (°C)	T_g gel [a] (°C)	T_g Annealed [b] (°C)	T_g gel [a] (°C)	T_g Annealed [b] (°C)
1	59	72	60	71	83	85	110	114
2	60	72	58	72	85	85	114	113
3	59	72	57	71	83	85	115	116
4	60		59		80		114	

[a] T_g measured from cycle 3, 7, 11, and 15. [b] T_g measured from cycle 5, 9, and 13.

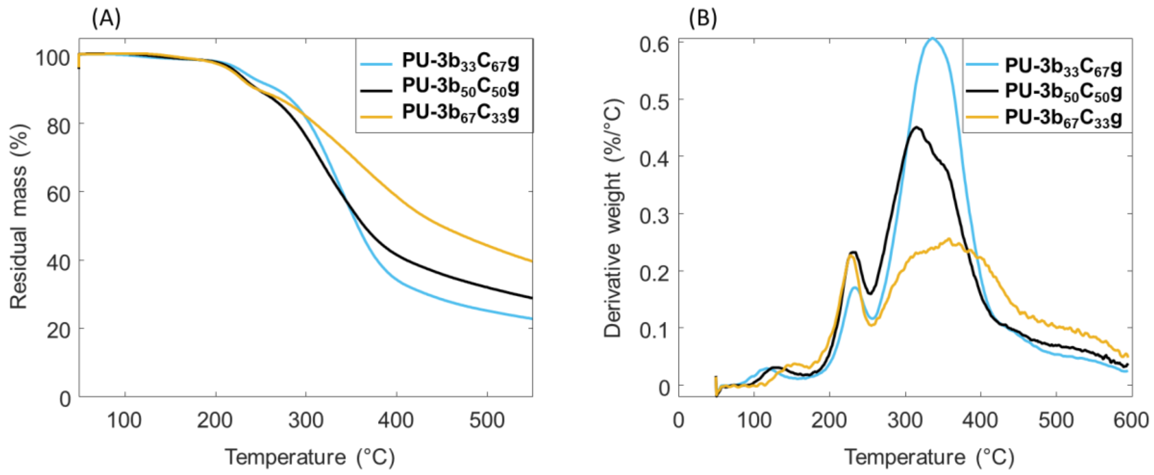

Figure S54. (A) TGA thermograms and (B) 1st derivative curves of the crosslinked polyurethanes.

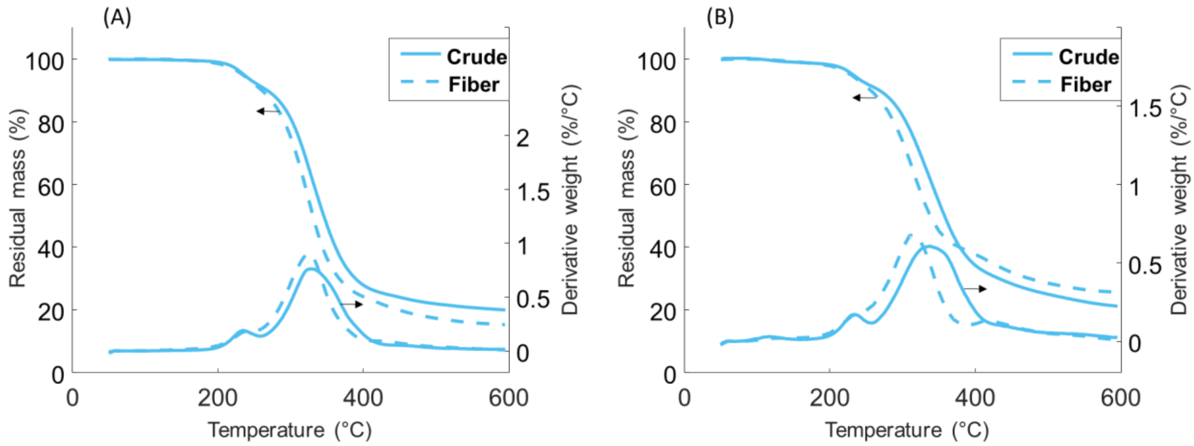

Figure S55. TGA thermograms and 1st derivative curves of (A) **PU-3b$_{33}$C$_{67}$** and (B) **PU-3b$_{33}$C$_{67}$g** before and after fiber spinning. Solid curves are before fiber spinning, and dashed curves are after fiber spinning (indicated as **Crude** and **Fiber**).

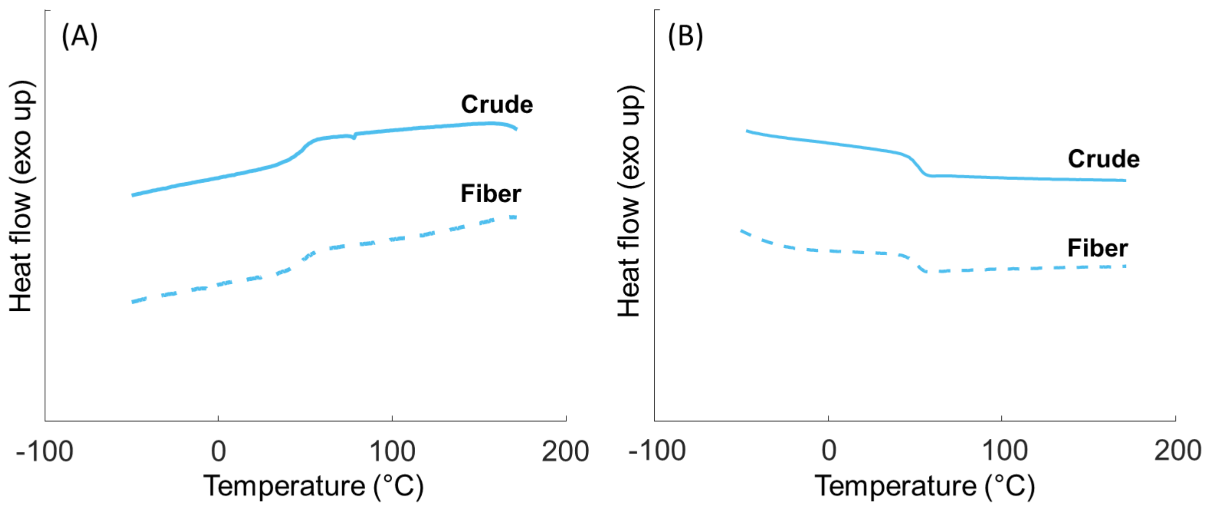

Figure S56. DSC (A) first cooling and (B) second heating curves of **PU-3b$_{33}$C$_{67}$** before and after fiber spinning**.** Solid curves are before fiber spinning and dashed curves are after fiber spinning (indicated as **Crude** and **Fiber**).

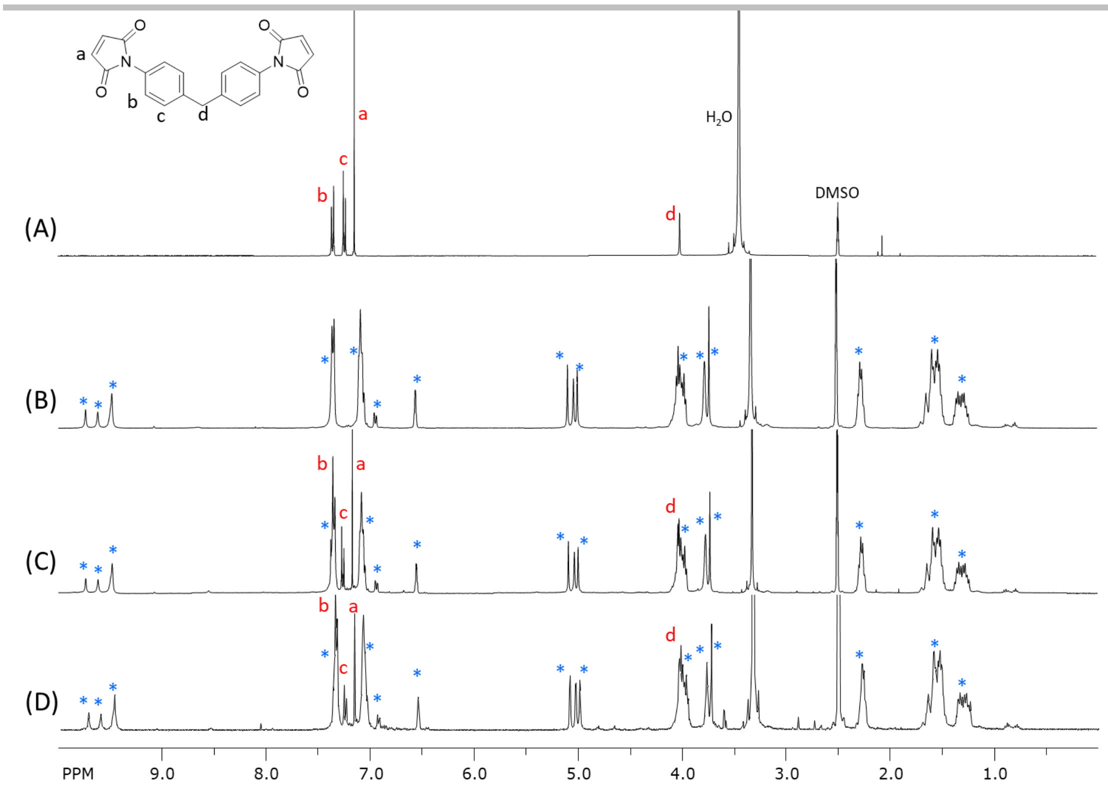

Figure S57. ^{1}H-NMR spectra of bis-maleimide (A), non-crosslinked **PU-3b$_{33}$C$_{67}$** polymer fiber (B), the regenerated polymer from the crosslinked fiber (**PU-3b$_{33}$C$_{67}$g**) after heating in DMSO-*d*$_6$ (C), and the regenerated polymer from the crosslinked fiber (**PU-3b$_{33}$C$_{67}$g-R**) after heating in DMSO-*d*$_6$ (D).

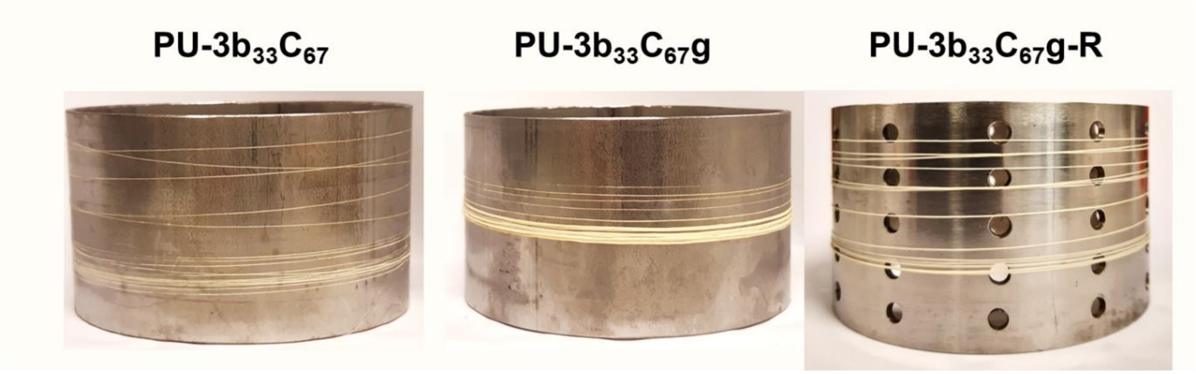

Figure S58. Wet spun fibers of **PU-3b$_{33}$C$_{67}$**, **PU-3b$_{33}$C$_{67}$g**, and **PU-3b$_{33}$C$_{67}$g-R.**

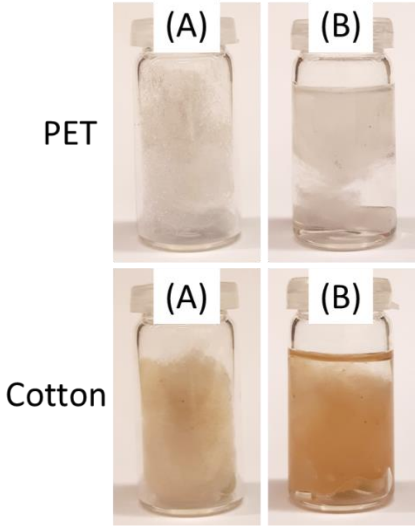

Figure S59. Solubility test of PET and cotton fibers in DMF. (A) Dry PET and cotton fibers. (B) PET and cotton fibers after being heated to 140 °C for 1 h in DMF.

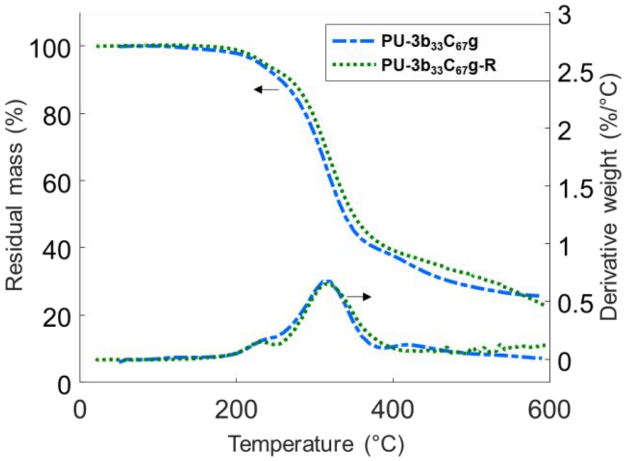

Figure S60. TGA thermograms and 1st derivative curves of **PU-3b$_{33}$C$_{67}$g** and **PU-3b$_{33}$C$_{67}$g-R** fibers.

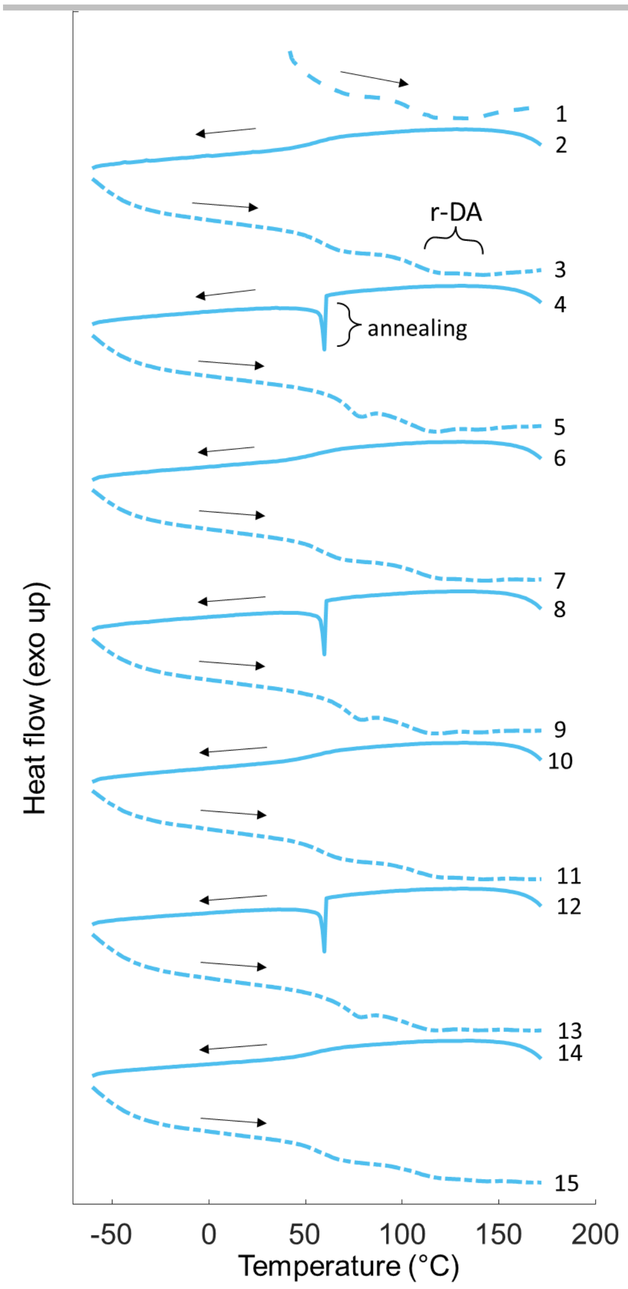

Figure S61. Specially designed DSC measurements of the fiber made from crosslinked polymer **PU-3b$_{33}$C$_{67}$g**. Annealing was conducted during the cooling cycles (No. 4, 8, 12) at 60 °C before further cooling down. Arrows on top of each curve indicate whether they are heating or cooling.

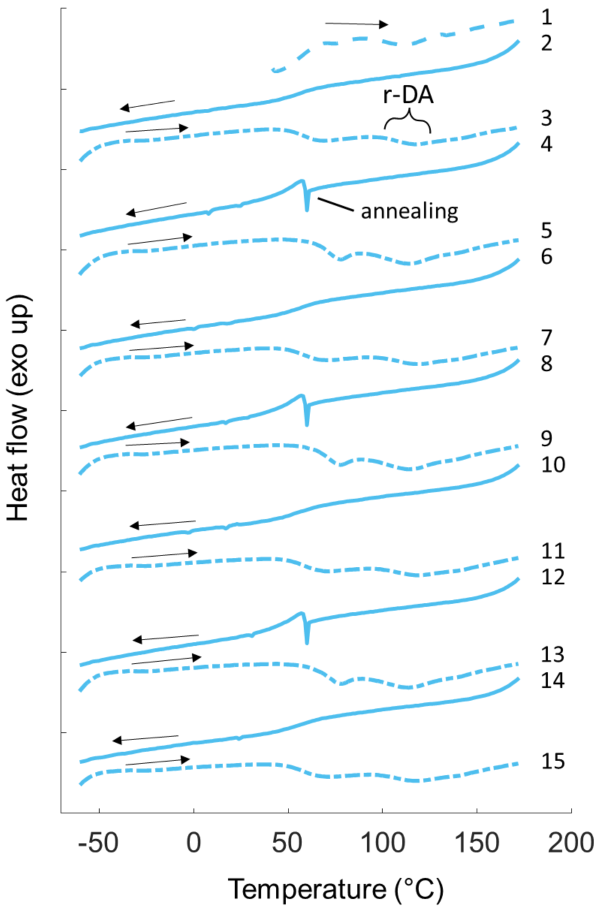

Figure S62. Specially designed DSC measurements of the fiber **PU-3b$_{33}$C$_{67}$g-R**. Annealing was conducted during the cooling cycles (No. 4, 8, 12) at 60 °C before further cooling down. Arrows on top of each curve indicate whether they are heating or cooling.

Table S15. Thermal properties of the polymers before and after fiber spinning.

Polymer	T_5 [a] (°C)	T_g [b] (°C)
PU-3b$_{33}$C$_{67}$	236	53
PU-3b$_{33}$C$_{67}$g	235	72
PU-3b$_{33}$C$_{67}$g fiber	229	72
PU-3b$_{33}$C$_{67}$g-R fiber	235	71

[a] T_5 was taken as the temperature at 5% mass loss for the copolyurethanes. [b] T_g of the polymers measured by DSC second heating curves.

Table S16. Mechanical properties of the obtained fibers.

Polymer	Draw ratio	Titer dtex	Force cN	Elong. %	Ten. cN/tex	YM (1%) cN/tex
Cotton	-	**1.4**±0.43	**6.3**±2.8	**8.9**±2.6	**43.8**±14.8	**524.7**±171.6
Elastane	-	**64.1**±2.5	**77.6**±4	**624.4**±33.4	**12.1**±0.9	**16.4**±0.8
PET	-	**9.4**±0.5	**36.7**±1	**48**±6.7	**39**±2.3	**109.0**±31.3
**PU-C**[a]	1	**35.1**±0	**52.3**±16.7	**382.7**±56.7	**14.9**±4.5	**14.9**±0.8
**PU-3$b_{33}C_{67}$**[b]	1	**78.5** ± 2.5	**27.2**±0.7	**72.5**±24.9	**3.4**±0.1	**173.3**±3.8
**PU-3$b_{33}C_{67}$**[b]	1.2	**84.4**±1.8	**40.3**±2.6	**295.7**±15.1	**4.8**±0.3	**129.7**±19.2
**PU-3$b_{33}C_{67}$g**[c]	1	**70.5**±5.6	**39.1**±3.5	**79.4**±63.5	**5.6**±0.9	**222.5**±21.1
**PU-3$b_{33}C_{67}$g**[c]	1.2	**67.5**±6.5	**52.4**±3.5	**188.7**±11.8	**7.8**±0.5	**219.4**±9.0
**PU-3$b_{33}C_{67}$g-R**[d]	1	**72.3**±4.5	**29.5**±4.6	**11.8**±6.3	**4.1**±0.5	**191.5**±9.3

[a] Fiber spun from **PU-C.** [b] Fiber spun from **PU-3$b_{33}C_{67}$**. [c] Fiber spun from **PU-3$b_{33}C_{67}$g**. [d] Fiber spun from **PU-3$b_{33}C_{67}$g** recycled from PET and cotton mixture.

References

[1] *ISO - ISO 14040:2006 - Environmental Management — Life Cycle Assessment — Principles and Framework*, **n.d.**

[2] *ISO 14044:2006(En), Environmental Management — Life Cycle Assessment — Requirements and Guidelines*, **n.d.**

[3] M. Mascal, E. B. Nikitin, *ChemSusChem* **2009**, *2*, 859–861.

[4] M. Brasholz, K. Von Känel, C. H. Hornung, S. Saubern, J. Tsanaktsidis, *Green Chem.* **2011**, *13*, 1114.

[5] H. H. Szmant, D. D. Chundury, *J. Chem. Technol. Biotechnol.* **1981**, *31*, 205–212.

[6] M. Viskovic, D. Djatkov, M. Martinov, *J. Clean. Prod.* **2018**, *199*, 383–390.

[7] I. Tsiropoulos, B. Cok, M. K. Patel, *J. Clean. Prod.* **2013**, *43*, 182–190.

[8] M. N. García González, P. Börjesson, M. Levi, S. Turri, *J. Polym. Environ.* **2018**, *26*, 3626–3637.

[9] M. N. Garcia Gonzalez, L. Björnsson, *J. Clean. Prod.* **2022**, *346*, 131211.

[10] M. Sayed, N. Warlin, C. Hulteberg, I. Munslow, S. Lundmark, O. Pajalic, P. Tunå, B. Zhang, S.-H. Pyo, R. Hatti-Kaul, *Green Chem.* **2020**, *22*, 5402–5413.

[11] J. Wang, W. Xu, J. Ren, X. Liu, G. Lu, Y. Wang, *Green Chem.* **2011**, *13*, 2678–2681.

[12] "α-D-Glucose," can be found under https://webbook.nist.gov/cgi/cbook.cgi?ID=C492626&Mask=2, **n.d.**

[13] S. P. Verevkin, V. N. Emel'yanenko, E. N. Stepurko, R. V Ralys, D. H. Zaitsau, A. Stark, *Ind. Eng. Chem. Res.* **2009**, *48*, 10087–10093.

[14] Y. Zhou, J. Wu, E. W. Lemmon, *J. Phys. Chem. Ref. Data* **2011**, *40*, 1–11.

[15] K. S. Pitzer, *J. Am. Chem. Soc.* **1940**, *62*, 331–335.

[16] "Toluene," can be found under https://webbook.nist.gov/cgi/cbook.cgi?ID=C108883&Mask=2, **n.d.**

[17] "Sucrose," can be found under https://webbook.nist.gov/cgi/cbook.cgi?ID=C57501&Mask=2, **n.d.**

[18] "Fructose (CAS 7660-25-5) - Chemical & Physical Properties by Cheméo," can be found under

https://www.chemeo.com/cid/56-374-5/Fructose#ref-nist-webbook, **n.d.**

[19] G. H. Aylward, T. J. V. (Tristan J. V. Findlay, *SI Chemical Data*, John Wiley & Sons Australia, **2007**.

[20] "Methylene chloride," can be found under https://webbook.nist.gov/cgi/cbook.cgi?ID=C75092&Units=CAL&Mask=2, **n.d.**

[21] M. Mascal, E. B. Nikitin, *Angew. Chemie - Int. Ed.* **2008**, *47*, 7924–7926.

[22] A. H. Johnstone, *J. Chem. Technol. Biotechnol.* **2007**, *50*, DOI 10.1002/jctb.280500215.

[23] R. Itten, R. Frischknecht, M. Stucki, *Life Cycle Inventories of Electricity Mixes and Grid*, **2012**.

Author Contributions

Conceptualization: NW, MNGG, OYA, FGB, ZG, SL, BZ

Methodology: NW, MNGG, FGB

Validation: NW, MNGG, OYA

Formal Analysis: NW, MNGG

Investigation: NW, RNLM, MNGG, AK, EO, CA, FGB

Resources: NW, MNGG, MS, OYA, ZG, NR, RHK, PJ, BZ

Writing – Original Draft: NW

Writing – Review & Editing: NW, MNGG, RNLM, AK, EO, CA, MS, SVM, NV, OYA, CPH, FGB, ZG, NR, SL, RHK, PJ, BZ

Visualization: NW, SVM, NV, BZ

Supervision: NW, CPH, RHK, PJ, BZ

Project Administration: NW, CPH, RHK, PJ, BZ

Funding acquisition: NW, CPH, ZG, NR, RHK, PJ, BZ
